# Unexpected conservation of the RNA splicing apparatus in the highly streamlined genome of *Galdieria sulphuraria*

**DOI:** 10.1186/s12862-018-1161-x

**Published:** 2018-04-02

**Authors:** Huan Qiu, Alessandro W. Rossoni, Andreas P. M. Weber, Hwan Su Yoon, Debashish Bhattacharya

**Affiliations:** 1 Department of Ecology, Evolution and Natural Resources, Rutgers University, New Brunswick, NJ 08901 USA; 2 Institute for Plant Biochemistry, Cluster of Excellence on Plant Sciences (CEPLAS), Heinrich-Heine-University, D-40225 Düsseldorf, Germany; 3 Department of Biological Sciences, Sungkyunkwan University, Suwon, 16419 Korea; 4 Department of Biochemistry and Microbiology, Rutgers University, New Brunswick, NJ 08901 USA

**Keywords:** Genome reduction, RNA splicing, Intron, Rhodophyta

## Abstract

**Background:**

Genome reduction in intracellular pathogens and endosymbionts is usually compensated by reliance on the host for energy and nutrients. Free-living taxa with reduced genomes must however evolve strategies for generating functional diversity to support their independent lifestyles. An emerging model for the latter case is the Rhodophyta (red algae) that comprises an ecologically widely distributed, species-rich phylum. Red algae have undergone multiple phases of significant genome reduction, including extremophilic unicellular taxa with limited nuclear gene inventories that must cope with hot, highly acidic environments.

**Results:**

Using genomic data from eight red algal lineages, we identified 155 spliceosomal machinery (SM)-associated genes that were putatively present in the red algal common ancestor. This core SM gene set is most highly conserved in *Galdieria* species (150 SM genes) and underwent differing levels of gene loss in other examined red algae (53-145 SM genes). Surprisingly, the high SM conservation in *Galdieria sulphuraria* coincides with the enrichment of spliceosomal introns in this species (2 introns/gene) in comparison to other red algae (< 0.34 introns/gene). Spliceosomal introns in *G. sulphuraria* undergo alternatively splicing, including many that are differentially spliced upon changes in culture temperature*.*

**Conclusions:**

Our work reveals the unique nature of *G. sulphuraria* among red algae with respect to the conservation of the spliceosomal machinery and introns. We discuss the possible implications of these findings in the highly streamlined genome of this free-living eukaryote.

**Electronic supplementary material:**

The online version of this article (10.1186/s12862-018-1161-x) contains supplementary material, which is available to authorized users.

## Background

The study of eukaryote genome evolution has focused primarily on how genomes grow in size and complexity over time (e.g., via genome duplication [1] and transposable element accumulation [2] often due to neutral, population level processes) in model organisms such as vertebrates and land plants. In contrast, there is limited information arising from the opposite perspective (i.e., genome reduction), despite its prevalence in many lineages [3]. In addition, knowledge about genome reduction, which has been studied primarily in highly specialized endosymbionts and pathogens [4] has limited implications for free-living species and the maintenance of their biodiversity. Therefore, understanding the impact of genome reduction in free-living organisms, particularly in eukaryotes that have complex genomes, provides a novel avenue to understand and test the underlying principles of genome evolution.

An emerging model for elucidating the impacts of genome reduction in free-living eukaryotes is the Rhodophyta (red algae). This monophyletic algal lineage comprises an ecologically widely distributed and species-rich phylum (ca. 7000 species) [5]. Analysis of genomic and transcriptomic data have shown that red algae underwent at least two phases of massive genome reduction [6]. The first is in the stem lineage, where about one-quarter of the gene inventory was shed [6] and the second is in the ancestor of the anciently diverged extremophiles, Cyanidiophytina, such as *Cyanidioschyzon merolae* [7] and *Galdieria sulphuraria* [8], that thrive in volcanic hot-spring areas [6, 9]. As a consequence of adaptation to their unusual environment, *G. sulphuraria* (6.5 K nuclear genes) and *C. merolae* (4.7 K nuclear genes) contain smaller gene inventories than their mesophilic red algal sisters which encode ~ 10 K nuclear genes [10–12].

Alternative splicing provides a major avenue of post-transcriptional regulation in eukaryotes [13]. Here, using analysis of genomic and RNA-seq data from *G. sulphuraria*, we show: 1) selective retention of the spliceosomal machinery (SM) in *G. sulphuraria*, a toolkit that has been greatly reduced in complexity in many of its sister red algal lineages, and 2) the coincidence of high SM retention and intron enrichment in *G. sulphuraria* that has resulted in extensive alternative splicing (AS) in this species. Given these unique features in *G. sulphuraria*, we discuss the possible implications of AS in red algal evolution.

## Results

### Pattern of spliceosome machinery gene loss in red algae

**Fig. 1:**
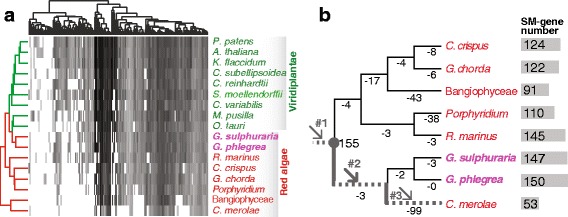
The evolution of SM genes in red algae. **a** Distribution of 215 spliceosomal (SM) protein homologs in 17 red algal (in red color) and Viridiplantae lineages (in green color). *Galdieria* species are shown in the purple color. The heatmap is based on the sequence identities between human SM proteins and their homologs in the corresponding species. **b** The putative losses of SM genes in the red algal tree of life. The extent of SM gene loss is indicated by the numbers under branches. The grey circle indicates the most recent common ancestor of red algae. The dashed branches and arrows indicate the three phases of red algal genome reduction

Using a BLASTp search-based method (see Methods) with 215 non-redundant human SM-associated proteins [14] as the query, we identified homologs in red algae and their putative sister lineage, the Viridiplantae (Additional file 1: Table S1). Consistent with the fundamental function of the SM, a majority of these proteins have detectable homologs in red algae and Viridiplantae (Fig. 1a), with generally more genes found in the latter phylum (Fig. 1a). Substantial variation in SM gene number was found among red algal lineages with *Galdieria* species (*G. sulphuraria* and *G. phlegrea*) containing the largest number of genes and *C. merolae* the smallest (Fig. 1a); the latter result has previously been described [15]. The observed SM gene distribution among red algal species could have resulted from independent, recent gene losses in multiple lineages or from extensive gene acquisition via horizontal gene transfer (HGT; e.g., in *G. sulphuraria* [8]). To distinguish between these two scenarios, we used phylogenetics to study the origin of red algal SM genes (see Methods) and estimated the timing of SM gene losses using a robust red algal tree of life [16]. Most individual SM gene phylogenies suggest vertical transmission because of the shared common ancestry of red algae with a variety of other eukaryotes (e.g., Metazoa in Additional file 2: Figure S1A; see Additional file 3 for all of the phylogenies). No clear evidence was found for the HGT of SM genes in *Galdieria* and other red algal species (Additional file 3). Using Dollo parsimony [17], we reconstructed the evolutionary history of SM genes in red algae. A total of 155 SM associated genes was likely present in the stem lineage of red algae, most of which (150) are preserved in *Galdieria* species (Fig. 1b). In contrast, extensive SM gene losses occurred independently in other red algal lineages such as *C. merolae* (currently 53 SM genes), Bangiophyceae (*Porphyra yezoensis* + *Porphyra umbilicalis*, 91 SM genes), and *Porphyridium* species (*P. purpureum* and *P. aerugineum*, 110 SM genes) (Fig. 1a). *Rhodosorus marinus* (145) contains a SM gene number similar to that in *Galdieria* species (Fig. 1b). Using 303 highly conserved gene families in eukaryotes as reference, we assessed the completeness of each red algal protein dataset with BUSCO. Most species showed a high coverage (< 8% missing genes), except *Chondrus crispus* (16% missing) and Bangiophyceae (19% missing) (Additional file 4: Table S2). Because *C. crispus* contains slightly more SM-genes than its sister lineage *Gracilariopsis chorda*, this result suggests that the estimate of SM-gene number in most species was robust except for the Bangiophyceae. Whereas the extensive SM gene loss in *C. merolae* is likely explained by recent genome reduction specific to this extremophilic lineage [9] (arrow #3 in Fig. 1b), the underlying reasons for SM gene loss in *Porphyridium* and other mesophilic species are unclear. In contrast, the significant retention of SM genes at the split of extremophilic and mesophilic red algae (and maintenance in *Galdieria*) in the face of genome reduction, specific to this group (arrow #2 in Fig. 1b) is a surprising result.

### Spliceosome composition and conservation in red algae

Based on human SM protein expression data [14] (Additional file 5: Table S3), we found that highly expressed SM proteins (79%) are twice as likely to be retained in red algae as those expressed at low levels (43%). Nearly all of the core proteins that directly bind small nucleolar (sn) RNAs to form small nuclear ribonucleoproteins (i.e., Sm, U1, U2, U5, U4/U6 and U5/U4/U6) are conserved in *Galdieria* species and have the lowest degree of loss in mesophilic red algae (Additional file 5: Table S3). All five snRNAs (U1-2 and U4-6) are found in *G. sulphuraria* (Additional file 6: Figure S2) and in five other red algal genomes (Additional file 7: Table S4), except for U1 snRNA that was most likely lost in *C. merolae* [15] (Additional file 7: Table S4). Whereas we included *G. phlegrea* to fully capture the SM gene inventory (Fig. 1), this taxon is not included in downstream analyses because of its close phylogenetic relationship to *G. sulphuraria* and the relatively low quality of intron annotation due to a lack of transcriptome data. In contrast, the remaining auxiliary SM proteins that generally perform peripheral or modulatory functions underwent more frequent loss (Additional file 5: Table S3). These results suggest that the red algal common ancestor contained 155 SM proteins that comprised the complete core of the spliceosomal machinery that was largely maintained in some lineages (such as *Galdieria*). It is noteworthy that although *C. merolae* and *G. sulphuraria* are both extremophiles that inhabit areas surrounding volcanic hot springs, these two species differ dramatically in lifestyle and metabolic capacity [18] which presumably is reflected by the additional phase of genome reduction in *C. merolae* (arrow #3 in Fig. 1b) [9]. Whereas the extremely reduced SM does perform RNA-splicing functions in *C. merolae*, it likely has a highly compromised efficiency given the minimal number of introns (only 27) present in this genome [7]. A highly reduced SM has also been found in several parasitic eukaryotes [19]. These species invariably show a paucity of introns including the kinetoplastid *Trypanosoma brucei* (13 introns in 8747 genes) [20], the microsporidian *Encephalitozoon cuniculi* (7 introns in 1996 genes) [21], and the diplomonad *Giardia lamblia* (6 introns in 7364 genes) [22].

Consistent with a previous study [15], we found that many red algal SM proteins are distantly related to reference sequences (i.e., human SM proteins) and have extremely long branches in phylogenies (Additional file 8: Figure S3). This pattern of evolution is common in red algal species with reduced SM gene sets such as *C. merolae*, Bangiophyceae, and *Porphyridium*, and is largely absent in *G. sulphuraria* and *R. marinus*. Using the BLASTp bit score as the metric, we found *G. sulphuraria* SM proteins to be generally more conserved than their orthologs in other red algal species (except *R. marinus*) at the primary sequence level (Additional file 8: Figure S3). Similarly, *G. sulphuraria* also shows the strongest overall sequence conservation among snRNAs, as reflected by their high alignment scores (Additional file 7: Table S4). *C. merolae* has the least conserved snRNAs (Additional file 7: Table S4). Whereas the fast evolution of SM proteins and snRNAs might reflect a genome-wide feature in *C. merolae* [16], the highly derived SM proteins and snRNAs in other red algal species likely resulted from the acquisition of novel functions or relaxed functional constraints. This result suggests that some apparent cases of gene loss in SM gene-poor red algal species (e.g., *C. merolae*) might instead be explained by high divergence; i.e., beyond sequence similarity-based recognition. In summary, our results demonstrate the conservation of the *G. sulphuraria* SM with respect to both gene inventory and protein similarity.

### Enrichment of introns in the *G. sulphuraria* genome

**Fig. 2:**
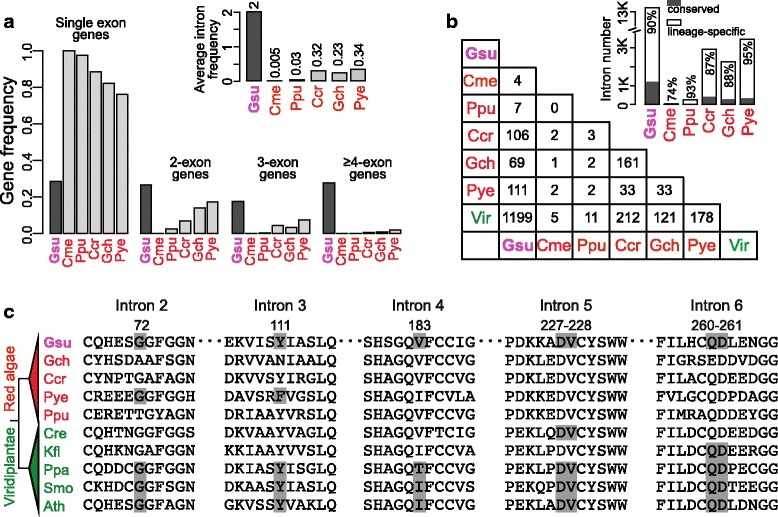
Evolution of red algal introns. **a** The frequencies of single-exon genes and multi-exon genes in six red algal nuclear genomes. The bars in dark grey indicate *G. sulphuraria*. Abbreviations: Gsu (*G. sulphuraria*), Cme (*C. merolae*), Ppu (*P. purpureum*), Ccr (*C. crispus*), Gch (*G. chorda*), Pye (*P. yezoensis*) and Vir (Viridiplantae). The color scheme is the same as in Fig. 1. **b** Shared intron positions across algal lineages. The frequencies of lineage-specific intron for each red algal lineage are shown. **c** Conserved intron positions in *G. sulphuraria* geranylgeranyl transferase beta-subunit gene. The residues interrupted by intron (phase 2 and 3) or flanking intron (phase 1) are shadowed. The alignment is shown only for the 11-12 residue segments spanning the conserved intron positions. The positions of amino acids refer to those in *G. sulphuraria* proteins. Introns found only in Viridiplantae are not shown. Full alignment is provided in Additional file 11: Figure S5

Among the six red algal species with completed or draft genomes, intron numbers vary substantially, ranging from 27 in *C. merolae*, 245 in *P. purpureum*, to 13,245 in *G. sulphuraria* (Additional file 9: Table S5). The most highly conserved red algal SM in *G. sulphuraria* (Fig. 1) coincides with the enrichment of introns and multiple-exon genes in this species (Fig. 2a). Conversely, *C. merolae* that has the most reduced SM (Fig. 1) possesses the smallest number of introns (Fig. 2a and Additional file 9: Table S5). On average, *G. sulphuraria* genes are interrupted by two introns, whereas the corresponding numbers in other four red algal genomes are markedly smaller (0.005 – 0.3 intron/gene, Fig. 2a). The number of genes with one or more introns in *G. sulphuraria* greatly exceeds that in the other five studied red algal species (Fig. 2a). Whereas intron number is likely underestimated in the *P. yezoensis* genome because of its highly fragmented assembly [12], our conclusion does not change when the intron estimate is derived from a set of ‘complete’ *P. yezoensis* genes (i.e., 60% single-exon gene and 0.7 intron/gene on average [12]). Although in need of validation with additional genome data, these results suggest that the extent of SM conservation is likely associated with intron density in red algal genomes (Additional file 10: Figure S4A). A high number of auxiliary SM genes in *G. sulphuraria* likely results in an efficient SM that is able to process the relatively large number of introns in this species. Notably, *G. sulphuraria* has an exceptionally low GC content among red algae (Additional file 10: Figure S4B). Additional red algal genomic data are required to test the correlation between GC content and intron density in these taxa (Additional file 10: Figure S4C).

### Origin of *G. sulphuraria* introns

To study the origin of *G. sulphuraria* spliceosomal introns, we compared their positions within homologous genes across six red algal species (Fig. 2b) and between them and five Viridiplantae lineages (Additional file 9: Table S5). Most of the *G. sulphuraria* intron positions (90%) appear to be lineage-specific (Fig. 2b), likely resulting from recent intron insertions. Based on a self-BLASTn search (*e*-value cutoff = 1e-5), only 3.6% (478/13,245) of introns share sequence similarity (query coverage ≥0.5) with one or more (up to 9) other introns. This result does not support the idea that the majority of *G. sulphuraria* introns resulted from recent intron duplications. Regarding intron positions that are shared with Viridiplantae, *G. sulphuraria* (1199) contains > 4-fold more ancestral introns than do other red algal lineages, such as *C. crispus* (212) and *G. chorda* (121) (Fig. 2b). This result suggests that many anciently derived introns were retained in *G. sulphuraria* and lost in other red algal lineages. Examples include the intron-rich *G. sulphuraria* gene encoding geranylgeranyl transferase beta-subunit (NCBI GeneID: 17088310). This gene contains six introns, of which five (from the 2nd to the 6th) have conserved positions in Viridiplantae homologs (Fig. 2c and Additional file 11: Figure S5). In contrast, all of these introns underwent losses in mesophilic red algae, resulting in a 3-exon gene in *P. yezoensis*, single-exon genes in *P. purpureum*, *C. crispus*, and *G. chorda* (Fig. 2c and Additional file 11: Figure S5). When assuming a simple evolutionary scenario (i.e., Dollo parsimony [17]), about 1700 introns are estimated to have been present in the red algal stem lineage, followed by significant losses in mesophilic red algae and in *C. merolae* (Additional file 12: Figure S6). These results suggest that red algal introns have a high turnover rate, that is a common feature of many eukaryotes [23]. The relatively large number of introns in *G. sulphuraria* resulted from both lineage-specific intron gains and retention of ancestral introns. Notably, *G. sulphuraria* introns are much smaller in size (50 bp, on average) than in other red algal genomes (Additional file 13: Figure S7A). This is consistent with a strong size constraint that has resulted in the compact genome of *G. sulphuraria* (Additional file 13: Figure S7B) [8].

### Alternative mRNA splicing in *G. sulphuraria*

**Fig. 3:**
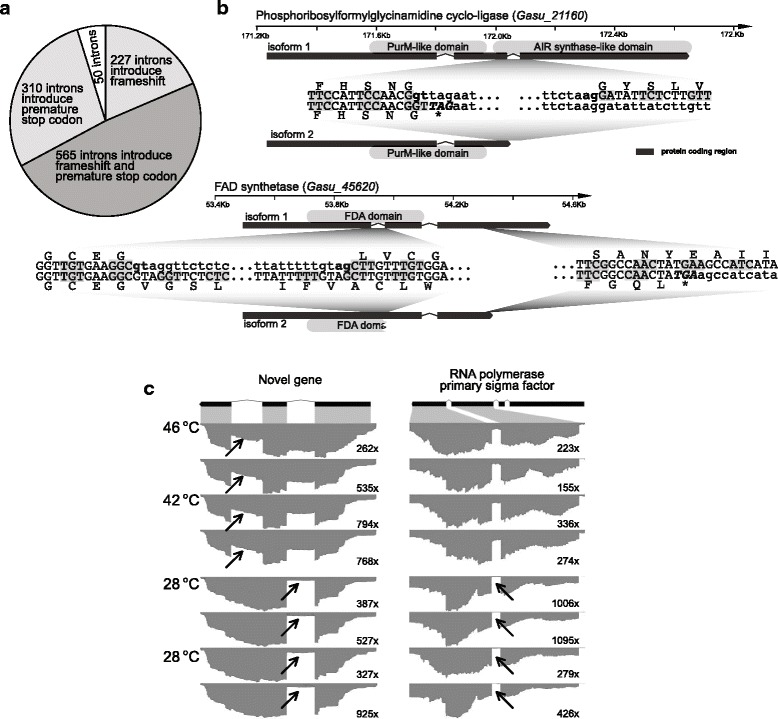
Intron retention in *G. sulphuraria*. **a** Predicted changes resulting from the retention of 1152 alternatively spliced introns identified in this study. **b** Examples of intron retention in two *G. sulphuraria* genes. The sequences flanking the splicing signals (GT-AG) are illustrated. Coding nucleotides are shown in uppercase and non-coding nucleotides in lower case. Gray rectangular boxes are aligned with every other codon triplet. Gene models comprise thin black boxes (exons) connected with angled lines (introns). **c** Two examples of differential intron splicing between the heat and cold conditions in *G. sulphuraria*. Gene structures are shown at the top with the black boxes indicating the exons. For each gene, the read coverage along the displayed regions is shown for a total of 8 samples, with 2 replicates for each at the 4 temperature treatments (see Methods for details). The maximum read coverage is shown for each sample. For each intron, regions that are significantly more spliced than the compared temperature group are indicated with arrows

Why would SM genes and a relatively more complex intron-exon structure be preserved in the compact *G. sulphuraria* genome? The answer to this question may lie in the fact that intron-exon structure provides the foundation in eukaryotes for generating multiple transcripts via alternative splicing. This is true in *G. sulphuraria*, as demonstrated by previous analysis of transcriptome data derived from Sanger sequences and 454 long-reads that revealed alternatively spliced isoforms for about 500 genes [8]. To test if AS in *G. sulphuraria* responds to environmental changes, we generated and analyzed extensive RNA-seq data from this alga under two arbitrary different temperature conditions: ‘heat’ (42 °C and 46 °C; non-stressed, because this alga normally lives at temperatures between 35 and 56 °C [24]) and ‘cold’ (28 °C; stressed) (see Methods). A total of 1766 introns were identified as being alternatively spliced (mostly via intron retention) under one or both temperature conditions (Additional file 14: Table S6). A total of 1397 of these alternatively spliced introns were located within 1027 known *G. sulphuraria* genes, including 12 genes derived via HGT (Additional file 14: Table S6). Among these 1766 introns, 1152 are identical with the annotated *G. sulphuraria* introns, accounting for 10.2% of the latter (13,245 introns). We predicted the impact of retention of these 1152 introns in the encoded transcripts and found that 792 (68.7%) lead to frame-shifts (i.e., in lengths not divisible by 3) (Fig. 3a). When translated in the reading frame of preceding exons; i.e., 875 (75.9%) introns encode premature stop codons that lead to truncated proteins. Only 50 (4.3%) of retained introns do not cause these two types of changes in the inferred proteins. An example of intron retention is provided by the phosphoribosylformylglycinamidine cyclo-ligase gene (NCBI Gene ID: 17089374). The maintenance of its second intron introduces a stop codon (TAG) and leads to a truncated protein with a fragmented AIR synthase-like C-terminal domain (Fig. 3b). In a FDA synthase gene that contains two introns (NCBI Gene ID: 17086779), the retention of the first intron introduces a 1-bp frame-shift resulting in a ~ 150 amino acid novel peptide downstream of the protein encoded by the first exon (Fig. 3b). These two examples are supported by long reads generated by Sanger or 454 sequencing [8]. The functional implication of these protein variants is not yet known.

### Differential intron splicing in *G. sulphuraria*

To test if AS in *G. sulphuraria* responds to temperature fluctuations, we searched and identified 212 introns that were (statistically significantly) differentially spliced between the heat and cold conditions (Additional file 15: Table S7). One example is a novel *Galdieria*-specific gene that is comprised of three exons (Fig. 3c). The splicing of the first intron is largely restricted to the heat condition, whereas the second intron is spliced in both conditions with apparently more extensive intron retention under heat than under cold (Fig. 3c). A second example is the gene encoding the RNA polymerase primary sigma factor (NCBI Gene ID: 17087802), with the first intron being significantly retained (i.e., 1/4~ 1/3 of total transcripts) under the heat condition. The same intron is rarely retained under the cold condition (Fig. 3c). This intron is located within a Sigma70-r3 domain (pfam04539) that is involved in the binding of core RNA polymerase. Retention of this intron leads to stop codons and a truncated Sigma70-r3 domain (Additional file 16: Figure S8). We also found many differentially spliced introns that are not located within any annotated genic region, which likely reflects splicing of non-coding RNA transcripts (Additional file 15: Table S7). Under the same conditions, 178 genes were differentially expressed (> 2.5 fold down- or up-regulated in terms of overall expression abundance) including only two SM genes (Additional file 17: Table S8). Because of splicing variation within a group (i.e., heat) due to differences in treatment (42 °C and 46 °C), our result represents a conservative estimate of the extent of differential splicing between the overall ‘heat’ and ‘cold’ conditions. Although a comprehensive analysis of temperature-dependent gene expression and the functional consequences of differential splicing are not within the scope of this paper, our results suggest that *G. sulphuraria* is able to respond to temperature changes (and likely other stimuli) using differential mRNA-splicing. These results explain the considerable fluctuations in microenvironments where many of these species live (e.g., non-thermophilic *Galdieria soos* [25]). Given the overall reduced SM in red algae, compared to humans and Viridiplantae, the prevalence of intron retention might also have resulted from compromised splicing efficiency in *G. sulphuraria*. How this possible scenario contributes to overall intron retention in *G. sulphuraria* is unknown.

## Discussion

We show here that components of the spliceosomal machinery have undergone recent gene losses and accelerated evolution among different red algal lineages. In this context, the high conservation of the SM in *Galdieria* is counterintuitive, given its relatively more reduced gene inventory resulting from lineage-specific genome reduction (Fig. 1b, arrow #2). In addition, *G. sulphuraria* contains a relatively large number of introns via ancient intron preservation and novel insertions, in spite of its compact genome size. This unexpected evolutionary trajectory allows alternative mRNA splicing that generates transcriptomic (and likely proteomic) diversity [26, 27] in this lineage for about a quarter of the alternatively spliced transcripts that do not encode premature stop codons (including 4.3% leading to insertion of amino acids and 19.7% resulting in novel peptides due to frame-shifts, e.g., Fig. 3b). The functional impact of alternative splicing on *G. sulphuraria* biology remains to be investigated.

It is noteworthy that of the 1152 well annotated, alternatively spliced introns we identified in *G. sulphuraria*, most cases (68.7%) of intron retention lead to a truncated protein (Fig. 3a). Why might this be tolerated? The most likely reason is nonsense-mediated decay (NMD), a process that is widespread in eukaryotes for regulating post-transcriptional gene expression [28]. NMD allows for the targeted degradation of alternatively spliced isoforms that would result in truncated proteins due to the introduction of premature termination codons (PTCs; e.g., due to intron retention), as described previously [29]. NMD is not however completely effective and PTCs persist in the transcript pools of many eukaryotes, suggesting a functional role [30]. Soergel et al. [31] postulated an evolutionary interaction between AS and NMD, that allows the rise of alternative, beneficial splice forms (i.e., under the umbrella of a well-established surveillance system) that can ultimately be fixed in the population. This scenario may provide an explanation for the extensive AS-derived PTCs we found in the *G. sulphuraria* RNA-seq data. Several key genes in NMD (i.e., UPF1-3) are present in *G. sulphuraria* and other red algal species (Additional file 18: Figure S9). Our findings with *G. sulphuraria* are generally in line with existing data from other systems. In *Arabidopsis thaliana*, about 13% of intron-containing genes are potentially regulated by AS/NMD [29]. In the unicellular green alga, *Chlamydomonas reinhardtii*, there are 611 AS events that impact 3% of all genes with intron retention being the most common outcome leading to many PTCs [32]. The AS-derived variants may enhance gene regulation in *G. sulphuraria*. This idea is consistent with the reduced intergenic regions (i.e., that could encode *cis*-regulatory elements) that has resulted from genome streamlining (Additional file 13: Figure S7B).

An additional possible explanation for our results comes from a recent study of the yeast UV stress response where genes associated with transcription are regulated by non-coding RNAs derived from alternatively spliced, short transcripts of the same gene (i.e., alternative last exons (ALEs) [33, 34]). In the case of the *ASCC3* gene that represses RNA polymerase II transcription after UV irradiation, transcription of the complete gene (i.e., full-length protein) is de-repressed by an ALE derived from the same gene that acts as a non-coding regulatory RNA. Therefore, it is possible that stress pathways (i.e., not UV irradiation) impacted by our heat and cold treatments of *G. sulphuraria* may lead to the generation of shorter non-coding RNAs via AS that play a role in regulating the stress response. Thermal stress is clearly a major factor in the ecology of *G. sulphuraria*, therefore AS may produce both novel protein isoforms as well as regulatory RNAs (perhaps like ALEs) that play roles in responding to this stress. More generally, our results suggest that strong constraints that exist on the growth of gene numbers (and functions) due to genome reduction can be ameliorated at the transcriptome level. This insight required the analysis of free-living organisms that have relatively complex genomes (i.e., containing introns) and a history of ancient genome reduction, together with recent lineage-specific gene losses. In this regard, our results underline the utility of free-living taxa such as red algae as models for studying eukaryote genome reduction.

## Conclusions

Our results revealed an unexpected aspect of *Galdieria* genome evolution. Whereas the correlation between SM gene number and spliceosomal intron density within red algae remains to be validated with more genomic data, our findings lead to several hypotheses that can be tested in this unique model to understand genome reduction in free-living organisms.

## Methods

### Detection of spliceosomal proteins in red algae

The culture of *G. sulphuraria* used in this study is the strain with the completed nuclear genome sequence (i.e., 074 W) and was isolated from a site near Reykjavik Island [8]. Using the 215 non-redundant human SM proteins as queries (Additional file 19: Supplementary Methods), we searched the proteomes from eight red algal species and nine Viridiplantae (Fig. 1a and Additional file 1: Table S1) using BLASTp (*e*-value cutoff = 1e-5). Significant hits that led to a reasonable alignment length (query coverage > 30%) and had the highest hit-query identities were recorded for each query versus each search species. The resulting data were clustered and visualized with the heatmap function in the R language. Genes and taxa were clustered using Euclidean distances between all gene (or taxon) pairs and the complete-linkage clustering method.

To search red algal SM with higher stringencies and examine their origins, we adopted a phylogenetic-based method [6]. We generated a proteome data comprising SM proteins derived from homology-based gene predictions (using human proteins as reference; Additional file 19: Supplementary Methods) and protein models annotated in existing studies (Table S1). To identify *G. sulphuraria* SM proteins, we searched the *G. sulphuraria* proteome data with BLASTp (*e*-value cutoff = 1e-3) using human SM proteins as queries. The top three *G. sulphuraria* hits according to bit-score (by default) and the top three hits according to query-hit alignment identity were recorded for further validation. To differentiate between orthologous and paralogous relationships between human SM queries and their *G. sulphuraria* homologs, these proteins were used as queries to search (BLASTp *e*-value cutoff = 1e-5) against a comprehensive local protein database [6]. The significant hits were recorded for each SM query and the representative sequences were selected with up to 8 sequences for each phylum in the default order sorted by bit-score. A second set of representative sequences was selected after re-sorting the BLASTp hits according to the query-hit sequence identity. The two sets of representative sequences (by bit-score and alignment identity) for all the SM queries (human and *G. sulphuraria* homologs potentially corresponding to the same SM gene) were then combined, aligned using MUSCLE (v3.8.31) [35] and trimmed using TrimAl (version 1.2) [36] in automated mode (−automated1). The phylogenetic tree was constructed using FastTree (version 2.1.7) [37] under the ‘WAG+CAT’ model with 4 rounds of minimum evolution SPR moves (−psr 4) and exhaustive ML nearest-neighbor interchanges (−mlacc 2, −slownni). Branch support was derived from the Shimodaira-Hasegawa test [38]. We examine the resulting phylogenies manually. A SM gene was regarded to exist in *G. sulphuraria*, if at least one of the *G. sulphuraria* gene candidates appeared in the same orthologous group as the human SM gene (see Additional file 2: Figure S1A for an example of this approach). The SM gene was considered to be absent if no *G. sulphuraria* candidates were found in the orthologous group with the human SM gene (see Additional file 2: Figure S1B for an example of this approach). HGT was inferred when the candidate red algal sequences were nested within multiple sequences from prokaryotic and/or fungal taxa. Following the same procedure as described above, the presence and absence of SM genes were determined in other red algal lineages that are shown in Fig. 1b.

In addition, we used *Galdieria* SM proteins as references for homology-based gene prediction in genome or transcriptome data from the remaining six red algal species (Fig. 2) (Additional file 19: Supplementary Methods). The resulting SM proteins were incorporated into our comprehensive local protein database described above. Using the *G. sulphuraria* SM proteins (or *G. phlegrea* when the *G. sulphuraria* gene was missing) as queries, we carried out a BLASTp search, sorted the significant hits, selected representative sequences, and aligned and built phylogenetic trees following the procedures described above. The resulting trees were manually inspected to identify additional red algal SM sequences that were monophyletic with the *Galdieria* queries.

### Assessing completeness of the protein data

We used BUSCO (version 3) under the default settings to estimate the overall completeness of protein data (equivalent to genomic coverage) for each red algal species [39]. The ‘Eukaryota sets’ that contained 303 conserved gene families in eukaryotes were used as the reference for this analysis.

### Detection of snRNAs

We downloaded snRNA alignments for U1 (RF00003), U2 (RF00004), U4 (RF00015), U5 (RF00020) and U6 (RF00026) from the Rfam database [40]. The alignments were calibrated (using cmcalibrate) and then used for snRNA searches in red algal genomes (using cmsearch) using Infernal (v1.1.2) with the default settings [41].

### Intron analysis

We downloaded the genome and coding DNA sequences (CDSs) from six red algal species that have high-quality whole genome sequences: *G. sulphuraria* [8], *C. merolae* [7], *P. purpureum* [10], *C. crispus* [11], *P, yezoensis* [12] and *G. chorda* (unpublished data), and from five Viridiplantae species (Table S4). The CDSs were mapped to the corresponding genome sequences using BLAT [42] under the default settings. The non-specific alignments were removed and the positions of introns (in genomes) and exon junctions (in CDSs) were then subtracted from the BLAT output using custom scripts. Most of the introns (94-99%) were flanked by the canonical splicing signal (GT-AG) (Table S4).

To identify *G. sulphuraria* intron positions that are shared with *C. crispus*, we searched *G. sulphuraria* proteins against the *C. crispus* proteome using BLASTp (*e*-value cutoff = 1e-10) and retrieved information about the top 10 BLASTp hits. Because the original intron positions along the protein primary sequences are not comparable across sequences due to variable lengths of N′-terminal domains, insertions, and deletions, we built alignments for each query protein and its corresponding *C. crispus* hit(s) using MUSCLE (v3.8.31) [35]. With gaps being introduced during the alignment procedure, the intron positions in the original sequences (without gaps) were converted into column numbers for each sequence in their respective alignments. A *G. sulphuraria* intron position was considered as being conserved if it was located at the same column position in an alignment with one or more introns of the same phase in *C. crispus*. The same method was used to identify intron positions that were shared between any two of the species included in this study (Fig. 2b).

### *Galdieria sulphuraria* cell cultures

Biological replicate cultures of *G. sulphuraria* 074W were grown separately at 42 °C, constant illumination (90 μE), and constant shaking (160 rpm) in photoautotrophic conditions using 2xGS Medium [43]. The experimental design followed a temperature shift timeline: after two weeks of cultivation at stated conditions the first sampling took place (H-42) and the cultures were swiftly moved to 28 °C. After cold treatment at 28 °C for 48 h, a second sampling was performed (C-28.1). The *G. sulphuraria* was then switched to 46 °C for 48 h, at the end of which a third sample was retrieved (H-46). It again was followed by a cold treatment at 28 °C for 48 h when a fourth sample was retrieved (C-28.2). Altogether, two time-points from high temperatures (42 °C and 46 °C) and two time-points from cold temperatures (28 °C) were targeted for sampling.

### Sequencing of the *Galdieria sulphuraria* transcriptome

RNA was extracted using Roboklon’s Universal RNA Purification Kit by following the “plant tissue samples” protocol (Roboklon, Berlin - Germany). RNA quality and concentration was assessed using a Nanodrop photospectrometer ND-1000 (Peqlab Biotechnologie GmbH, Erlangen -Germany). The samples were synthesized to be compatible with Illumina HiSeq2000 RNA-seq libraries strictly following the "Illumina TruSeq RNA Sample Prep v2 LS Protocol" (Illumina, San Diego - USA). All reagents were scaled by 2/3 to the volume proposed in the protocol. The quality of all libraries was assessed using the Bioanalyzer (Agilent Technologies, Santa Clara - USA). The libraries were sequenced in paired-end mode (2x100bp) on two lanes with an Illumina HiSeq2000 sequencer at the BMFZ (Biologisch-Medizinisches Forschungszentrum, Düsseldorf, Germany). The resulting RNA-seq data were deposited in the NCBI Gene Expression Omnibus (GEO) database (https://www.ncbi.nlm.nih.gov/geo/) under accession number GSE89169.

### Detection of differentially spliced introns

The *G. sulphuraria* RNA-seq data were cleaned in paired-end mode using Trimmomatic (v0.36) [44] to remove contaminated adaptor sequences and low quality regions (SLIDINGWINDOW:6:13). Short reads (< 75 bp) were discarded. The cleaned sequence data were then mapped to *G. sulphuraria* genome sequences using STAR (v2.5.2a) [45]. The genome index was generated taking into account the small size of *G. sulphuraria* genome (−-genomeSAindexNbases 11). The reads were mapped to the genome assembly with an allowed maximum intron size (1000 bp) and maximum mate-pair distance (500 bp). Reads that mapped to more than one region were removed and broken pairs were discarded. The mapping results (in SAM format) were then used as input to search for alternatively spliced modules [46] that were differentially expressed across samples using DiffSplice [46] under the default setting, with the following modifications. We required a splice junction to be considered if the mean coverage across all samples was > 10× and RNA-splicing at the junction was found in at least four out of the eight different samples. The expression thresholds for exons and introns were specified to be 16× and 8× coverage, respectively. For the test of differential splicing, the minimal value for square root of JSD [46] was set to be 0.25 with false discovery rate threshold (=0.01). The minimum fold change (2.5) was required to call gene differential expression (down- or up-regulation). Because we aimed to test the existence of differential intron splicing in response to temperature changes (instead of global gene expression change across samples), we regarded the two samples (H-42 and H-46) as biological replicates from high temperature, and the other two samples (C-28.1 and C-28.2) as biological replicates from low temperature. This practice maximized statistical power to detect splicing events that were shared within groups (e.g., high temperature samples including H-42 and H-46) and differed between the groups (high versus low temperature samples). The examples of alternatively spliced modules that showed statistically significant difference between high and low temperatures (Fig. 3c) were visualized using CLC workbench (v8) (http://www.clcbio.com/products/clc-main-workbench/).

## Additional files

Additional file 1: **Table S1.** Algal genome and transcriptome data used in this study. (PDF 96 kb)

Additional file 2: **Figure S1.** Two examples of spliceosomal single-gene phylogeny that show different ancestries of red algal spliceosomal genes. (PDF 137 kb)

Additional file 3: Phylogenies of red algal SM-genes. (TXT 5414 kb)

Additional file 4: **Table S2.** Completeness of proteomic data estimated using 303 BUSCO gene families that are evolutionarily conserved among eukaryotes. (PDF 119 kb)

Additional file 5: **Table S3.** Presence and absence of human spliceosomal machinery-associated proteins in red algae. (PDF 134 kb)

Additional file 6: **Figure S2.** The search results for snRNA component of the spliceosome in *Galdieria sulphuraria*. (PDF 91 kb)

Additional file 7: **Table S4.** INFERNAL scores and e-values for red algal snRNA genes. (PDF 64 kb)

Additional file 8: **Figure S3.** Sequence conservation in *Galdieria sulphuraria* genes. (PDF 181 kb)

Additional file 9: **Table S5.** The intron statistics in red algal and Viridiplantae genomes. (PDF 71 kb)

Additional file 10: **Figure S4.** GC content and intron density in red algae. (PDF 92 kb)

Additional file 11: **Figure S5.** Conservation of intron positions in the *Galdieria sulphuraria* geranylgeranyl transferase beta-subunit gene. (PDF 97 kb)

Additional file 12: **Figure S6.** Estimation of gains and losses of conserved introns in red algal phylogeny. (PDF 92 kb)

Additional file 13: **Figure S7.** The distributions of intron lengths in five red algal species. (PDF 82 kb)

Additional file 14: **Table S6.** *Galdieria sulphuraria* introns that underwent alternative splicing in our studied samples. (PDF 981 kb)

Additional file 15: **Table S7.** *Galdieria sulphuraria* introns that were differentially spliced under the heat and cold conditions. (PDF 226 kb)

Additional file 16: **Figure S8.** Intron retention in a *Galdieria sulphuraria* gene. (PDF 74 kb)

Additional file 17: **Table S8.** *Galdieria sulphuraria* genes that were differentially expressed under the heat and cold conditions. (PDF 186 kb)

Additional file 18: **Figure S9.** Phylogenetic trees of UPF1, UPF2, and UPF3. (PDF 97 kb)

Additional file 19: Supplementary Methods. (PDF 109 kb)

## Abbreviations

ALE: Alternative last exons
AS: Alternative splicing
CAZyme: Carbohydrate-active enzyme
NMD: Nonsense-mediated decay
PTC: Premature termination codons
SM: Spliceosomal machinery
