# Supplementary material for: Unexpected conservation of the RNA splicing apparatus in the highly streamlined genome of Galdieria sulphuraria
Source: BMC Evol Biol. 2018 Apr 2;18:41. doi: 10.1186/s12862-018-1161-x (PMC5880011; doi:10.1186/s12862-018-1161-x)
Supplement: Supplementary file 1 — Table S1. Algal genome and transcriptome data used in this study. (PDF 96 kb) [file 12862_2018_1161_MOESM1_ESM.pdf]

Table S1. Algal genome and transcriptome data used in this study.

No.	Phylum	Taxa	Data type	Data source
1	Rhodophyta	*Porphyridium aerugineum*[1]	Transcriptome	[1]
2	Rhodophyta	*Porphyridium purpureum*[1]	Genome	[2]
3	Rhodophyta	*Porphyra umbilicalis*[2]	Transcriptome	[3]
4	Rhodophyta	*Pyropia yezoensis*[2]	Genome	[4]
5	Rhodophyta	*Rhodosorus marinus*	Transcriptome	[1]
6	Rhodophyta	*Galdieria phlegrea*	Genome	[5]
7	Rhodophyta	*Chondrus crispus*	Genome	[6]
8	Rhodophyta	*Galdieria sulphuraria*	Genome	[7]
9	Rhodophyta	*Cyanidioschyzon merolae*	Genome	[8]
10	Rhodophyta	*Gracilariopsis chorda*	Genome	Unpublished
11	Viridiplantae	*Arabidopsis thaliana*	Genome	[9]
12	Viridiplantae	*Physcomitrella patens*	Genome	[10]
13	Viridiplantae	*Selaginella moellendorffii*	Genome	[11]
14	Viridiplantae	*Klebsormidium flaccidum*	Genome	[12]
15	Viridiplantae	*Chlamydomonas reinhardtii*	Genome	[13]
16	Viridiplantae	*Coccomyxa subellipsoidea*	Genome	[14]
17	Viridiplantae	*Chlorella variabilis*	Genome	[15]
18	Viridiplantae	*Micromonas pulilla*	Genome	[16]
19	Viridiplantae	*Ostreococcus tauri*	Genome	[17]

[1] Combined to form *Porphyridium* data in Fig. 1A.
[2] Combined to form Banginacea data in Fig. 1A.

References

1. Keeling PJ, Burki F, Wilcox HM, Allam B, Allen EE, Amaral-Zettler LA, et al. The Marine Microbial Eukaryote Transcriptome Sequencing Project (MMETSP): illuminating the functional diversity of eukaryotic life in the oceans through transcriptome sequencing. PLoS Biol. 2014;12:e1001889.

2. Bhattacharya D, Price DC, Chan CX, Qiu H, Rose N, Ball S, et al. Genome of the red alga *Porphyridium purpureum*. Nat. Commun. 2013;4:1941.

3. Chan CX, Blouin NA, Zhuang Y, Zäuner S, Prochnik SE, Lindquist E, et al. *Porphyra* (Bangiophyceae) transcriptomes provide insights into red algal development and metabolism. J. Phycol. 2012;48:1328–42.

4. Nakamura Y, Sasaki N, Kobayashi M, Ojima N, Yasuike M, Shigenobu Y, et al. The first symbiont-free genome sequence of marine red alga, Susabi-nori (*Pyropia yezoensis*). PloS One. 2013;8:e57122.

5. Qiu H, Price DC, Weber APM, Reeb V, Yang EC, Lee JM, et al. Adaptation through horizontal gene transfer in the cryptoendolithic red alga *Galdieria phlegrea*. Curr. Biol. 2013;23:R865-866.

6. Collén J, Porcel B, Carré W, Ball SG, Chaparro C, Tonon T, et al. Genome structure and metabolic features in the red seaweed *Chondrus crispus* shed light on evolution of the Archaeplastida. Proc. Natl. Acad. Sci. U. S. A. 2013;110:5247–52.

7. Schönknecht G, Chen W-H, Ternes CM, Barbier GG, Shrestha RP, Stanke M, et al. Gene transfer from bacteria and archaea facilitated evolution of an extremophilic eukaryote. Science. 2013;339:1207–10.

8. Matsuzaki M, Misumi O, Shin-I T, Maruyama S, Takahara M, Miyagishima S-Y, et al. Genome sequence of the ultrasmall unicellular red alga *Cyanidioschyzon merolae* 10D. Nature. 2004;428:653–7.

9. Arabidopsis Genome Initiative. Analysis of the genome sequence of the flowering plant *Arabidopsis thaliana*. Nature. 2000;408:796–815.

10. Rensing SA, Lang D, Zimmer AD, Terry A, Salamov A, Shapiro H, et al. The *Physcomitrella* genome reveals evolutionary insights into the conquest of land by plants. Science. 2008;319:64–9.

11. Banks JA, Nishiyama T, Hasebe M, Bowman JL, Gribskov M, dePamphilis C, et al. The *Selaginella* genome identifies genetic changes associated with the evolution of vascular plants. Science. 2011;332:960–3.

12. Hori K, Maruyama F, Fujisawa T, Togashi T, Yamamoto N, Seo M, et al. *Klebsormidium flaccidum* genome reveals primary factors for plant terrestrial adaptation. Nat. Commun. 2014;5:3978.

13. Merchant SS, Prochnik SE, Vallon O, Harris EH, Karpowicz SJ, Witman GB, et al. The *Chlamydomonas* genome reveals the evolution of key animal and plant functions. Science. 2007;318:245–50.

14. Blanc G, Agarkova I, Grimwood J, Kuo A, Brueggeman A, Dunigan DD, et al. The genome of the polar eukaryotic microalga *Coccomyxa subellipsoidea* reveals traits of cold adaptation. Genome Biol. 2012;13:R39.

15. Blanc G, Duncan G, Agarkova I, Borodovsky M, Gurnon J, Kuo A, et al. The *Chlorella variabilis* NC64A genome reveals adaptation to photosymbiosis, coevolution with viruses, and cryptic sex. Plant Cell. 2010;22:2943–55.

16. Worden AZ, Lee J-H, Mock T, Rouzé P, Simmons MP, Aerts AL, et al. Green evolution and dynamic adaptations revealed by genomes of the marine picoeukaryotes *Micromonas*. Science. 2009;324:268–72.

17. Derelle E, Ferraz C, Rombauts S, Rouzé P, Worden AZ, Robbens S, et al. Genome analysis of the smallest free-living eukaryote *Ostreococcus tauri* unveils many unique features. Proc. Natl. Acad. Sci. U. S. A. 2006;103:11647–52.
