# Supplementary material for: Unexpected conservation of the RNA splicing apparatus in the highly streamlined genome of Galdieria sulphuraria
Source: BMC Evol Biol. 2018 Apr 2;18:41. doi: 10.1186/s12862-018-1161-x (PMC5880011; doi:10.1186/s12862-018-1161-x)
Supplement: Supplementary file 2 — Figure S1. Two examples of spliceosomal single-gene phylogeny that show different ancestries of red algal spliceosomal genes. (PDF 137 kb) [file 12862_2018_1161_MOESM2_ESM.pdf]

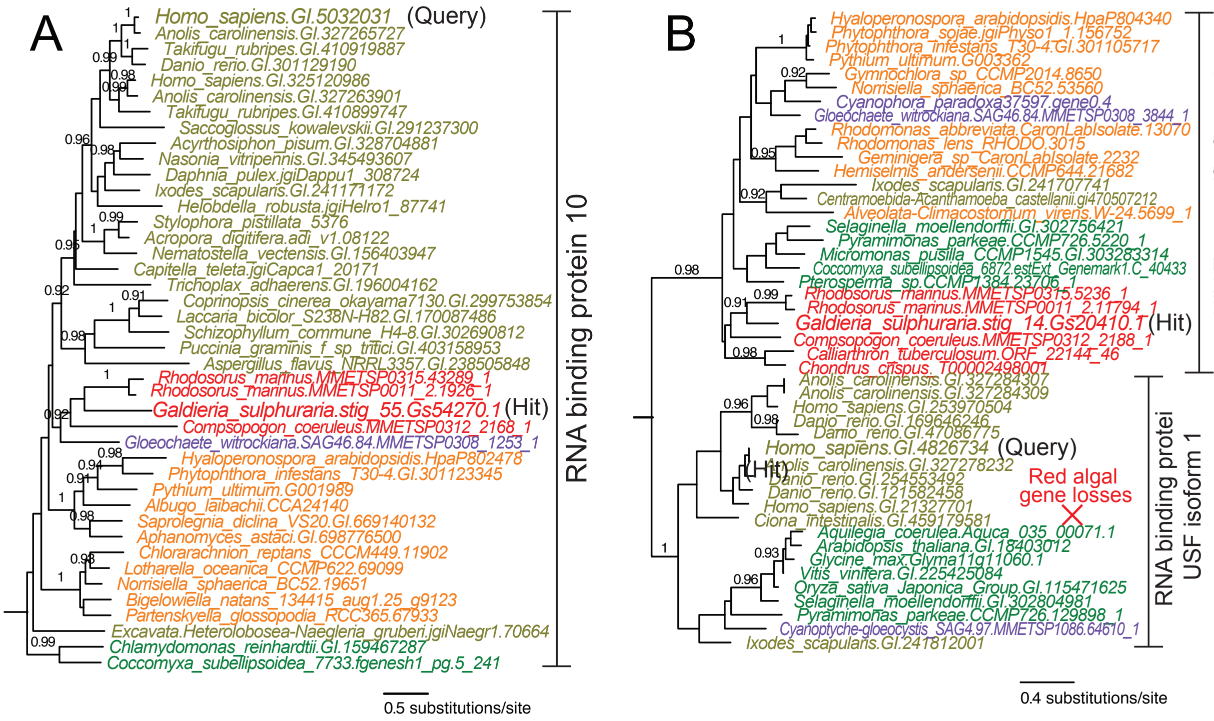

Figure S1. Two examples of spliceosomal single-gene phylogeny that show different ancestries of red algal spliceosomal genes (see Methods). (A) Maximum likelihood tree of RNA binding protein 10. Branch support values (when larger than 0.9) are shown and local support values were estimated using the Shimodaira-Hasegawa test (Shimodaira, H. and Hasegawa, M. 1999). In this tree, *Galdieria sulphuraria* gene (Gs54270.1) is one of the top six BLASTp hits to the human query gene (GI: 5032031). When included in the same phylogenetic tree, these two genes appeared in a same orthologous group. This result suggests a shared ancestry and an orthologous relationship between the *G. sulphuraria* and human genes. Species are shown in difference colors including red (red algae), green (Viridiplantae), glaucophytes (pink), orange (chromalveolates), and Metazoa and fungi (brown). (B) Maximum likelihood tree of RNA binding protein USF isoform 1 and HIV Tat-specific factor 1. The *G. sulphuraria* gene (Gs20410.1) is one of the top six BLASTp hits to the human query gene (GI: 4826734). When included in the same phylogenetic tree, the *G. sulphuraria* gene appeared in a separate orthologous group than the human query gene. All six *G. sulphuraria* top hits resulted in similar phylogenetic patterns. Given this result, the eukaryotic gene (RNA binding protein USF) was regarded to be lost in red algae (indicated by the cross in red color).
