# Supplementary material for: Unexpected conservation of the RNA splicing apparatus in the highly streamlined genome of Galdieria sulphuraria
Source: BMC Evol Biol. 2018 Apr 2;18:41. doi: 10.1186/s12862-018-1161-x (PMC5880011; doi:10.1186/s12862-018-1161-x)
Supplement: Supplementary file 4 — Table S2. Completeness of proteomic data estimated using 303 BUSCO gene families that are evolutionarily conserved among eukaryotes. (PDF 119 kb) [file 12862_2018_1161_MOESM4_ESM.pdf]

Table S2. Completeness of proteomic data estimated using 303 BUSCO gene families that are evolutionarily conserved in eukaryotes. The two taxa with significantly lower hits to BUSCO gene families are highlighted in the yellow field.

Protein data	Full-length	Proportion	Fragmented	Proportion	Missing	Proportion
C. merolae	283	93.4%	10	3.3%	10	3.3%
G. phlegrea	280	92.4%	6	2.0%	17	5.6%
G. sulphuraria	276	91.1%	7	2.3%	20	6.6%
P. purpureum+aerugineum	276	91.0%	9	3.0%	18	6.0%
R. marinus	274	90.4%	8	2.6%	21	7.0%
G. chorda	272	89.8%	7	2.3%	24	7.9%
C. crispus	**231**	**76.2%**	**23**	**7.6%**	**49**	**16.2%**
P. umbilicalis+P. yezoensis	**171**	**56.5%**	**75**	**24.8%**	**57**	**18.7%**
