# Supplementary material for: Unexpected conservation of the RNA splicing apparatus in the highly streamlined genome of Galdieria sulphuraria
Source: BMC Evol Biol. 2018 Apr 2;18:41. doi: 10.1186/s12862-018-1161-x (PMC5880011; doi:10.1186/s12862-018-1161-x)
Supplement: Supplementary file 5 — Table S3. Presence and absence of human spliceosomal machinery-associated proteins in red algae. (PDF 134 kb) [file 12862_2018_1161_MOESM5_ESM.pdf]

Table S3. Presence and absence of human spliceosomal machinery-associated proteins in red algae. The information regarding gene expression and protein categories was derived from the previous paper (Ref. 13). "+" indicates gene presence. Redundant genes (due to metazoan-specific duplication), when belong to the same categories, were collapsed. Human paralogous genes are shown in parentheses. Abbreviations: Gph (*G. phlegrea*), Gsu (*G. sulphuraria*), Rma (*R. marinus*), Gch (*G. chorda*), Ccr (*C. crispus*), Ppu (*Porphyridium*), Ban (Bangiacea), and Cme (*C. merolae*).

Human gene	Expression	Category	Gph	Gsu	Rma	Gch	Ccr	Por	Ban	Cme
SNRPB	high	**Sm**	+	+	+	+	+	+	+	+
SNRPD1	high	**Sm**	+	+	+	+	+	+	+	+
SNRPD2	high	**Sm**	+	+	+	+	+	+	+	+
SNRPD3	high	**Sm**	+	+	+	+	+	+	+	+
SNRPE	high	**Sm**	+	+	+	+	+	+	+	+
SNRPF	high	**Sm**	+	+	+	+	+	+		
SNRPG	high	**Sm**	+	+	+	+	+	+	+	+
SRPK1	high	**U1**	+	+	+	+	+	+	+	+
SNRNP70	high	**U1**	+	+	+	+	+		+	
SNRPA	high	**U1**	+	+	+	+	+			
SNRPC	high	**U1**	+	+	+	+	+	+	+	
PHF5A	high	**U2**	+	+	+	+	+	+	+	+
SF3A1	high	**U2**	+	+	+	+	+	+	+	
SF3A2	high	**U2**	+	+	+	+	+	+	+	+
SF3A3	high	**U2**	+	+	+	+	+	+	+	+
SF3B1	high	**U2**	+	+	+	+	+	+	+	+
SF3B14	high	**U2**	+	+	+	+	+	+	+	
SF3B2	high	**U2**	+	+	+	+	+	+	+	+
SF3B3	high	**U2**	+	+	+	+	+	+	+	+
SF3B4	high	**U2**	+	+	+	+	+	+	+	+
SF3B5	high	**U2**	+	+	+	+	+	+		
SNRPA1	high	**U2**	+	+	+	+	+	+	+	
SNRPB2(SNRPA)	high	**U2**	+	+	+	+	+			
PPIH	high	**U4/U6**	+	+	+	+	+			
NHP2L1	high	**U4/U6**	+	+	+	+	+	+	+	+
PRPF3	high	**U4/U6**	+	+	+	+	+	+		
PRPF31	high	**U4/U6**	+	+	+	+	+	+	+	+
PRPF4	high	**U4/U6**	+	+	+	+	+			
CD2BP2	high	**U5**	+	+	+					
SNRNP40	high	**U5**	+	+	+					
DDX23	high	**U5**	+	+	+	+	+	+	+	
EFTUD2	high	**U5**	+	+	+	+	+	+	+	+
PRPF6	high	**U5**	+	+	+	+	+	+		
PRPF8	high	**U5**	+	+	+	+	+	+	+	+
SNRNP200	high	**U5**	+	+	+	+	+	+	+	+
TXNL4A	high	**U5**	+	+	+	+	+	+	+	+
DHX16	high	**1st step**	+	+	+	+	+	+	+	
GPKOW	high	**1st step**	+	+		+	+			
DHX38	high	**2nd step**	+	+	+	+	+	+		
DHX8	high	**2nd step**	+	+	+	+	+	+	+	
PRPF18	high	**2nd step**	+	+	+	+	+	+	+	
SLU7	high	**2nd step**	+	+	+	+	+	+	+	
SNRNP27	high	U5Â°U4/U6								

SRPK1(2)	high	U5Â°U4/U6	+	+	+	+	+	+	+	+
SART1	high	U5Â°U4/U6	+		+	+	+	+		
USP39	high	U5Â°U4/U6	+	+	+	+	+	+	+	
LSM2	high	LSm	+	+	+	+	+	+		+
LSM3	high	LSm	+	+	+	+	+	+	+	+
LSM4	high	LSm	+	+	+	+	+	+	+	
LSM5	high	LSm	+	+		+		+		+
LSM6	high	LSm	+	+	+	+	+	+	+	+
LSM7	high	LSm	+	+	+	+	+	+	+	+
NAA38	high	LSm			+	+		+		
BUB3	high	A	+	+	+	+	+			
C19orf43	high	A								
CCAR1	high	A								
CDK11A	high	A	+	+	+	+	+			
FUS	high	A								
RBM10(5)	high	A	+	+	+					
RBM5	high	A	+	+	+					
SUGP1	high	A								
DDX5	high	A	+	+	+	+	+	+	+	+
HTATSF1	high	A	+	+	+	+	+	+		
PRPF40A	high	A	+	+	+	+	+	+		
RBM25	high	A								
SF1	high	A	+	+	+	+	+	+	+	+
TCERG1	high	A								
DDX17(5)	low	A	+	+	+	+	+	+	+	+
LUC7L	low	A	+	+	+	+	+	+	+	
RBM23/39	low	A	+	+	+	+	+	+		
HSPB1	high	B								
IK	high	B								
KIN	high	B	+	+	+	+	+	+	+	+
MFAP1	high	B	+	+	+	+	+		+	
PRPF4B	high	B	+	+	+			+		
SMU1	high	B	+	+	+					
THRAP3	high	B								
PRPF38A	high	B	+	+	+	+	+	+	+	+
UBL5	high	B	+	+	+	+	+	+	+	
ZMAT2	high	B	+	+	+	+	+	+	+	
CCDC12	high	B act	+	+	+	+	+			
FRG1	high	B act								
PPIL2	high	B act								
ZNF830	high	B act								
CDC40	high	B act	+	+	+	+	+	+	+	
CWC22	high	B act	+	+	+	+	+	+	+	
CWC27	high	B act	+	+	+	+	+	+		
RNF113A	high	B act	+	+	+	+	+	+	+	
SRRM2	high	B act	+	+						
C19orf29	high	C	+	+	+	+	+	+	+	
C1orf55	high	C	+	+	+	+	+	+		
C9orf78	high	C			+	+	+			
CXorf56	high	C								
DDX41	high	C	+	+	+	+		+	+	
DHX35	high	C	+	+						
FAM32A	high	C	+	+	+		+	+		

FAM50A/B	high	C	+	+	+	+	+	+	+	
GPATCH1	high	C	+	+	+					
NOSIP	high	C	+	+	+	+	+		+	
PPIG	high	C								
PPIL3	high	C	+	+	+	+	+	+	+	
PPWD1	high	C	+	+	+	+		+		
WDR83	high	C	+	+	+		+			
CCDC130	low	C2	+	+	+					
CDK10	low	C2	+	+	+	+				
CSDA(YBX1)	low	C2								
DGCR14	low	C2	+	+	+					
DHX57	low	C2				+	+			+
FRA10AC1	low	C2	+		+					
GNB2L1	low	C2	+	+	+	+	+	+	+	+
HSPA1A	low	C2	+	+	+	+	+	+	+	+
JUP	low	C2								
MATR3	low	C2								
NKAP	low	C2	+	+	+	+	+	+	+	
RBM4	low	C2								
TFIP11	low	C2	+	+	+	+	+	+		
TOE1	low	C2	+	+	+	+	+	+	+	+
TTC14	low	C2								
ZCCHC10	low	C2								
EIF4A3	high	EJC/TREX	+	+	+	+	+	+	+	+
MAGOH	high	EJC/TREX	+	+	+				+	
RBM8A	high	EJC/TREX	+	+	+					
SAP18	high	EJC/TREX								
ACIN1	low	EJC/TREX								
C17orf85	low	EJC/TREX				+	+	+		+
DDX39B	low	EJC/TREX	+	+	+	+	+	+	+	+
PNN	low	EJC/TREX	+	+	+					
RNPS1	low	EJC/TREX								
THOC1	low	EJC/TREX	+	+	+	+	+			
THOC2	low	EJC/TREX	+	+	+	+	+	+	+	+
THOC3	low	EJC/TREX	+	+	+	+	+			
THOC4	low	EJC/TREX	+	+	+	+	+	+	+	+
THOC5	low	EJC/TREX	+	+	+	+	+			
THOC7	low	EJC/TREX								
HNRNPA3/A0/A1/A2B1/A3/D	low	hnRNP	+	+	+	+	+	+	+	+
HNRNPC/RALY	low	hnRNP	+		+	+	+		+	+
HNRNPH2	low	hnRNP								
HNRNPH3/F/H1	low	hnRNP								
HNRNPK	low	hnRNP								
HNRNPM	low	hnRNP	+	+	+	+	+	+	+	+
HNRNPU/HNRNPUL1	low	hnRNP								
PCBP2/1	low	hnRNP								
PTBP2/1	low	hnRNP								
RBMX	low	hnRNP	+	+						
SYNCRIP/H	low	hnRNP								

NRNPR										
AGGF1	low	MISC								
BAG2	low	MISC								
C16orf80	low	MISC								
CCDC55	low	MISC								
CELF1	low	MISC	+	+	+			+		
CIRBP(RBMX)	low	MISC	+	+						
CLNS1A	low	MISC								
DHX9	low	MISC								
DNAJC6(SRSF3)	low	MISC	+	+	+	+	+	+	+	+
GCFC1	low	MISC	+	+						
ILF3	low	MISC								
KHDRBS3/1	low	MISC	+	+	+	+	+	+	+	
KIAA1967(CCAR1)	low	MISC								
NRIP2	low	MISC								
PPIL4	low	MISC	+	+	+	+	+			
PPP1CA	low	MISC	+	+	+	+	+	+	+	+
PPP1R8	low	MISC								
PRMT5	low	MISC	+	+	+	+	+	+	+	
QKI	low	MISC	+	+	+	+	+	+	+	+
RBBP6	low	MISC	+	+	+	+	+	+	+	+
RBFOX2	low	MISC								
RBM42	low	MISC	+	+	+	+	+	+	+	
SAP30BP	low	MISC	+	+	+	+				
SEC31B	low	MISC	+	+	+	+	+	+	+	+
SMN1(D3)	low	MISC								
WDR77	low	MISC								
ZNF207	low	MISC	+		+	+	+	+		
NCBP1	high	mRNA	+	+	+	+	+	+	+	+
NCBP2	high	mRNA	+	+	+	+	+	+	+	+
DDX3X	low	mRNA	+	+	+	+	+	+	+	+
ELAVL1	low	mRNA								
ILF2(3)	low	mRNA								
PABPC1	low	mRNA	+	+	+	+	+	+	+	+
RBM7	low	mRNA								
SRRT	low	mRNA	+	+	+	x	+	+	+	
YBX1	low	mRNA								
ZC3H18	low	mRNA								
CTNNBL1	high	Prp19	+	+	+		+			
PQBP1	high	Prp19								
WBP11	high	Prp19								
BCAS2	high	Prp19	+	+	+			+		
CDC5L	high	Prp19	+	+	+	+	+		+	
CWC15	high	Prp19	+	+	+	+	+	+		
HSPA8	high	Prp19	+	+	+	+	+	+	+	+
PLRG1	high	Prp19	+	+	+	+	+	+	+	+
PRPF19	high	Prp19	+	+	+	+	+			
PPIE	high	Prp19 rel								
PPIL1	high	Prp19 rel	+	+	+			+		
PRCC	high	Prp19 rel								
AQR	high	Prp19 rel	+	+	+		+	+		

BUD31	high	Prp19 rel								
CRNKL1	high	Prp19 rel	+	+	+	+	+	+	+	
ISY1	high	Prp19 rel	+	+	+	+	+	+	+	
RBM22	high	Prp19 rel	+	+	+	+	+	+	+	
SNW1	high	Prp19 rel	+	+	+	+	+	+		
SYF2	high	Prp19 rel	+	+	+	+	+	+		
XAB2	high	Prp19 rel	+	+	+	+	+	+		
BUD13	high	RES	+	+		+	+	+		
RBMX2	high	RES	+	+	+	+	+	+	+	
SNIP1	high	RES	+	+	+					
ARGLU1	low	SR								
SRRM1	low	SR	+	+	+					
SRSF1/9	low	SR	+	+	+				+	
SRSF10	low	SR							+	
SRSF2	low	SR	+	+	+				+	
SRSF3	low	SR	+	+	+	+	+	+	+	+
SRSF6/4/5	low	SR	+	+	+		+		+	
SRSF7	low	SR	+	+	+	+	+	+	+	+
TRA2B/2A	low	SR								
CHERP	high	U2 rel								
DDX42	high	U2 rel	+	+	+		+	+	+	
DNAJC8	high	U2 rel								
PUF60	high	U2 rel								
RBM17	high	U2 rel								
SMNDC1	high	U2 rel								
U2AF1	high	U2 rel	+	+	+	+	+	+	+	
U2SURP	high	U2 rel	+	+	+					
DDX46	high	U2 rel	+	+	+	+	+	+	+	+
DHX15	high	U2 rel	+	+	+	+	+	+	+	+
U2AF2	high	U2 rel	+	+	+	+	+	+	+	
