# Supplementary material for: Unexpected conservation of the RNA splicing apparatus in the highly streamlined genome of Galdieria sulphuraria
Source: BMC Evol Biol. 2018 Apr 2;18:41. doi: 10.1186/s12862-018-1161-x (PMC5880011; doi:10.1186/s12862-018-1161-x)
Supplement: Supplementary file 6 — Figure S2. The search results for snRNA component of the spliceosome in Galdieria sulphuraria. (PDF 91 kb) [file 12862_2018_1161_MOESM6_ESM.pdf]

Figure S2. The search results for snRNA component of the spliceosome in *Galdieria sulphuraria*. The outputs are from INFERNAL v1.1.2 under the default setting. Only alignments for the top hit for each query RNA are shown.

```
Query:       U1  [CLEN=166]
Accession:   RF00003
Description: U1 spliceosomal RNA

Hit scores:
 rank     E-value  score  bias  sequence  start    end   mdl trunc   gc  description
 ----   --------- ------ -----  -------- ------ ------   --- ----- ----  -----------
  (1) !   1.8e-10   47.1   0.0  stig_36  123158 122976 -  cm    no 0.51  -


Hit alignments:
>> stig_36
 rank     E-value  score  bias mdl mdl from   mdl to       seq from      seq to       acc trunc   gc
 ----   --------- ------ ----- --- -------- --------    ----------- -----------      ---- ----- ----
  (1) !   1.8e-10   47.1   0.0 cm         1      166 []      123158      122976 - .. 0.76    no 0.51

                                v                                v                                                NC
                 :::::::::::((((<<<<<<-<<<<<__________.>>>>>>>>>>>,<<<------<<<-~~~~~~->>->-----.>>>,,<<<-<<-<<__ CS
       U1      1 AUACUUACCUGGccggGGggaaacggcGAUCAaGAAG.gccguucccCCcagGgugaGGucccu*[15]*gggugcugac.cCcuGcGgUucccCCCa 103
                 AUACUUACCUGG::G :::G:  :::C AUC   A   G::: :C:::  :GG GA G C::       G::UG+UGA+ CC:U CG: ::C:C A
  stig_36 123158 AUACUUACCUGGGAGGGCAGUCUGUAC-AUCGCAAGAcGUACGACUGUG-CGGGGACGACAA-*[34]*GUUUGUUGAAaCCGU-CGAGGUCACGA
123039
                 **************966666666555.55555444435555566665.777455444444....8..344444444448888.******88877 PP

                                                     v             v        NC
                 ____>>->>--->>>,))))--------------.-<<<<<--<<<<<____>>>>>->>>>>: CS
       U1    104 AAGUGGugaaAccCgAcggCAUAAUUUgUGgUAG.ucgGGGgaccGcgUUcGcgCggcCCCcgc 166
                    UG:+::   :CGAC::C  AAUUU UG       ::G :G  :GCGUUCGCGC: C: C::C
  stig_36 123038 CUAUGUGGC-UGUCGACUCCUCAAUUUAUGACCCuGGAGUAGUAUGCGUUCGCGCAGCUGCUCC 122976
                 777888777.9*****************9754249**************************** PP
```

```
Query:       U2  [CLEN=193]
Accession:   RF00004
Description: U2 spliceosomal RNA

Hit scores:
 rank     E-value  score  bias  sequence  start    end   mdl trunc   gc  description
 ----   --------- ------ -----  -------- ------ ------   --- ----- ----  -----------
  (1) !   1.7e-27  130.3   0.0  stig_27    8229   8421 +  cm    no 0.46  -
 ------ inclusion threshold ------
  (2) ?       1.2   18.4   0.0  stig_13  117529 117438 -  cm    no 0.36  -
  (3) ?       1.7   17.8   0.7  stig_26   77195  77345 +  cm    no 0.34  -


Hit alignments:
>> stig_27
 rank     E-value  score  bias mdl mdl from   mdl to       seq from      seq to       acc trunc   gc
 ----   --------- ------ ----- --- -------- --------    ----------- -----------      ---- ----- ----
  (1) !   1.7e-27  130.3   0.0  cm        1      193 []        8229        8421 + .. 0.95    no 0.46

                                                                                      v  v   v  v                   NC
               ::::::<<<.-<<<<____>>>>->>>,,,,,,,,,,,,,,,,,,,<<<<<<________>>>>>>,<<<<<<<___>>>>>>>,,,,,,,,,,,,,, CS
       U2    1 AUacCUUCu.cgGCcUUUUgGCuaaGAUCAAGUGUAGUAUCUGUUCUUauCAGUuUAAuAuCUGauAuggccccAuuggggccaauuauaUUAaauua 99
                 A CUUCU  :G CU UUG C: AGAUCAAGUGUAGUAUCUGUUCU::::AGU+UAA+A+CU::::UG : :C A U G: : C    +U UUA A  +
  stig_27 8229 UGAUCUUCUcGAGUCUAUUGGCUCAGAUCAAGUGUAGUAUCUGUUCUAUCAAGUGUAACAACUUGAUUGGGGUCCACUUGGGCUCCC-GUUGUUACAAGU
8327
               ********668****************************************************9999888765666666666.799********* PP

                                   vv                    vv                                                NC
               ,,,,,,,,,,,,,<<<<<<<<----.<<<<<__>>>>>-->>>>>>>>,,<<<<<<-<<<<<<___________>>>>>>-->>>>>>:::::: CS
       U2  100 AUUUUUggaacuaGugggggcauuu.uggGCUUGCccauugcccccaCacgguugaccuggcaUUGCAcUaccgccagguucagcccAcccuuu 193
               AUUUUU GA ++ G: ::G  ++ U :::GCUUGC:::++  C:: :CA G::UUG CCU:G: UUGCACU C :C:AGG+ CAG::CACC++UU
  stig_27 8328 AUUUUUCGAGAGGGGGUAGGUUAGUuACUGCUUGCAGUAACACUAUCCAAGCUUUGCCCUAGUCUUGCACUGC-ACUAGGAGCAGAGCACCUCUU 8421
               ****************999888888****************************************.******************** PP
```

```
Query:       U4  [CLEN=140]
Accession:   RF00015
Description: U4 spliceosomal RNA

Hit scores:
 rank     E-value  score  bias  sequence  start    end   mdl trunc   gc  description
 ----   --------- ------ ----- --------- ------ ------   --- ----- ----  -----------
  (1) !   4.2e-20   95.0   0.0  stig_26   82879  82687 -  cm    no 0.42  -


Hit alignments:
>> stig_26
 rank     E-value  score  bias mdl mdl from   mdl to       seq from      seq to       acc trunc   gc
 ----   --------- ------ ----- --- -------- --------    ----------- -----------      ---- ----- ----
  (1) !   4.2e-20   95.0   0.0  cm        1      140 []       82879       82687 - .. 0.98    no 0.42

                                       v                          v                                        v      v NC
                ::::::::::::::::<<-<<<<<-<<<_________________>>>>>>>>-->>,,,,<<<<____________>>>>,,,,<<<<<<<<<<<<< CS
       U4     1 AUCcUUGuGCAgGGGcAaUaccccugcCAGUGAGGaUucguCcGAGgcggggUauUGCUAgUugAAAACUAuuucccaAcaccCgccucauggccuc 98
                 UC+UUG+GCA GGGCAAU::C C :C:AGUGAGG+UU+ +CCGA:G:G G::UAUUGCUA:UUGAAAACUA U C CAA:+CC GC :: U::CCU
  stig_26 82879 GUCUUUGCGCACGGGCAAUGUCUC-CCAAGUGAGGUUUU-ACCGAUGGGCGACUAUUGCUAAUUGAAAACUACU-C-CAAUUCCAGCGCGUUCACCUC
82786
                ************************.**************.*********************************9.6.********************* PP

                    v      v                                                                                        NC
                ____>>>>>>>>>>->>>,,,,,,,,,,,,<<<<__...........................................................__> CS
       U4    99 uucagaggccaugaaggcAAUUUUUGgAAuccccuu...........................................................aag 137
                 UC  AGG::A ::  GCAAUUUUUG+AA C::: U                                                           A+:
  stig_26 82785 -UCGUAGGUGACCG-UGCAAUUUUUGAAACCUUUCUccuuuugauaauaaacgcuuuuuuaauuaaacaguucccugguuguaaacugccaauagAUA
82690
                .*************.********************************************************************************99* PP

                    NC
                >>> CS
       U4   138 ggg 140
                ::G
  stig_26 82689 AAG 82687
                *** PP
```

```
Query:       U5  [CLEN=116]
Accession:   RF00020
Description: U5 spliceosomal RNA

Hit scores:
 rank     E-value  score  bias  sequence  start    end   mdl trunc   gc  description
 ----   --------- ------ -----  -------- ------ ------   --- ----- ----  -----------
  (1) !   3.9e-08   58.8   0.0  stig_15  160149 160033 -  cm    no 0.44  -


Hit alignments:
>> stig_15
 rank     E-value  score  bias mdl mdl from   mdl to       seq from      seq to       acc trunc   gc
 ----   --------- ------ ----- --- -------- --------    ----------- -----------      ---- ----- ----
  (1) !   3.9e-08   58.8   0.0  cm        1      116 []      160149      160033 - .. 0.95    no 0.44

                               vv                                              vv                                 NC
                 ::<<<<--<<<<<<<<<<<.--------<<<<<<<<___________>>>>>>>>--->>>>>>>>>>>------..>>>>--------.------ CS
       U5      1 auccccuGgggccacuaca.uauCGAAcaagucUcUcGCCUUUUACuAgAgacuuCCGuguaguggcccaauuaa..gggguuuaaacu.AAUUUU 92
                 + ::C: G:G :C:  ::: ++U G AC::::CU:UCGC UUUUACUA:AG::::CC :::  :G: C:+AU+ +  :G::+UU+AAC+ AAUUUU
  stig_15 160149 UGAGCUGGCGGUCUCUGUCaAUUUGUACUCUCCUUUCGCUUUUUACUAAAGGAGACCAGGCUUAGGUCGCAUAGUacAGCUCUUUAACCuAAUUUU
160054
                 *******************77778**************************************************************9998999999 PP

                    v                   v NC
                 ---<-<<<<<<______>>>>>>> CS
       U5     93 UGgaagcccuuuuuuuuaagggcu 116
                 UG   :::CU: ++  +:AG:::U
  stig_15 160053 UGU--CGUCUCCCAA-AGAGACGU 160033
                 998..9999997665.6999999* PP
```

```
Query:       U6  [CLEN=104]
Accession:   RF00026
Description: U6 spliceosomal RNA

Hit scores:
 rank     E-value  score  bias  sequence  start    end   mdl trunc   gc  description
 ----   --------- ------ -----  -------- ------ ------   --- ----- ----  -----------
  (1) !   1.3e-23   96.2   0.0  stig_48   62703  62604 -  cm    no 0.45  -

Hit alignments:
>> stig_48
 rank     E-value  score  bias mdl mdl from   mdl to       seq from      seq to       acc trunc   gc
 ----   --------- ------ ----- --- -------- --------    ----------- -----------      ---- ----- ----
  (1) !   1.3e-23   96.2   0.0  cm        1      104 []       62703       62604 - .. 0.99    no 0.45

                                                                                                        NC
               <<<<<______>>>>>:::::::::::::::::::::::::::::::::::::::::::::::::::::::::::::::::: CS
       U6     1 guccccUUCGggggacaUaUggUaAAaUUGgAACgAUACAGAGAAGAUUAGCAUGGCCCCUGCaCAAGGAUGACACguauaauagAGAgauguaccau 98
               GUCC  UUCG  GGACAUA G  AAAAUUGGAA +AUACAGAGAAGAUUAGCAUGGCCCCUGC+CAAGGAUGACACG+A+AA +  GAGA+G +CC +
  stig_48 62703 GUCC--UUCG--GGACAUACGCAAAAAUUGGAAAAAUACAGAGAAGAUUAGCAUGGCCCCUGCGCAAGGAUGACACGCAAAACUUCGAGAAGAUCCCA
62610
               ***8..8888..8************************************************************************************* PP

                      NC
               :::::: CS
       U6    99 aUUUUU 104
                UUUUU
  stig_48 62609 GUUUUU 62604
               ****** PP
```
