# Supplementary material for: Unexpected conservation of the RNA splicing apparatus in the highly streamlined genome of Galdieria sulphuraria
Source: BMC Evol Biol. 2018 Apr 2;18:41. doi: 10.1186/s12862-018-1161-x (PMC5880011; doi:10.1186/s12862-018-1161-x)
Supplement: Supplementary file 7 — Table S4. INFERNAL scores and e-values for red algal snRNA genes. (PDF 64 kb) [file 12862_2018_1161_MOESM7_ESM.pdf]

Table S4. INFERNAL scores and e-values for red algal snRNA genes. Only statics from the top hits for each taxon are shown. High sequence conservation (high scores and low e-values) is shown in red color and low sequence conservation in blue color.

Scores

Taxa	U1	U2	U4	U5	U6
Galdieria sulphuraria	47.1	130.3	95	58.8	96.2
Pyropia yezoensis	23	103.6	59.1	59.2	42.9
Chondrus crispus	48.4	109.9	38.4	41	55.5
Gracilariopsis chorda	64.2	101.3	38.7	29.8	47.7
Porphyridium purpureum	22.5	82.5	58.3	32.6	22.6
Cyanidioschyzon merolae	NA	23.6	27.8	26.1	19.6

E-value

Taxa	U1	U2	U4	U5	U6
Galdieria sulphuraria	1.80E-10	1.70E-27	4.20E-20	3.90E-08	1.30E-23
Pyropia yezoensis	9.10E-03	1.40E-20	1.60E-10	1.00E-07	1.20E-07
Chondrus crispus	5.60E-10	1.10E-21	6.20E-05	2.40E-03	6.80E-11
Gracilariopsis chorda	1.00E-14	1.10E-19	5.00E-05	6.70E-02	1.10E-08
Porphyridium purpureum	5.60E-03	7.20E-16	1.10E-10	3.20E-02	4.20E-02
Cyanidioschyzon merolae	NA	8.00E-02	4.80E-03	7.30E-01	2.80E-01
