# Supplementary material for: Unexpected conservation of the RNA splicing apparatus in the highly streamlined genome of Galdieria sulphuraria
Source: BMC Evol Biol. 2018 Apr 2;18:41. doi: 10.1186/s12862-018-1161-x (PMC5880011; doi:10.1186/s12862-018-1161-x)
Supplement: Supplementary file 8 — Figure S3. Sequence conservation in Galdieria sulphuraria genes. (PDF 181 kb) [file 12862_2018_1161_MOESM8_ESM.pdf]

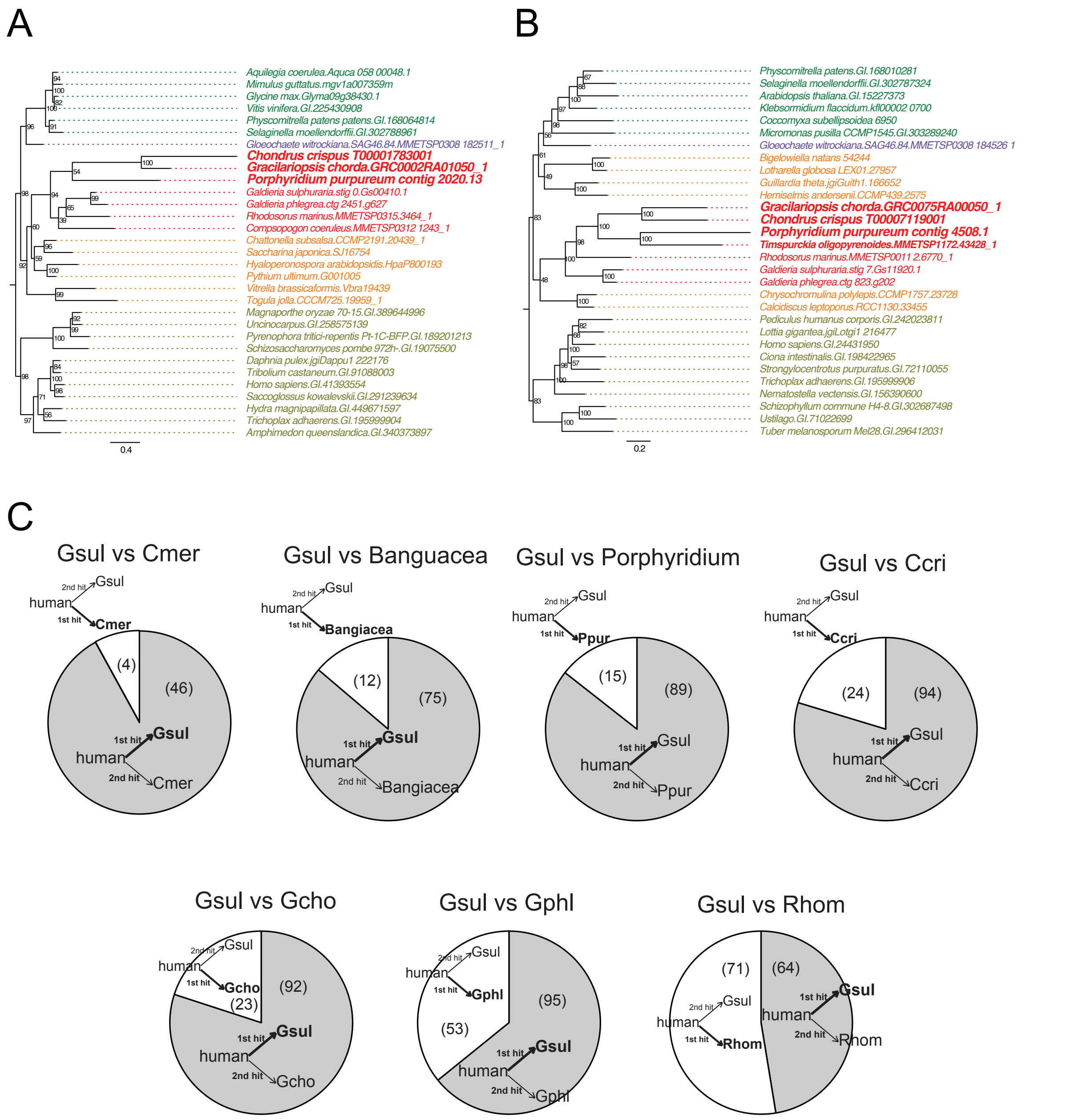

Figure S3. Sequence conservation in *Galdieria sulphuraria* genes. (A) Maximum likelihood tree of LUC7-like protein. Red algal taxa connected with exceedingly long branched are shown in bigger font and bold face. Node supports values are calculated using the ultrafast bootstrap (UFboot) approximation approach with 1500 bootstrap replicates. (B) Maximum likelihood tree of PRPF4 protein. (C) Pairwise compassion in sequence conservation using BLASTp bit-score as measure. Protein databases are constructed for proteins found in both red algal species that are compared (e.g., *G. sulphuraria* and *C. merolae*). The distribution of BLASTp top hits among the two red algal species is shown in pie charts. Abbreviation: Gsul (*Galdieria sulphuraria*), Cmer (*Cyanidiochyzon merolae*), Ccri (*Chondrus crispus*), Gcho (*Gracilariopsis chorda*), Gphl (*Galdieria phlegrea*), Rhom (*Rhodosorus marinus*).
