# Supplementary material for: Unexpected conservation of the RNA splicing apparatus in the highly streamlined genome of Galdieria sulphuraria
Source: BMC Evol Biol. 2018 Apr 2;18:41. doi: 10.1186/s12862-018-1161-x (PMC5880011; doi:10.1186/s12862-018-1161-x)
Supplement: Supplementary file 9 — Table S5. The intron statistics in red algal and Viridiplantae genomes. (PDF 71 kb) [file 12862_2018_1161_MOESM9_ESM.pdf]

Table S5. The intron statistics in red algal and viridiplantae genomes. Only introns flanked by coding exons are included. Coding DNA sequences (CDSs) with in-frame stop codon, ambiguous nucleotide or incomplete codon (length is not divisible by 3) were removed. Frequencies of the two most prevalent splice sites found in most species (GT-AG and GC-AG) are shown for each species.

Species	Species	CDS with intron(s)*	Intron number	GT-AG	GC-AG
Rhodophyta	*G. sulphuraria*	4655	13,245	98.5%	1.4%
Rhodophyta	*C. merolae*	26	27	96.3%	3.7%
Rhodophyta	*P. purpureum*	201	245	94.7%	/
Rhodophyta	*G. chorda*	1815	2249	96.1%	/
Rhodophyta	*C. crisps*	1602	2926	95.8%	2.6%
Rhodophyta	*P. yezeonsis*	2520	3500	95.4%	0.05%
Viridiplantae	*C. reinhardtii*	11386	93749	98.5%	0.4%
Viridiplantae	*K. flaccidum*	16306	94296	98.4%	1.3%
Viridiplantae	*P. patens*	27765	143729	97.6%	2.2%
Viridiplantae	*S. moellendorffii*	29017	161879	99.3%	0.3%
Viridiplantae	*A. thaliana*	40106	236649	98.6%	1.1%

*includes multiple splice variants derived from single gene locus for Viridiplantae.
