# Supplementary material for: Unexpected conservation of the RNA splicing apparatus in the highly streamlined genome of Galdieria sulphuraria
Source: BMC Evol Biol. 2018 Apr 2;18:41. doi: 10.1186/s12862-018-1161-x (PMC5880011; doi:10.1186/s12862-018-1161-x)
Supplement: Supplementary file 10 — Figure S4. GC content and intron density in red algae. (PDF 92 kb) [file 12862_2018_1161_MOESM10_ESM.pdf]

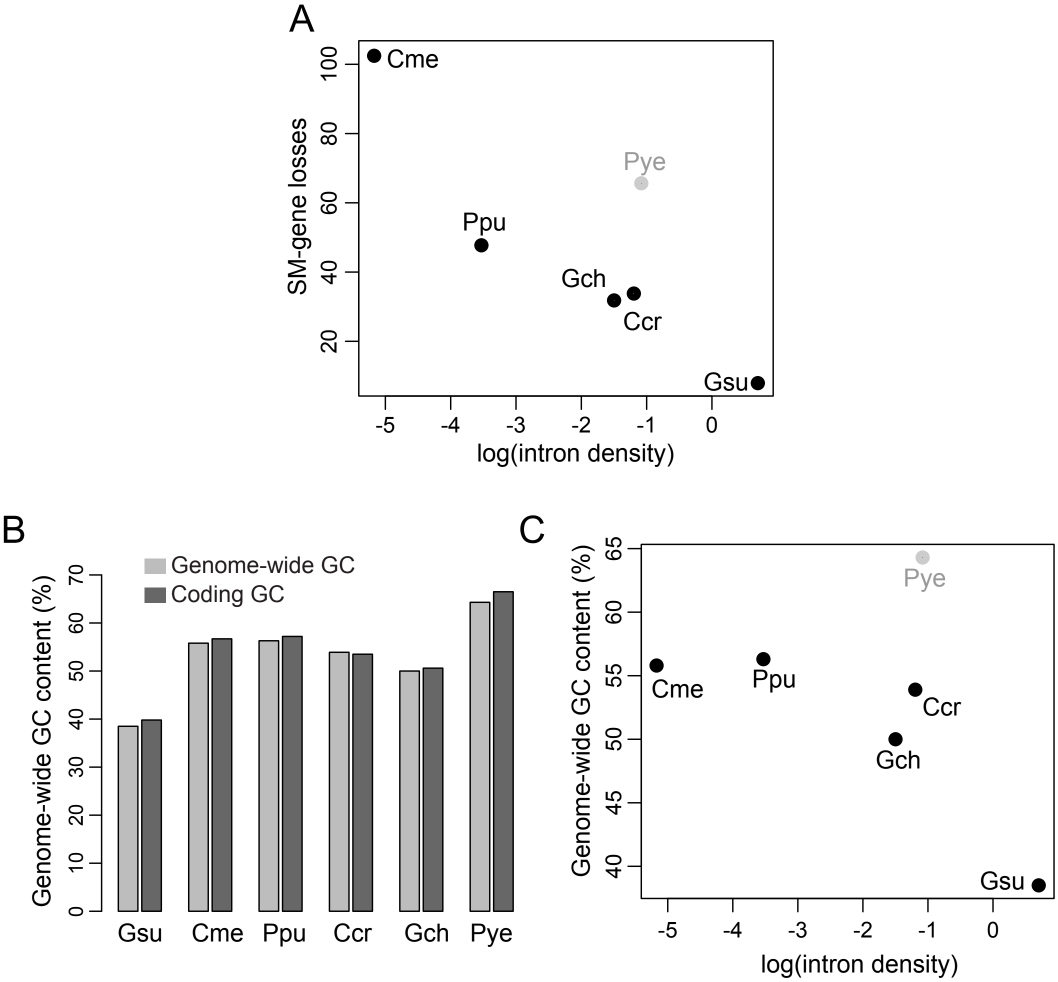

Figure S4. GC content and intron density in red algae. Only species with genome assemblies are included. (A) An obvious correlation between SM-gene losses and intron density. *Pyropia yezeonsis* (Pye, shown in grey color) has poor data completeness (see Additional file 4: Table S2) and likely has an inflated number of SM-gene losses. The correlation is apparent for the remaining five species. (B) GC contents in six red algal genome assemblies. (C) Scatter plot of GC content against intron density among the six red algal species. Abbreviation: Gsu (*Galdieria sulphuraria*), Cme (*Cyanidiochyzon merolae*), Ppu (*Porphyridium purpureum*) Ccr (*Chondrus crispus*), Gch (*Gracilariopsis chorda*), and Pye (*Pyropia yezeonsis*).
