# Supplementary material for: Unexpected conservation of the RNA splicing apparatus in the highly streamlined genome of Galdieria sulphuraria
Source: BMC Evol Biol. 2018 Apr 2;18:41. doi: 10.1186/s12862-018-1161-x (PMC5880011; doi:10.1186/s12862-018-1161-x)
Supplement: Supplementary file 11 — Figure S5. Conservation of intron positions in the Galdieria sulphuraria geranylgeranyl transferase beta-subunit gene. (PDF 97 kb) [file 12862_2018_1161_MOESM11_ESM.pdf]

Figure S5. Conservation of intron positions in *Galdieria sulphuraria* geranylgeranyl transferase beta-subunit gene. The residues flanking intron (phase 1) or interrupted by intron (phase 2 and 3) are highlighted in green color. Red algal taxa and Viridiplantae are shown in red and black text, respectively. The alignment was generated using MAFFT v7 with progressive methods (G-INS-1) (http://mafft.cbrc.jp/alignment/server/). Because the alignment of intron #1-containing region is highly variable depending on used methods and settings, this intron is not included in main text. Introns specific to Viridiplantae lineages are not shown. Abbreviations: Gsu (*Galdieria sulphuraria*), Gch (*Gracilariopsis chorda*), Ccr (*Chondrus crispus*), Pye (*Pyropia yezoensis*), Ppu (*Porphyridium purpureum*), Kfl (*Klebsormidium flaccidum*), Ppa (*Physcomitrella patens*), Smo (*Selaginella moellendorffii*), Ath (*Arabidopsis thaliana*), Cre (*Chlamydomonas reinhardtii*).

```
Gsu XP_00570604 M-------------------------------DRFTPLPVDLHQQYLCKA---QNDTKSW
Gch GRC0126RA00 MASS------------------------------REQLRIDKHVAYIVGL---EKKTKTT
Ccr XP_00571301 M-----------------------------------------------------------
Pye g4238       MGDG--------------------------EEEVPTILLRDKHIEYILSL---QDKQDSP
Ppu contig_3423 MMTS------------------DNKSHRSMDESVCAQLLAELHVESLLRL---EASSESY
Cre XP_00168980 MKAA------------------GTGPAEAGP---RSTLLVDKHATYIKSFSRLWDTPDKL
Kfl GAQ90956.1  MATQADDGMTSRAEAAAEGTECGNAGSEAGP-SGRSTLLVDKHKSYIKSL---DNDTATL
Ppa XP_00177348 ----------------------------------MGELYLDKHVAYIKSL----DKKKDF
Smo XP_00296800 ----------------------------------M-ELRVDKHVEFIKSL----EKKKDF
Ath NP_974770.1 MSSTSS--------------------------SQMVQLVADKHVRYI--LM-AEKKKESF

Gsu XP_00570604 EYF-LTAHKRISGAYWTLTSLDLLHALETT-DENAIISWIFSC-QHES-G----------
Gch GRC0126RA00 DALYFTDHKRLSGVYWALSSLALMRKLHVL-PQDTILDYVLSC-YHSDVA----------
Ccr XP_00571301 -----------------------------------------TC-YNPTIG----------
Pye g4238       EML-FADHRRLSGVYWAVSALDLLGARDRL-EKEAILDYVMSCYREEE-G----------
Ppu contig_3423 LQF-LLEHKKMSAMYWALTSLALLRSLHTLADRGAVLAFIKGC-ERET-TSAGEDKTNRS
Cre XP_00168980 EFV-ATEHFWMSGMYWGLSAMYLMGRLGDM-DRDAILGWVMRC-QHTN-G----------
Kfl GAQ90956.1  DYQ-LTEHLRMSGVYWGLTAMDLMHCLHEM-DSERVVKWVMSC-QHKN-G----------
Ppa XP_00177348 DAV-VMEHLRMSGAYWGLTALDIMGRLGDM-NVDEIVSWILMC-QDDC-G----------
Smo XP_00296800 DSM-VMEHLRMNGAYWGLTALDLMGCREDV-DDAKVISWLLQC-KHDC-G----------
Ath NP_974770.1 ESV-VMDHLRMNGAYWGLTTLDLLDKLGCV-SEEEVISWLMTC-QHES-G----------
                                                                 *

Gsu XP_00570604 ------GFGGNVDQDPNLLSTLSAVQCLALYRRLDELD--SEKVISYIASLQLSDGSFMG
Gch GRC0126RA00 ------AFSGNTDQDPHLLYTLSGVQILAMYDKLHLID--ADRVVANIAALQRPDGSFAG
Ccr XP_00571301 ------AFAGNTDQDAHLLYTLSGIQILAIYDNLSLID--VDKVVSYIRGLQKPDGSFAG
Pye g4238       ------GFGGHVDQDAHILYTLSALQVLVMYDALDRVD--VDAVSRFVGSLQQPDGSFIG
Ppu contig_3423 QIAPALGYAGNVDQDAHLLYTLSAVQCLAVLGEFVLIEAQADRIAAYVRSLQNADGSFCG
Cre XP_00168980 ------GFGGSERNDPHLLYTLSAVQILALYDRLDDVD--ADKVAAYVAGLQRPDGSFAG
Kfl GAQ90956.1  ------AFGGNRDHDAHLLYTLSAVQILALYDKLDLLD--GKKIAAYVVSLQQEDGSFAG
Ppa XP_00177348 ------GFGGNHEHDPHILYTLSAVQILALFDRVDAVD--ADKIASYISGLQNEDGSFSG
Smo XP_00296800 ------GFSGNIGHDPHILYTLSAVQILAIYDRMELLD--SDKAASYIASLQQEDGSFAG
Ath NP_974770.1 ------GFAGNTGHDPHILYTLSAVQILALFDKINILD--IGKVSSYVAKLQNEDGSFSG
                      .:.*   :*.::* ***.:* *.:   .  ::         :  **  **** *

Gsu XP_00570604 DVWG-EVDSRFTYAAILCLSILKRLDVIRVDKAVEFVISCLNFDGGFGCIPGAESHSGQV
Gch GRC0126RA00 DKWG-EIDTRFTYCALLCAAILRRSHLLDVPAAVRYVLRCQNFDGGFGCIAGAESHAGQV
Ccr XP_00571301 DEWG-EIDTRFSYCALLSLSILNRLDAVDVDAAVAFVLSCQNFDGGFGCIPGAESHAGQV
Pye g4238       SPTSPEVDTRFSYCALLSVTLLNRRSVLDLSAAGAFLASCTNFDGGYGCIPDAESHAGQI
Ppu contig_3423 DAYG-EVDTRFSYAALLTLQLIRRTDAVDVEAAVNYVLSCRNFDGGFGCIPFAESHAGQV
Cre XP_00168980 DAWG-EIDTRFTYCALLCLSILGRTAAINVPSALDFIAKCKNFDGGFGCTPGNESHAGQV
Kfl GAQ90956.1  DEFG-EIDTRFSYAAILCLSLLNKLHSINVPRAVDYIMQCQNFDGGFGVAPGLESHAGQI
```

```
Ppa XP_00177348 DEWG-EIDTRFSYCAICCLSLLKRLDKINLEKACNFVASCKNFDGGFGCAPGGESHAGQT
Smo XP_00296800 DEWG-EIDTRFSYCALCCLSLLKRLDVINVEKAVDYIASCKNFDGGFGSIPGGESHAGQI
Ath NP_974770.1 DMWG-EIDTRFSYIAICCLSILKCLDKINVEKAVKYIVSCKNLDGGFGCTPGAESHAGQI
                .  . *:*:**:* *:    ::      : :  *  ::  * *:***:*  .  ***:**

Gsu XP_00570604 FCCIGALYLTDS---LYRIDQELTGWWLAERQLKNGGLNGRPDKKADVCYSWWVLSSLAM
Gch GRC0126RA00 FCCVGSLYLADAMDEMTREDRERLALWLCERQLPCGGLNGRPDKLEDVCYSWWVLSSLKM
Ccr XP_00571301 FCCVGALALADA---LRRIDAELLGWWLCERQLPCGGFNGRPDKLEDVCYSWWVLASLAM
Pye g4238       FCVLAALALLGT---PTPPAAAQLCEWLASRQLPCGGLNGRPDKKEDVCYSWWVVASLSM
Ppu contig_3423 FCCVGALKLAGA---LDRVDQTTLGWWLCQRQLPNGGLNGRPDKKEDVCYSWWVLSSLSM
Cre XP_00168980 FTCIGALSLANA---LHLVDRDLFCWWLCERQTKSGGLNGRPEKLQDVCYSWWCLSCLSI
Kfl GAQ90956.1  FCCVASLALTGS---LHRLDRDLLGWWLAERQVKEGGLNGRPEKLPDVCYSWWVLSSLVI
Ppa XP_00177348 FCCVGALAIGGA---LHHVDKDLLGWWLAERQVKSGGLNGRPEKLPDVCYSWWVLASLVI
Smo XP_00296800 FCCVSALAIAGA---LHHIDKDLLGWWLCERQVKSGGLNGRPEKQPDVCYSWWVLSSLVT
Ath NP_974770.1 FCCVGALAITGS---LHHVDKDSLGWWLCERQLKAGGLNGRPEKLADVCYSWWVLSSLIM
                *  :.:* : .:              **..**   **:****:*  *******  ::.*

Gsu XP_00570604 LNKLDWIDSSKLIEFILHCQDLENGGIADYPDDRSDVFHTFFGLAGLSLLGCPQL----K
Gch GRC0126RA00 LGHVDWIDADKLIAFIGRSEDDVDGGFADRPGDMSDVFHTFFALAGLSLLGNKQL----A
Ccr XP_00571301 LGKADWVDARKLREFILACQDEEDGGIADRPGDMADIFHTFFGFAGLSLMEEEGF----E
Pye g4238       LGRVGWIDATALQRFVLGCQDPDAGGVADRVGDLPDVFHTFFGLAALDLLGVGGLGGSGG
Ppu contig_3423 LDKQHWIDANALGAFIMRAQDDEYGGIADRPGDRADVFHTFFGLAGLSLLGHPSL----E
Cre XP_00168980 LGRLHWIDRSALTTFILDCQDEEDGGISDRPDDMADVYHTFFGIAGLSLMGYPNL----A
Kfl GAQ90956.1  MDRVHWIDQKALQRFILDCQDEERGGISDRPDDMVDVFHTYFGVAGLSLLGYPGL----K
Ppa XP_00177348 MERVHWIDRKKLEQFILDCQDPECGGISDRPDDAVDVFHTFFGVAGLSLLGFPGL----A
Smo XP_00296800 LGRVDWIDKEKLKTFILDCQDTEEGGISDRPNDAVDVFHTFFGVAGLSLLDYPGL----K
Ath NP_974770.1 IDRVHWIDKAKLVKFILDCQDLDNGGISDRPEDAVDIFHTYFGVAGLSLLEYPGV----K
                : :  *:*   *  *:  .:*   **.:*   *  *::**:*..*.*.*:    .

Gsu XP_00570604 RIHPAYALPLEII----------------------S
Gch GRC0126RA00 DIHPAYALTTDVVQRVTEP----------------SA
Ccr XP_00571301 KIDPAFALAESVVARIG------------------GG
Pye g4238       GVDPVFALPRQVVARLPAARDLGWGEDGRAPESGSK
Ppu contig_3423 HIHPMYALPCSVIRQLNFSE---------------SA
Cre XP_00168980 AIDPTWALPVEVVERIKRRNE--------AAASASGK
Kfl GAQ90956.1  AIDPAYALPIDVVEKIVRSRR--------TE-----S
Ppa XP_00177348 AIDPAYALPVNVVDRVFYGHK--------KQLAA--S
Smo XP_00296800 RIDAVYALPVDVVKRIF------------------G
Ath NP_974770.1 VIDPAYALPVDVVNRIIF--------------T--K
```
