# Supplementary material for: Unexpected conservation of the RNA splicing apparatus in the highly streamlined genome of Galdieria sulphuraria
Source: BMC Evol Biol. 2018 Apr 2;18:41. doi: 10.1186/s12862-018-1161-x (PMC5880011; doi:10.1186/s12862-018-1161-x)
Supplement: Supplementary file 12 — Figure S6. Estimation of gains and losses of conserved introns in red algal phylogeny. (PDF 92 kb) [file 12862_2018_1161_MOESM12_ESM.pdf]

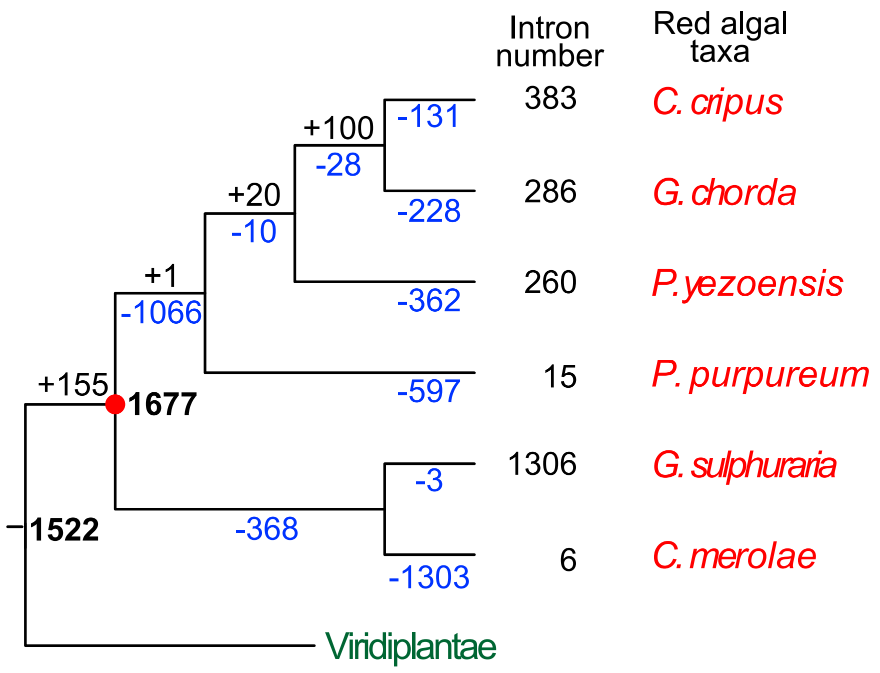

Figure S6. Estimation of gains and losses of conserved introns in red algal phylogeny. The numbers of intron gains (+) and losses (–) are shown above and beneath of the corresponding branches, respectively. The number of conserved intron positions in red algae-Viridiplantae common ancestor and red algal common ancestor are shown in bold text. The estimation was carried out using Dollo parsimony method (1). Intron positions that are unique to each terminal taxon were not included in the analysis. We recognize that parsimony is an unrealistic assumption given the high rate of intron turnover. Nevertheless, the dramatic intron losses that are found independently in *C. merolae* and mesophilic red algae are consistent with the pairwise comparison results in shown Figure 2B.

Reference:

1. Farris J (1977) Phylogenetic analysis under Dollo's law. *Syst Zool* 26(1):77–88.
