# Supplementary material for: Unexpected conservation of the RNA splicing apparatus in the highly streamlined genome of Galdieria sulphuraria
Source: BMC Evol Biol. 2018 Apr 2;18:41. doi: 10.1186/s12862-018-1161-x (PMC5880011; doi:10.1186/s12862-018-1161-x)
Supplement: Supplementary file 13 — Figure S7. The distributions of intron lengths in five red algal species. (PDF 82 kb) [file 12862_2018_1161_MOESM13_ESM.pdf]

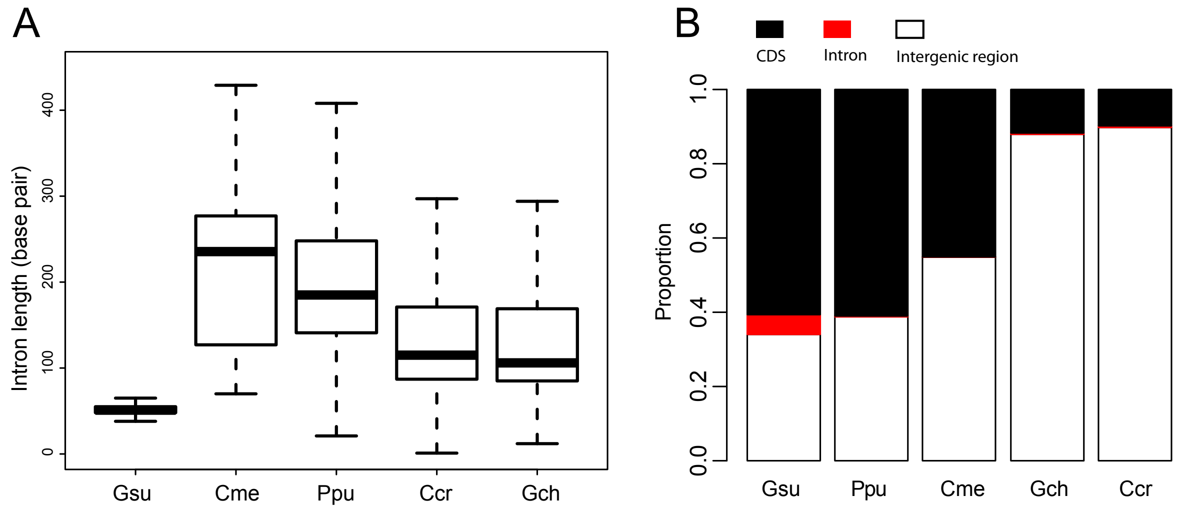

Figure S7. The distributions of intron length and genome compositions in five red algal species. (A) The thick horizontal bars indicate median length whereas the boxes represent inter-quartile range (25%-75%) of the intron lengths for each species. The whiskers extend to the most distant data points that are no more than 1.5 times of the corresponding interquartile ranges from the boxes. Species abbreviations: Gsu (*G. sulphuraria*), Cme (*C. merolae*), Ppu (*P. purpureum*), Ccr (*C. crispus*), and Gch (*G. chorda*), Pye (*P. yezoensis*). (B) The genomic compositions in five red algal species. *G. sulphuraria* and *P. purpureum* have the most compact genomes among the studied species.
