# Supplementary material for: Unexpected conservation of the RNA splicing apparatus in the highly streamlined genome of Galdieria sulphuraria
Source: BMC Evol Biol. 2018 Apr 2;18:41. doi: 10.1186/s12862-018-1161-x (PMC5880011; doi:10.1186/s12862-018-1161-x)
Supplement: Supplementary file 14 — Table S6. Galdieria sulphuraria introns that underwent alternative splicing in our studied samples. (PDF 981 kb) [file 12862_2018_1161_MOESM14_ESM.pdf]

Table S6. ***Galdieria sulphuraria*** **introns that underwent alternative splicing in our studied samples**
*indicates prokaryotic-derived HGTs

No.	Chr	Start	End	Category	coverage (heat)	coverage (cold)	Gene	Product
1	stig_11	197033	200135	alter_trans_start/end	227.182	264.48		
2	stig_12	172606	173433	alter_trans_start/end	262.558	199.669	Gs19100	thylakoid lumen 15.0 kDa protein
3	stig_12	135522	136167	alter_trans_start/end	171.967	144.211		
4	stig_13	143202	143753	alter_trans_start/end	64.8332	90.9179		
5	stig_15	83193	83947	alter_trans_start/end	62.0223	326.757	Gs21670	acyl-CoA oxidase isoform 1
6	stig_17	129770	130309	alter_trans_start/end	31.8651	52.3825	Gs24260	lipoyl(octanoyl) transferase isoform 2
7	stig_20	148087	148187	alter_trans_start/end	200.204	229.763	Gs27370	50S ribosomal protein L33 (mitochondrial)
8	stig_205	3730	4080	alter_trans_start/end	75.4824	153.59		
9	stig_22	70773	70962	alter_trans_start/end	181.507	161.59	Gs29130	DNA binding / methyl-CpG binding protein isoform
10	stig_25	109876	111216	alter_trans_start/end	74.8906	92.1445	Gs32270	haloacid dehalogenase-like hydrolase family protein
11	stig_3	57485	58175	alter_trans_start/end	217.713	208.982	Gs05740	hypothetical protein Gasu_05740
12	stig_31	54024	55179	alter_trans_start/end	58.2705	141.575	Gs36950	BRCA1-associated protein / zinc finger family protein
13	stig_33	112604	113777	alter_trans_start/end	92.838	100.823	Gs38850	V-type H+-transporting ATPase subunit b
14	stig_34	148179	149222	alter_trans_start/end	154.583	152.069	Gs39810	formate--tetrahydrofolate ligase
15	stig_36	64717	65757	alter_trans_start/end	76.4068	182.497	Gs41000	homeobox protein cut-like protein isoform 2
16	stig_38	16145	16464	alter_trans_start/end	492.412	182.688	Gs42260	3R-hydroxymyristoyl ACP dehydrase isoform 1
17	stig_38	49226	49964	alter_trans_start/end	208.063	168.425	Gs42480	ATP-dependent RNA helicase
18	stig_44	69389	69803	alter_trans_start/end	102.992	136.864	Gs47250	cis-prenyltransferase, dehydrodolichyl diphosphate synthase
19	stig_46	112436	112832	alter_trans_start/end	105.848	125.065	Gs49010	DNA excision repair protein ERCC-2
20	stig_50	45779	46486	alter_trans_start/end	82.2424	189.223	Gs51320	O-acyl transferase, membrane bound
21	stig_55	18074	21133	alter_trans_start/end	218.103	145.451	Gs54110	ubiquinone/menaquinone biosynthesis methyltransferase
22	stig_55	78858	79819	alter_trans_start/end	97.5576	88.9046	Gs54460	aspartyl protease
23	stig_57	52662	52984	alter_trans_start/end	78.3484	92.5134	Gs55450	hypothetical protein Gasu_55450
24	stig_6	238115	238549	alter_trans_start/end	85.334	83.8542	Gs11350	ubiquitin carboxyl-terminal hydrolase 4/11/15
25	stig_6	143315	144123	alter_trans_start/end	129.566	93.6686	Gs10850	hypothetical protein Gasu_10850
26	stig_60	76676	77311	alter_trans_start/end	79.1716	98.4837	Gs57160	mitotic spindle assembly checkpoint protein MAD1
27	stig_65	996	2976	alter_trans_start/end	790.672	229.202	Gs59090	phosphoserine phosphatase
28	stig_7	158838	161133	alter_trans_start/end	105.257	149.23	Gs12490	lycopene beta cyclase
29	stig_8	136934	138932	alter_trans_start/end	75.9771	135.911	Gs13750	ADP-ribosylation factor GTPase-activating protein 1
30	stig_8	71977	73783	alter_trans_start/end	128.762	168.751	Gs13410	hypothetical protein Gasu_13410
31	stig_17	45986	46138	exon_skipping	342.875	169.45	Gs23800	diamine N-acetyltransferase
32	stig_21	140919	140976	exon_skipping	193.906	179.633	Gs28420	hypothetical protein isoform 1
33	stig_25	143979	144028	exon_skipping	302.514	327.567		
34	stig_3	57114	57173	exon_skipping	426.177	310.009	Gs05730	hypothetical protein Gasu_05730
35	stig_34	167927	167986	exon_skipping	250.008	761.092		
36	stig_39	104368	104444	exon_skipping	1389.42	1311.65	Gs43500	3-isopropylmalate dehydratase small subunit
37	stig_40	108854	108909	exon_skipping	1568.74	1145.14	Gs44280	thioredoxin f

38	stig_48	75537	75598	exon_skipping	288.95	274.812	Gs50150	UDPglucose 6-dehydrogenase isoform 2
39	stig_53	7887	7950	exon_skipping	318.811	329.033		
40	stig_56	83049	83162	exon_skipping	484.482	495.002	Gs54960	electron carrier protein (proton gradient regulation 5) isoform 1
41	stig_7	53192	53422	exon_skipping	1109.58	682.769	Gs11750	hypothetical protein Gasu_11750
42	stig_0	199569	199619	intron_retention	108.594	196.586	Gs01030	CoA-transferase family protein
43	stig_0	40164	40218	intron_retention	415.011	243.037	Gs00250	DNA topoisomerase II
44	stig_0	236227	236278	intron_retention	602.633	213.024	Gs01220	bifunctional phosphoribosylaminoimidazole carboxylase
45	stig_0	199841	199908	intron_retention	90.1034	139.654	Gs01030	CoA-transferase family protein
46	stig_0	49448	49518	intron_retention	135.486	109.37	Gs00300	cornichon family protein
47	stig_0	110478	110524	intron_retention	96.0344	73.5864	Gs00630	hypothetical protein Gasu_00630
48	stig_0	29525	29574	intron_retention	182.097	266.045	Gs00180	thioredoxin family Trp26-like protein
49	stig_0	37217	37284	intron_retention	136.007	246.137	Gs00240	hypothetical protein Gasu_00240
50	stig_0	248675	248728	intron_retention	208.523	357.487	Gs01300	hypothetical protein isoform 1
51	stig_0	247658	247712	intron_retention	288.321	223.255	Gs01290	diphosphoinositol polyphosphate phosphohydrolase-like protein
52	stig_0	431507	431558	intron_retention	60.319	89.0397		
53	stig_0	436468	436540	intron_retention	266.186	201.131		
54	stig_0	388340	388398	intron_retention	61.8076	116.523	Gs02020	PAB-dependent poly(A)-specific ribonuclease subunit 2
55	stig_0	108391	108438	intron_retention	97.0388	92.1198	Gs00610	dolichol-phosphate mannosyltransferase
56	stig_0	239592	239638	intron_retention	121.527	242.742	Gs01240	16S rRNA processing protein RimM
57	stig_0	239391	239443	intron_retention	85.4125	159.162	Gs01240	16S rRNA processing protein RimM
58	stig_0	239739	239795	intron_retention	148.989	284.414	Gs01240	16S rRNA processing protein RimM
59	stig_0	153465	153521	intron_retention	427.375	469.05	Gs00780	hypothetical protein Gasu_00780
60	stig_0	343389	343439	intron_retention	51.574	87.3998	Gs01860	prefoldin, putative isoform 1
61	stig_0	309831	309883	intron_retention	192.184	139.21	Gs01690	D-3-phosphoglycerate dehydrogenase
62	stig_0	346678	346728	intron_retention	143.83	204.546	Gs01880	trehalose-6-phosphate synthase, putative
63	stig_0	105910	105957	intron_retention	237.005	126.949	Gs00590	CAAX amino terminal protease family protein
64	stig_0	240170	240223	intron_retention	94.2677	144.411	Gs01250	hypothetical protein Gasu_01250
65	stig_0	103802	103884	intron_retention	165.403	212.256	Gs00580	beta-phosphoglucomutase isoform 1
66	stig_0	327338	327388	intron_retention	250.989	200.033	Gs01780	hypothetical protein Gasu_01780
67	stig_0	433844	433899	intron_retention	91.8127	110.22	Gs02300	sugar-phosphate:phosphate translocator
68	stig_0	129024	129070	intron_retention	76.9532	79.9374	Gs00700	protein geranylgeranyltransferase type II
69	stig_0	136589	136640	intron_retention	111.951	92.7759	Gs00720	hypothetical protein Gasu_00720
70	stig_0	240323	240380	intron_retention	62.0567	86.5464	Gs01250	hypothetical protein Gasu_01250
71	stig_0	350842	350902	intron_retention	80.402	163.659		
72	stig_0	309620	309670	intron_retention	239.202	182.673	Gs01690	D-3-phosphoglycerate dehydrogenase
73	stig_0	301892	301943	intron_retention	140.038	71.0163	Gs01650	hypothetical protein Gasu_01650
74	stig_0	291129	291176	intron_retention	292.04	198.916	Gs01560	peptidyl-prolyl cis-trans isomerase B (cyclophilin B)
75	stig_0	108182	108230	intron_retention	103.722	86.0424	Gs00610	dolichol-phosphate mannosyltransferase
76	stig_0	276631	276679	intron_retention	189.635	195.055	Gs01480	mitochondrial protein translocase, MPT family
77	stig_0	357814	357863	intron_retention	104.439	26.7067	Gs01940	hypothetical protein isoform 1
78	stig_0	351567	351616	intron_retention	32.4963	88.5832	Gs01910	O-phospho-L-seryl-tRNA(Sec):L-selenocysteinyl-tRNA synthase isoform1

79	stig_0	283345	283404	intron_retention	414.602	525.719	Gs01520	glycerate kinase
80	stig_0	302156	302206	intron_retention	265.967	128.085	Gs01650	hypothetical protein Gasu_01650
81	stig_0	41524	41587	intron_retention	77.3476	81.2325	Gs00250	DNA topoisomerase II
82	stig_0	347001	347052	intron_retention	87.1634	115.624		
83	stig_0	248901	248963	intron_retention	88.6346	214.961	Gs01300	hypothetical protein isoform 1
84	stig_0	186304	186364	intron_retention	86.4075	57.6278		
85	stig_0	290836	290882	intron_retention	380.73	248.817	Gs01560	peptidyl-prolyl cis-trans isomerase B (cyclophilin B)
86	stig_0	168658	168700	intron_retention	52.7043	87.4098	Gs00870	hypothetical protein Gasu_00870
87	stig_0	388974	389041	intron_retention	189.492	227.958	Gs02020	PAB-dependent poly(A)-specific ribonuclease subunit 2
88	stig_0	357640	357695	intron_retention	136.551	52.6253	Gs01940	hypothetical protein isoform 1
89	stig_0	277230	277287	intron_retention	552.893	472.78	Gs01480	mitochondrial protein translocase, MPT family
90	stig_0	437367	437415	intron_retention	419.093	308.422	Gs02320	prohibitin
91	stig_0	21088	21153	intron_retention	81.6838	127.219	Gs00130*	alpha-glucosidase
92	stig_0	340151	340196	intron_retention	71.4594	72.7586		
93	stig_0	143171	143222	intron_retention	78.8535	66.3276		
94	stig_0	222737	222792	intron_retention	205.105	151.585	Gs01140	hypothetical protein Gasu_01140
95	stig_0	254979	255040	intron_retention	567.184	357.59	Gs01340	Myb-like DNA-binding protein BAS1
96	stig_0	383815	383875	intron_retention	78.8912	119.272	Gs02010	alpha-1,3-mannosyl-glycoproteinbeta-1,2-N-acetylglucosaminyltransferase isoform 2
97	stig_0	434061	434114	intron_retention	59.7574	108.971	Gs02300	sugar-phosphate:phosphate translocator, DMT family isoform 1
98	stig_0	103553	103605	intron_retention	195.112	236.708	Gs00580	beta-phosphoglucomutase isoform 1
99	stig_0	211570	211627	intron_retention	90.4005	119.297	Gs01100	hypothetical protein Gasu_01100
100	stig_0	225015	225072	intron_retention	507.525	403.572		
101	stig_0	350262	350324	intron_retention	25.0492	56.7372	Gs01900	hypothetical protein isoform 1
102	stig_0	74201	74252	intron_retention	77.338	93.2953	Gs00420	NADH dehydrogenase I alpha subcomplex assembly factor1-like protein
103	stig_0	251499	251562	intron_retention	160.936	278.572	Gs01310	hypothetical protein Gasu_01310
104	stig_0	389407	389457	intron_retention	98.6953	117.422		
105	stig_1	33164	33226	intron_retention	59.6978	24.2893	Gs02550	adenylate kinase
106	stig_1	38372	38430	intron_retention	370.321	472.326	Gs02580	DNA repair protein isoform 2
107	stig_1	60516	60568	intron_retention	259.975	429.061	Gs02740	shikimate kinase family protein
108	stig_1	227258	227322	intron_retention	262.165	371.228	Gs03620	ADP-ribosylation factor isoform 2
109	stig_1	295396	295459	intron_retention	80.1695	206.207	Gs03920	hypothetical protein Gasu_03920
110	stig_1	227125	227180	intron_retention	271.935	371.047	Gs03620	ADP-ribosylation factor isoform 2
111	stig_1	194599	194661	intron_retention	247.62	869.409	Gs03450	membrane bound O-acyl transferase (MBOAT) family
112	stig_1	45204	45253	intron_retention	33.7656	74.703		
113	stig_1	8994	9044	intron_retention	368.006	286.043	Gs02440	DNA-directed RNA polymerase II subunit D
114	stig_1	48740	48792	intron_retention	125.636	243.71	Gs02660	hypothetical protein Gasu_02660
115	stig_1	221123	221172	intron_retention	167.421	166.656	Gs03590	hypothetical protein Gasu_03590
116	stig_1	151692	151747	intron_retention	137.713	181.592	Gs03230	hypothetical protein isoform 2
117	stig_1	124340	124389	intron_retention	62.5511	129.547		
118	stig_1	152199	152247	intron_retention	111.572	131.955	Gs03240	hypothetical protein isoform 1
119	stig_1	27489	27543	intron_retention	127.265	135.81	Gs02520	proton-dependent oligopeptide transporter, POT family

120	stig_1	38059	38118	intron_retention	177.277	258.193	Gs02580	DNA repair protein isoform 2
121	stig_1	193677	193728	intron_retention	31.8698	121.952		
122	stig_1	295756	295836	intron_retention	123.244	256.205		
123	stig_1	207966	208020	intron_retention	159.737	195.756		
124	stig_1	38257	38309	intron_retention	285.441	381.596	Gs02580	DNA repair protein isoform 2
125	stig_1	142644	142690	intron_retention	77.0064	88.4519	Gs03200	hypothetical protein Gasu_03200
126	stig_1	124066	124123	intron_retention	57.1326	108.01		
127	stig_1	227644	227692	intron_retention	149.138	218.242	Gs03620	ADP-ribosylation factor isoform 2
128	stig_1	233720	233774	intron_retention	198.242	335.065	Gs03640	MYB-related protein
129	stig_1	14805	14857	intron_retention	246.352	171.723	Gs02470	CTP synthase isoform 1
130	stig_1	112221	112291	intron_retention	72.4792	69.383		
131	stig_1	30694	30745	intron_retention	753.7	781.788	Gs02540	hypothetical protein Gasu_02540
132	stig_1	89376	89426	intron_retention	112.143	160.352	Gs02930	glucosidase/ hydrolase, hydrolyzing O-glycosyl
133	stig_1	235864	235917	intron_retention	142.328	86.1866	Gs03660	mRNA guanylyltransferase
134	stig_1	299916	299969	intron_retention	135.389	132.041		
135	stig_1	46142	46196	intron_retention	262.687	240.045	Gs02640	hypothetical protein isoform 2
136	stig_1	41832	41886	intron_retention	79.9214	63.8384	Gs02600	hypothetical protein Gasu_02600
137	stig_1	132486	132536	intron_retention	179.47	154.085	Gs03120	hypothetical protein Gasu_03120
138	stig_1	54700	54754	intron_retention	182.364	336.432	Gs02710	cytochrome c-type biogenesis protein
139	stig_1	48118	48171	intron_retention	80.0442	127.562	Gs02660	hypothetical protein Gasu_02660
140	stig_1	235438	235486	intron_retention	46.7115	55.1983	Gs03660	mRNA guanylyltransferase
141	stig_1	81228	81276	intron_retention	321.285	100.639		
142	stig_1	83226	83300	intron_retention	116.269	217.14	Gs02880	minichromosome maintenance family (MCM)
143	stig_1	14441	14493	intron_retention	233.973	231.053	Gs02470	CTP synthase isoform 1
144	stig_1	14610	14662	intron_retention	256.122	232.051	Gs02470	CTP synthase isoform 1
145	stig_1	158384	158453	intron_retention	189.772	170.233		
146	stig_1	295474	295525	intron_retention	102.812	245.64	Gs03920	hypothetical protein Gasu_03920
147	stig_1	281013	281073	intron_retention	138.264	66.1268		
148	stig_10	89890	89943	intron_retention	162.205	168.457		
149	stig_10	79328	79377	intron_retention	498.29	43.8429	Gs16000	ABC transporter, ATP-binding protein isoform 1
150	stig_10	71822	71875	intron_retention	169.97	240.288	Gs15960	hypothetical protein Gasu_15960
151	stig_10	107920	107964	intron_retention	62.778	296.067		
152	stig_10	105153	105208	intron_retention	568.203	453.785	Gs16130	3'(2'), 5'-bisphosphate nucleotidase
153	stig_10	168660	168708	intron_retention	245.825	371.564	Gs16400	dihydroneopterin aldolase
154	stig_10	168456	168515	intron_retention	187.483	270.523	Gs16400	dihydroneopterin aldolase
155	stig_10	183574	183626	intron_retention	115.824	133.134	Gs16520	hypothetical protein Gasu_16520
156	stig_10	96415	96463	intron_retention	220.955	480.71	Gs16090	cytochrome b5
157	stig_10	114906	114971	intron_retention	87.0386	102.136		
158	stig_10	40485	40532	intron_retention	119.872	198.809	Gs15820	serine/threonine protein kinase
159	stig_10	174039	174084	intron_retention	157.017	187.087	Gs16440*	2-dehydropantoate 2-reductase isoform 2
160	stig_10	114654	114709	intron_retention	165.151	236.271	Gs16180	hypothetical protein Gasu_16180
161	stig_10	97716	97763	intron_retention	241.208	145.059	Gs16100	glutamate decarboxylase isoform 2
162	stig_10	198025	198084	intron_retention	97.5684	110.525		
163	stig_10	73119	73172	intron_retention	32.3658	36.6093	Gs15970	Myb-like DNA-binding protein REB1

164	stig_10	97512	97576	intron_retention	207.279	114.771	Gs16100	glutamate decarboxylase isoform 2
165	stig_10	90952	91006	intron_retention	162.541	188.254	Gs16070	isocitrate dehydrogenase, NADP dependent
166	stig_10	33767	33823	intron_retention	329.382	280.36	Gs15780*	peptidase S58 DmpA isoform 1
167	stig_10	97101	97153	intron_retention	79.2585	91.7254	Gs16100	glutamate decarboxylase isoform 2
168	stig_10	98002	98056	intron_retention	430.318	245.212	Gs16100	glutamate decarboxylase isoform 2
169	stig_10	40741	40798	intron_retention	198.921	316.658	Gs15820	serine/threonine protein kinase
170	stig_10	115240	115308	intron_retention	55.2161	76.7813	Gs16190	telomerase reverse transcriptase isoform 2
171	stig_10	212218	212295	intron_retention	51.4424	117.091		
172	stig_10	135163	135234	intron_retention	24.7434	62.4033		
173	stig_10	79661	79728	intron_retention	350.346	76.7572	Gs16000	ABC transporter, ATP-binding protein isoform 1
174	stig_10	55794	55850	intron_retention	88.4599	120.506	Gs15900	H+-translocating PPase (vacuolar)
175	stig_10	115068	115121	intron_retention	85.6042	98.6249	Gs16190	telomerase reverse transcriptase isoform 2
176	stig_100	8296	8355	intron_retention	76.7591	125.812		
177	stig_11	125120	125168	intron_retention	455.92	279.094	Gs17520	oxidoreductase, acting on the CH-CH group of donors
178	stig_11	196514	196571	intron_retention	203.22	310.148	Gs17960	hypothetical protein isoform 1
179	stig_11	204729	204782	intron_retention	136.672	231.389		
180	stig_11	105949	105992	intron_retention	113.504	115.336	Gs17410	hypothetical protein Gasu_17410
181	stig_11	78419	78628	intron_retention	161.265	123.075	Gs17210	ATP phosphoribosyltransferase
182	stig_11	98015	98060	intron_retention	383.526	318.466	Gs17350	hypothetical protein isoform 2
183	stig_11	114866	114916	intron_retention	193.938	279.649	Gs17470	monooxygenase/ oxidoreductase
184	stig_11	3087	3143	intron_retention	382.022	419.163	Gs16780	hypothetical protein isoform 2
185	stig_11	153573	153620	intron_retention	277.331	355.093	Gs17690*	tRNA/rRNA methyltransferase isoform 1
186	stig_11	99164	99223	intron_retention	1440.4	447.417	Gs17360	short-chain dehydrogenase/reductase SDR
187	stig_11	167137	167190	intron_retention	152.883	240.095	Gs17780	NAD+ diphosphatase
188	stig_11	77279	77325	intron_retention	307.564	155.065	Gs17200	prefoldin subunit 2
189	stig_11	124895	124942	intron_retention	562.61	386.184	Gs17520	oxidoreductase, acting on the CH-CH group of donors
190	stig_11	210163	210224	intron_retention	401.857	513.749		
191	stig_11	214890	214944	intron_retention	63.383	104.605		
192	stig_11	59363	59416	intron_retention	142.028	110.413	Gs17120	carboxymethylenebutenolidase
193	stig_11	153036	153111	intron_retention	43.654	230.567		
194	stig_11	98704	98759	intron_retention	48.4011	77.0267	Gs17360	short-chain dehydrogenase/reductase SDR
195	stig_11	17742	17792	intron_retention	89.8667	106.124		
196	stig_11	97832	97887	intron_retention	192.754	191.722	Gs17350	hypothetical protein isoform 2
197	stig_11	20488	20548	intron_retention	122.559	109.845	Gs16900	20S proteasome subunit alpha 7
198	stig_11	193849	193913	intron_retention	320.958	301.574	Gs17940	zinc transporter, ZIP family
199	stig_11	168141	168202	intron_retention	42.4522	84.6551	Gs17780	NAD+ diphosphatase
200	stig_115	285	335	intron_retention	47.4085	37.1754		
201	stig_12	135754	135911	intron_retention	138.674	125.878		
202	stig_12	114640	114690	intron_retention	411.195	151.966	Gs18740	hypothetical protein Gasu_18740
203	stig_12	108461	108523	intron_retention	59.3002	111.127		
204	stig_12	111266	111313	intron_retention	156.468	125.309	Gs18710	hypothetical protein isoform 2
205	stig_12	195175	195229	intron_retention	169.491	328.561		
206	stig_12	60751	60794	intron_retention	41.5159	30.5278	Gs18390	programmed cell death 6-interacting protein
207	stig_12	179874	179925	intron_retention	9.8928	74.5803	Gs19140	hypothetical protein Gasu_19140

208	stig_12	134613	134657	intron_retention	204.447	235.151	Gs18860	aldo/keto reductase
209	stig_12	149479	149527	intron_retention	139.686	177.925	Gs18940	hypothetical protein Gasu_18940
210	stig_12	22307	22358	intron_retention	884.812	591.035	Gs18190	sucrose transporter, GPH family isoform 2
211	stig_12	141825	141882	intron_retention	207.12	292.462	Gs18910	E3 ubiquitin-protein ligase BRE1
212	stig_12	154465	154513	intron_retention	394.805	326.114	Gs18960	delta14-sterol reductase
213	stig_12	57411	57461	intron_retention	83.7663	105.491		
214	stig_12	114761	114818	intron_retention	236.015	90.8208	Gs18740	hypothetical protein Gasu_18740
215	stig_12	76166	76219	intron_retention	75.1251	71.3648	Gs18510	DNA polymerase delta subunit 2
216	stig_12	53430	53484	intron_retention	67.8797	84.5176		
217	stig_12	49025	49078	intron_retention	151.828	109.933	Gs18320	exosome complex component RRP43
218	stig_12	73308	73365	intron_retention	202.764	139.945	Gs18490	7,8-dihydro-8-oxoguanine triphosphatase
219	stig_12	66255	66307	intron_retention	131.187	75.793		
220	stig_12	127580	127636	intron_retention	163.973	114.838		
221	stig_12	131157	131205	intron_retention	102.602	168.042		
222	stig_12	121375	121430	intron_retention	867.7	417.612	Gs18800	hypothetical protein Gasu_18800
223	stig_126	5859	5936	intron_retention	20.8156	70.1458		
224	stig_13	170379	170431	intron_retention	83.9807	105.412	Gs20150	mRNA splicing factor, putative isoform 1
225	stig_13	112273	112321	intron_retention	355.448	376.62	Gs19820	mitochondrial ribosomal protein L46
226	stig_13	157926	157977	intron_retention	190.049	113.936	Gs20080	FtsJ-like methyltransferase family protein
227	stig_13	29090	29138	intron_retention	54.5927	54.4845	Gs19340	hypothetical protein Gasu_19340
228	stig_13	79407	79467	intron_retention	431.635	217.937	Gs19620	methionyl-tRNA synthetase isoform 1
229	stig_13	152479	152527	intron_retention	97.001	169.23	Gs20050	hypothetical protein isoform 2
230	stig_13	152612	152664	intron_retention	142.962	212.651	Gs20050	hypothetical protein isoform 2
231	stig_13	200793	200847	intron_retention	49.3094	96.6156		
232	stig_13	128985	129030	intron_retention	47.8954	70.0002	Gs19940	G-protein coupled receptor activity
233	stig_13	54946	54995	intron_retention	532.022	324.158		
234	stig_13	68235	68288	intron_retention	166.946	85.5943	Gs19560	hypothetical protein Gasu_19560
235	stig_13	151912	151957	intron_retention	37.3381	63.6484	Gs20050	hypothetical protein isoform 2
236	stig_13	165785	165835	intron_retention	483.032	332.624	Gs20120	cell division protein FtsZ
237	stig_13	152359	152406	intron_retention	71.9752	125.487	Gs20050	hypothetical protein isoform 2
238	stig_13	118577	118632	intron_retention	104.479	108.112	Gs19870	hypothetical protein Gasu_19870
239	stig_13	29989	30037	intron_retention	172.791	366.66	Gs19350	methionyl-tRNA synthetase isoform 1
240	stig_13	64958	65017	intron_retention	77.3553	271.48		
241	stig_13	51188	51239	intron_retention	119.471	127.461	Gs19470	dimethyladenosine transferase
242	stig_13	134150	134203	intron_retention	446.709	212.095	Gs19980	cyclin-dependent kinase regulatory subunit CKS1
243	stig_13	153070	153122	intron_retention	196.265	278.91	Gs20050	hypothetical protein isoform 2
244	stig_13	64792	64842	intron_retention	74.2943	233.326		
245	stig_13	152917	152977	intron_retention	199.756	295.158	Gs20050	hypothetical protein isoform 2
246	stig_13	186626	186686	intron_retention	302.396	544.972	Gs20190	ubiquitin-conjugating enzyme E2
247	stig_13	186962	187027	intron_retention	248.091	411.004	Gs20190	ubiquitin-conjugating enzyme E2
248	stig_13	170966	171022	intron_retention	174.045	201.217	Gs20150	mRNA splicing factor, putative isoform 1
249	stig_13	154283	154337	intron_retention	247.455	238.928		
250	stig_13	101947	101998	intron_retention	164.236	191.023	Gs19760	aminoacyl-tRNA hydrolase
251	stig_137	2646	2694	intron_retention	144.929	131.555	Gs62740	protein kinase / protein phosphatase 2C

252	stig_14	94778	94825	intron_retention	164.073	76.2687	Gs20700	chromosome transmission fidelity protein 8 isoform 2
253	stig_14	34073	34122	intron_retention	87.3873	138.787	Gs20410	RNA-binding protein
254	stig_14	35801	35849	intron_retention	260.908	215.559		
255	stig_14	112248	112328	intron_retention	346.453	208.355		
256	stig_14	116126	116177	intron_retention	273.414	235.857	Gs20830	rhomboid-like protein isoform 2
257	stig_14	8333	8378	intron_retention	172.646	101.304	Gs20290	hypothetical protein Gasu_20290
258	stig_14	75211	75262	intron_retention	220.875	297.579	Gs20600	hypothetical protein Gasu_20600
259	stig_14	35938	35991	intron_retention	220.236	194.481	Gs20420*	hypothetical protein Gasu_20420
260	stig_14	33477	33526	intron_retention	129.974	223.871	Gs20410	RNA-binding protein
261	stig_14	17135	17193	intron_retention	66.3486	76.6687		
262	stig_14	191988	192048	intron_retention	108.013	111.689		
263	stig_14	15708	15760	intron_retention	179.261	94.6709	Gs20330	dolichol-phosphate mannosyltransferase
264	stig_14	61239	61287	intron_retention	228.021	118.327	Gs20540	mitochondrial carrier (BOU / S-adenosylmethionine carrier)
265	stig_14	61126	61177	intron_retention	581.881	264.809	Gs20540	mitochondrial carrier (BOU / S-adenosylmethionine carrier)
266	stig_14	31869	31914	intron_retention	74.1502	88.4501	Gs20390	hypothetical protein Gasu_20390
267	stig_14	92647	92702	intron_retention	175.196	135.514		
268	stig_14	111789	111835	intron_retention	206.441	135.145	Gs20800	asparaginyl-tRNA synthetase isoform 1
269	stig_14	143729	143795	intron_retention	224.706	117.702	Gs21030	short chain dehydrogenase/reductase family oxidoreductase
270	stig_14	35583	35632	intron_retention	135.354	132.048		
271	stig_142	5085	5139	intron_retention	41.129	91.7779		
272	stig_149	333	403	intron_retention	39.3125	73.6715		
273	stig_15	159693	159742	intron_retention	220.203	409.428	Gs22080	sterol-4alpha-carboxylate 3-dehydrogenase (decarboxylating)
274	stig_15	137504	137561	intron_retention	255.848	236.789	Gs21980	hypothetical protein Gasu_21980
275	stig_15	184665	184717	intron_retention	111.939	425.394	Gs22200	hypothetical protein Gasu_22200
276	stig_15	50282	50339	intron_retention	250.649	220.64	Gs21460	RNA cytosine methyltransferase
277	stig_15	125016	125067	intron_retention	308.329	121.05	Gs21930	chorismate synthase
278	stig_15	66685	66730	intron_retention	39.8856	55.4187	Gs21560	hypothetical protein Gasu_21560
279	stig_15	94264	94320	intron_retention	170.241	145.859	Gs21730	3-hydroxyisobutyryl-CoA hydrolase
280	stig_15	110493	110547	intron_retention	389.715	344.32	Gs21830	hypothetical protein Gasu_21830
281	stig_15	68498	68545	intron_retention	165.828	165.42	Gs21570	protein binding protein / zinc ion binding protein
282	stig_15	98745	98804	intron_retention	285.543	295.777	Gs21770	inositol phosphatase/fructose-16-bisphosphatase
283	stig_15	120139	120185	intron_retention	90.3255	51.8345	Gs21900	hypothetical protein Gasu_21900
284	stig_15	126571	126626	intron_retention	264.519	248.744	Gs21940	hypothetical protein Gasu_21940
285	stig_15	55019	55071	intron_retention	344.063	357.532	Gs21480	50S ribosomal protein L24 (mitochondrial)
286	stig_15	176313	176364	intron_retention	326.637	221.798	Gs22150	hypothetical protein Gasu_22150
287	stig_15	127388	127450	intron_retention	81.1186	137.244	Gs21940	hypothetical protein Gasu_21940
288	stig_15	98621	98668	intron_retention	174.725	314.505		
289	stig_15	54819	54871	intron_retention	140.705	162.268	Gs21480	50S ribosomal protein L24 (mitochondrial)
290	stig_15	165265	165320	intron_retention	1127.34	616.67	Gs22100	profilin
291	stig_15	110834	110897	intron_retention	278.267	305.674	Gs21830	hypothetical protein Gasu_21830

292	stig_15	170119	170170	intron_retention	891.89	978.957	Gs22120	succinyl-CoA synthetase beta subunit
293	stig_15	35469	35519	intron_retention	112.989	190.862	Gs21350	NADH dehydrogenase
294	stig_15	29179	29235	intron_retention	65.232	72.1333		
295	stig_15	98431	98490	intron_retention	104.409	254.396	Gs21760	zinc finger protein
296	stig_15	165556	165605	intron_retention	393.77	398.462	Gs22110	alpha,alpha-trehalose-phosphate synthase (UDP-forming)
297	stig_15	66559	66616	intron_retention	355.978	521.083	Gs21560	hypothetical protein Gasu_21560
298	stig_151	2216	2276	intron_retention	100.856	114.322	Gs63020	S-adenosylmethionine-dependent methyltransferase
299	stig_155	4695	4743	intron_retention	209.228	273.113	Gs63090	hypothetical protein Gasu_63090
300	stig_155	4244	4294	intron_retention	351.033	445.871	Gs63090	hypothetical protein Gasu_63090
301	stig_155	4367	4420	intron_retention	393.428	540.987	Gs63090	hypothetical protein Gasu_63090
302	stig_159	3200	3262	intron_retention	120.773	125.886	Gs63180	aldehyde dehydrogenase (NAD+)
303	stig_16	23985	24034	intron_retention	149.646	236.247	Gs22480	hypothetical protein Gasu_22480
304	stig_16	177288	177333	intron_retention	75.0941	91.4039	Gs23390	zinc-containing alcohol dehydrogenase
305	stig_16	181312	181367	intron_retention	296.432	499.544	Gs23430	ribonuclease Z
306	stig_16	54343	54397	intron_retention	76.354	109.9		
307	stig_16	169626	169688	intron_retention	56.1129	43.6156	Gs23340	NAD+ synthase (glutamine-hydrolysing) isoform 2
308	stig_16	142397	142461	intron_retention	283.759	375.59	Gs23190	LAO/AO transport system kinase isoform 2
309	stig_16	192070	192118	intron_retention	113.77	228.141		
310	stig_16	111618	111667	intron_retention	473.623	550.857	Gs22990	hypothetical protein Gasu_22990
311	stig_16	66447	66492	intron_retention	302.429	297.206	Gs22730	mitochondrial protein translocase, MPT family
312	stig_16	198995	199049	intron_retention	174.07	205.49		
313	stig_16	158131	158189	intron_retention	136.722	176.375		
314	stig_16	54695	54745	intron_retention	100.149	110.643	Gs22660	hypothetical protein isoform 2
315	stig_16	176359	176405	intron_retention	35.0651	110.083		
316	stig_16	38388	38437	intron_retention	332.319	320.623	Gs22570	aminoacylase
317	stig_16	167833	167888	intron_retention	251.551	240.582	Gs23330	nicotinamide-nucleotide adenylyltransferase
318	stig_16	168054	168107	intron_retention	148.932	134.914	Gs23330	nicotinamide-nucleotide adenylyltransferase
319	stig_16	91645	91696	intron_retention	183.573	323.515	Gs22890	hypothetical protein Gasu_22890
320	stig_16	169307	169362	intron_retention	247.853	202.546	Gs23340	NAD+ synthase (glutamine-hydrolysing) isoform 2
321	stig_16	23847	23892	intron_retention	169.395	275.21	Gs22480	hypothetical protein Gasu_22480
322	stig_16	171361	171413	intron_retention	117.348	81.9096	Gs23350	hypothetical protein isoform 2
323	stig_16	135297	135352	intron_retention	76.8403	31.9087	Gs23130	mannosyl-oligosaccharide glucosidase
324	stig_16	178539	178594	intron_retention	113.693	97.1569	Gs23400	hypothetical protein Gasu_23400
325	stig_16	121798	121847	intron_retention	138.868	123.35	Gs23050	hypothetical protein Gasu_23050
326	stig_16	145584	145662	intron_retention	177.195	244.519	Gs23200	hormone-sensitive lipase
327	stig_16	105447	105498	intron_retention	201.437	383.71	Gs22950	hypothetical protein Gasu_22950
328	stig_16	29128	29180	intron_retention	175.679	148.491	Gs22510	nucleic acid binding protein
329	stig_16	176846	176902	intron_retention	35.3895	54.2077	Gs23390	zinc-containing alcohol dehydrogenase
330	stig_16	139471	139539	intron_retention	68.0171	200.601	Gs23170	hypothetical protein Gasu_23170
331	stig_16	121929	121984	intron_retention	227.166	504.359	Gs23050	hypothetical protein Gasu_23050
332	stig_16	201102	201159	intron_retention	32.8721	100.512		
333	stig_16	198575	198633	intron_retention	195.836	226.929	Gs23500	hypothetical protein Gasu_23500
334	stig_16	115614	115661	intron_retention	419.506	244.911	Gs23020	protein arginine N-methyltransferase 1
335	stig_16	191902	191964	intron_retention	162.084	312.823		

336	stig_16	187984	188044	intron_retention	544.145	287.915		
337	stig_16	117843	117895	intron_retention	1114.59	1876.46	Gs23030	histone H1
338	stig_16	172571	172625	intron_retention	77.8209	68.2975	Gs23360	hypothetical protein Gasu_23360
339	stig_16	145732	145797	intron_retention	400.383	370.846		
340	stig_16	4415	4464	intron_retention	302.004	219.607	Gs22340	protoporphyrinogen oxidase
341	stig_16	94457	94514	intron_retention	74.2541	96.4493	Gs22900	DNA mismatch repair protein PMS2
342	stig_16	165290	165354	intron_retention	49.8956	62.8551	Gs23310	sulfate permease, SulP family
343	stig_16	168963	169012	intron_retention	205.07	211.462	Gs23340	NAD+ synthase (glutamine-hydrolysing) isoform 2
344	stig_16	180753	180804	intron_retention	691.415	1225.3	Gs23420	hypothetical protein Gasu_23420
345	stig_162	3793	3846	intron_retention	106.996	175.454	Gs63230	myb family transcription factor
346	stig_162	3080	3128	intron_retention	264.617	213.631	Gs63220	hypothetical protein isoform 1
347	stig_162	3191	3235	intron_retention	173.159	173.404	Gs63220	hypothetical protein isoform 1
348	stig_164	906	958	intron_retention	357.386	193.161	Gs63270	arsenate reductase
349	stig_164	771	819	intron_retention	349.651	156.547	Gs63270	arsenate reductase
350	stig_17	70712	70765	intron_retention	144.646	133.6	Gs23920	hypothetical protein isoform 1
351	stig_17	24037	24087	intron_retention	113.824	190.479	Gs23680	NADH:ubiquinone oxidoreductase family protein
352	stig_17	130622	130689	intron_retention	51.1143	73.5547	Gs24260	lipoyl(octanoyl) transferase isoform 2
353	stig_17	190457	190508	intron_retention	223.214	532.647	Gs24590	carbonyl reductase (NADPH)
354	stig_17	145553	145620	intron_retention	326.901	558.706	Gs24350	tRNA-intron endonuclease
355	stig_17	199338	199402	intron_retention	107.066	129.222		
356	stig_17	105976	106029	intron_retention	110.049	228.627	Gs24120	putative glutathione S-transferase isoform 1
357	stig_17	116505	116572	intron_retention	458.08	346.605	Gs24190	prolyl-tRNA synthetase
358	stig_17	157341	157386	intron_retention	152.714	137.651	Gs24390	endonuclease
359	stig_17	96754	96803	intron_retention	118.378	76.0597	Gs24070	malate dehydrogenase (oxaloacetate-decarboxylating)
360	stig_17	56281	56347	intron_retention	65.6821	178.1	Gs23850	hypothetical protein Gasu_23850
361	stig_17	130792	130848	intron_retention	175.883	153.143		
362	stig_17	28161	28213	intron_retention	123.576	146.608	Gs23710	glycerol-3-phosphate dehydrogenase (NAD(P)+) isoform2
363	stig_17	119324	119372	intron_retention	94.4309	90.9695	Gs24200	chorismate mutase
364	stig_17	146399	146450	intron_retention	81.5972	129.863	Gs24350	tRNA-intron endonuclease
365	stig_17	103420	103472	intron_retention	471.739	209.922	Gs24100	peroxisomal membrane protein-related protein
366	stig_17	56398	56459	intron_retention	44.5326	134.364		
367	stig_17	179815	179870	intron_retention	139.024	82.6707	Gs24510	hypothetical protein isoform 2
368	stig_17	169406	169474	intron_retention	249.952	339.57	Gs24440	riboflavin synthase alpha chain
369	stig_17	128619	128671	intron_retention	200.051	155.382	Gs24250	exodeoxyribonuclease III
370	stig_17	42012	42079	intron_retention	641.359	544.776		
371	stig_170	702	771	intron_retention	106.99	67.3918	Gs63370	elongation factor EF-2
372	stig_173	2284	2334	intron_retention	236.525	325.169	Gs63430	hypothetical protein isoform 1
373	stig_173	2456	2511	intron_retention	173.268	224.525	Gs63430	hypothetical protein isoform 1
374	stig_173	2756	2816	intron_retention	170.957	268.302		
375	stig_173	3602	3652	intron_retention	112.583	240.911	Gs63440	hypothetical protein Gasu_63440
376	stig_175	993	1050	intron_retention	17.2628	47.3153		
377	stig_178	2909	2972	intron_retention	1294.03	1116.44	Gs63520	hypothetical protein Gasu_63520
378	stig_18	17734	17781	intron_retention	90.9588	80.734		
379	stig_18	82437	82489	intron_retention	558.448	757.898	Gs25040	hypothetical protein Gasu_25040

380	stig_18	139893	139942	intron_retention	136.476	114.23	Gs25390	hypothetical protein Gasu_25390
381	stig_18	120983	121040	intron_retention	288.037	299.559	Gs25300	glycerophosphodiester phosphodiesterase
382	stig_18	33502	33557	intron_retention	327.286	250.983	Gs24760	zinc finger protein
383	stig_18	34050	34127	intron_retention	779.899	558.218	Gs24760	zinc finger protein
384	stig_18	160534	160586	intron_retention	74.1005	120.953	Gs25550	SOH1-like protein (ISS) isoform 2
385	stig_18	141629	141688	intron_retention	95.7286	100.475	Gs25410	hypothetical protein isoform 2
386	stig_18	118797	118846	intron_retention	5174.9	257.887	Gs25270	phosphatidate cytidylyltransferase/ phytol kinase
387	stig_18	38992	39047	intron_retention	40.4926	109.677	Gs24800	hypothetical protein Gasu_24800
388	stig_18	133968	134042	intron_retention	472.649	386.839	Gs25370	hypothetical protein Gasu_25370
389	stig_18	134215	134266	intron_retention	603.521	534.026	Gs25370	hypothetical protein Gasu_25370
390	stig_18	160422	160469	intron_retention	167.659	255.455	Gs25550	SOH1-like protein (ISS) isoform 2
391	stig_18	92729	92903	intron_retention	202.833	135.176		
392	stig_18	67782	67830	intron_retention	206.583	227.197	Gs24960	transducin family protein / WD-40 repeat family protein
393	stig_18	93568	93625	intron_retention	367.061	351.798	Gs25100	translation initiation factor
394	stig_18	97615	97666	intron_retention	60.622	50.3582	Gs25130	3-hydroxybutyryl-CoA dehydrogenase
395	stig_18	34183	34230	intron_retention	600.582	452.084	Gs24760	zinc finger protein
396	stig_18	146576	146650	intron_retention	63.6814	45.9827	Gs25450	hypothetical protein Gasu_25450
397	stig_18	39310	39373	intron_retention	57.5653	174.832	Gs24800	hypothetical protein Gasu_24800
398	stig_18	68112	68161	intron_retention	98.1331	124.84	Gs24960	transducin family protein / WD-40 repeat family protein
399	stig_18	100815	100866	intron_retention	243.933	398.788	Gs25160	hypothetical protein Gasu_25160
400	stig_18	141874	141936	intron_retention	95.4671	110.266	Gs25410	hypothetical protein isoform 2
401	stig_18	90715	90790	intron_retention	65.1606	76.0221		
402	stig_18	142193	142257	intron_retention	463.93	285.928		
403	stig_18	48682	48729	intron_retention	630.953	1195.18	Gs24850	short-chain dehydrogenase/reductase SDR isoform 2
404	stig_18	39512	39573	intron_retention	97.4047	260.397	Gs24800	hypothetical protein Gasu_24800
405	stig_18	148980	149041	intron_retention	1689.33	856.344	Gs25470	myo-inositol 2-dehydrogenase
406	stig_18	78035	78089	intron_retention	81.5185	99.3344	Gs25010	aminomethyltransferase
407	stig_18	78218	78282	intron_retention	51.4252	84.2685	Gs25010	aminomethyltransferase
408	stig_18	48049	48096	intron_retention	194.916	446.304	Gs24850	short-chain dehydrogenase/reductase SDR isoform 2
409	stig_18	111262	111341	intron_retention	47.8502	75.3697		
410	stig_18	90372	90554	intron_retention	134.202	80.8133	Gs25080	kinesin family member isoform 1
411	stig_18	39768	39820	intron_retention	207.143	318.256	Gs24800	hypothetical protein Gasu_24800
412	stig_18	48336	48389	intron_retention	339.769	772.777	Gs24850	short-chain dehydrogenase/reductase SDR isoform 2
413	stig_18	47847	47903	intron_retention	128.107	251.979	Gs24850	short-chain dehydrogenase/reductase SDR isoform 2
414	stig_18	48473	48527	intron_retention	462.748	1052.37	Gs24850	short-chain dehydrogenase/reductase SDR isoform 2
415	stig_18	48168	48230	intron_retention	253.731	629.147	Gs24850	short-chain dehydrogenase/reductase SDR isoform 2
416	stig_19	163398	163449	intron_retention	64.3193	56.7491	Gs26550	transcription initiation factor TFIIB
417	stig_19	90580	90633	intron_retention	284.776	348.675	Gs26110	hypothetical protein Gasu_26110
418	stig_19	172616	172688	intron_retention	90.2007	136.999	Gs26580	hypothetical protein Gasu_26580
419	stig_19	174389	174444	intron_retention	59.7481	68.6996	Gs26600	tenascin isoform 1
420	stig_19	89974	90024	intron_retention	87.0686	121.012	Gs26110	hypothetical protein Gasu_26110
421	stig_19	84156	84211	intron_retention	54.7474	64.5067	Gs26080	phosphoribosylformimino-5-aminoimidazole carboxamide ribotideisomerase
422	stig_19	174822	174873	intron_retention	44.3089	65.1638	Gs26600	tenascin isoform 1

423	stig_19	66677	66725	intron_retention	25.4893	31.0458	Gs25990	AAA-type ATPase isoform 2
424	stig_19	174288	174337	intron_retention	72.0429	77.6885	Gs26600	tenascin isoform 1
425	stig_19	174691	174737	intron_retention	39.0909	62.7146	Gs26600	tenascin isoform 1
426	stig_19	176207	176264	intron_retention	46.8432	58.4451	Gs26610	hypothetical protein Gasu_26610
427	stig_19	160420	160572	intron_retention	148.868	206.95	Gs26520	protein transport protein Sec24-like protein isoform 1
428	stig_19	96717	96765	intron_retention	318.841	310.097	Gs26150	transcription elongation factor SPT4-like protein
429	stig_19	175108	175161	intron_retention	50.0866	73.2059	Gs26600	tenascin isoform 1
430	stig_19	174603	174651	intron_retention	43.2676	67.408	Gs26600	tenascin isoform 1
431	stig_19	172739	172786	intron_retention	113.285	156.552	Gs26580	hypothetical protein Gasu_26580
432	stig_19	90270	90314	intron_retention	188.479	219.325	Gs26110	hypothetical protein Gasu_26110
433	stig_19	124741	124787	intron_retention	86.1043	104.597		
434	stig_19	154898	154945	intron_retention	336.263	266.546	Gs26500	DNA mismatch repair protein MutS isoform 1
435	stig_19	91007	91057	intron_retention	327.4	285.85	Gs26110	hypothetical protein Gasu_26110
436	stig_19	163217	163270	intron_retention	43.8366	39.1115	Gs26550	transcription initiation factor TFIIB
437	stig_19	138950	139004	intron_retention	250.16	306.183	Gs26400	coiled-coil-helix-coiled-coil-helix domain-containing protein 5
438	stig_19	21806	21952	intron_retention	188.442	51.5403	Gs25760	omega-6 fatty acid desaturase (delta-12 desaturase)
439	stig_19	2330	2395	intron_retention	167.251	113.713		
440	stig_19	89275	89333	intron_retention	183.507	85.1111		
441	stig_19	34722	34900	intron_retention	176.608	67.3753		
442	stig_2	194277	194334	intron_retention	167.743	174.115	Gs04900	hypothetical protein Gasu_04900
443	stig_2	117206	117253	intron_retention	114.261	96.4198	Gs04460	peptidylprolyl isomerase
444	stig_2	157243	157295	intron_retention	104.102	57.5903	Gs04630	hypothetical protein isoform 2
445	stig_2	52996	53051	intron_retention	46.7493	49.2462		
446	stig_2	289902	289952	intron_retention	1166.76	995.016	Gs05340	hypothetical protein isoform 1
447	stig_2	34769	34824	intron_retention	77.1312	87.7128	Gs04080	transducin family protein / WD-40 repeat family protein
448	stig_2	132065	132113	intron_retention	95.3717	115.947		
449	stig_2	196946	196991	intron_retention	243.289	278.156	Gs04910	3-hydroxyisobutyrate dehydrogenase
450	stig_2	163316	163363	intron_retention	628.862	716.484	Gs04670	hypothetical protein Gasu_04670
451	stig_2	163561	163618	intron_retention	811.038	773.656	Gs04670	hypothetical protein Gasu_04670
452	stig_2	163221	163274	intron_retention	465.417	564.078	Gs04670	hypothetical protein Gasu_04670
453	stig_2	293951	293999	intron_retention	207.446	74.5024	Gs05370	adenylate kinase
454	stig_2	163427	163474	intron_retention	938.546	970.419	Gs04670	hypothetical protein Gasu_04670
455	stig_2	73422	73470	intron_retention	102.752	60.1033	Gs04270	26S proteasome regulatory subunit N8
456	stig_2	167919	167972	intron_retention	213.729	159.618	Gs04710	tRNA modification GTPase isoform 1
457	stig_2	164192	164244	intron_retention	124.853	102.572	Gs04680	GATA transcription factor areB gamma-like protein
458	stig_2	209695	209745	intron_retention	60.1696	133.601	Gs04950	zinc finger CCCH-type containing 3 isoform 2
459	stig_2	257668	257728	intron_retention	282.246	167.503		
460	stig_2	293832	293882	intron_retention	282.798	97.8912	Gs05370	adenylate kinase
461	stig_2	268698	268748	intron_retention	129.303	152.5	Gs05240	early-responsive to dehydration protein-related protein
462	stig_2	100151	100193	intron_retention	245.946	515.489		
463	stig_2	50514	50565	intron_retention	173.82	180.027	Gs04150	beta-amylase isoform 2
464	stig_2	66206	66259	intron_retention	980.313	722.356	Gs04220	nuclear transport factor, putative
465	stig_2	187446	187488	intron_retention	62.0242	115.137	Gs04850	PEBP family protein

466	stig_2	269807	269852	intron_retention	241.301	96.1624	Gs05250	hypothetical protein Gasu_05250
467	stig_2	65920	65994	intron_retention	90.803	52.1246	Gs04220	nuclear transport factor, putative
468	stig_2	187555	187615	intron_retention	68.0544	151.984	Gs04850	PEBP family protein
469	stig_2	162652	162703	intron_retention	176.661	280.278	Gs04660	glucosamine--fructose-6-phosphate aminotransferase (isomerizing)isoform 1
470	stig_2	132435	132479	intron_retention	68.9495	104.448	Gs04490	hypothetical protein Gasu_04490
471	stig_2	81276	81332	intron_retention	194.675	139.217	Gs04310	hypothetical protein Gasu_04310
472	stig_2	197198	197248	intron_retention	169.282	194.235	Gs04910	3-hydroxyisobutyrate dehydrogenase
473	stig_2	158542	158590	intron_retention	576.168	660.704	Gs04640	ATP binding / protein kinase
474	stig_2	190833	190882	intron_retention	261.717	389.654	Gs04880	hypothetical protein isoform 2
475	stig_2	225601	225652	intron_retention	111.509	131.918	Gs05050	multidrug resistance protein, MOP family
476	stig_2	61002	61058	intron_retention	41.7731	135.106		
477	stig_2	162352	162408	intron_retention	117.728	198.219	Gs04660	glucosamine--fructose-6-phosphate aminotransferase (isomerizing)isoform 1
478	stig_2	255760	255813	intron_retention	330.629	271.318		
479	stig_2	162487	162546	intron_retention	141.697	238.612	Gs04660	glucosamine--fructose-6-phosphate aminotransferase (isomerizing)isoform 1
480	stig_2	222533	222580	intron_retention	206.127	202.878	Gs05020	hypothetical protein Gasu_05020
481	stig_2	115157	115210	intron_retention	116.603	173.988	Gs04450	D-lactate dehydrogenase (cytochrome)
482	stig_2	225802	225848	intron_retention	108.954	116.31	Gs05050	multidrug resistance protein, MOP family
483	stig_2	99949	100019	intron_retention	230.409	509.921		
484	stig_2	215331	215382	intron_retention	229.42	283.923		
485	stig_2	193767	193820	intron_retention	119.833	116.433	Gs04890	vacuolar assembly protein Vps41
486	stig_2	100337	100385	intron_retention	303.483	469.169		
487	stig_2	136766	136816	intron_retention	431.149	265.361	Gs04520	hypothetical protein Gasu_04520
488	stig_20	58665	58713	intron_retention	164.41	201.679	Gs26830	hypothetical protein Gasu_26830
489	stig_20	64375	64427	intron_retention	78.9926	218.093	Gs26870	pyrophosphatase, MutT/nudix family protein
490	stig_20	93213	93265	intron_retention	267.8	505.604	Gs27010	SEC-C motif domain protein
491	stig_20	160687	160740	intron_retention	79.572	93.7567		
492	stig_20	92889	92940	intron_retention	134.706	262.194	Gs27010	SEC-C motif domain protein
493	stig_20	93336	93391	intron_retention	203.057	419.555	Gs27010	SEC-C motif domain protein
494	stig_20	180057	180299	intron_retention	60.4823	68.4596		
495	stig_20	118585	118641	intron_retention	87.402	72.5092	Gs27180	S-adenosylmethionine:tRNA ribosyltransferase-isomerase
496	stig_20	33982	34030	intron_retention	69.5029	93.6786	Gs26670	translin family protein isoform 1
497	stig_20	64207	64253	intron_retention	106.063	264.874	Gs26870	pyrophosphatase, MutT/nudix family protein
498	stig_20	29511	29582	intron_retention	50.2535	63.9136		
499	stig_20	188794	188847	intron_retention	138.32	130.723	Gs27550	GTP-binding protein isoform 2
500	stig_20	83226	83279	intron_retention	142.102	199.76	Gs26960	chalcone synthase
501	stig_20	58062	58225	intron_retention	112.112	182.474		
502	stig_20	64036	64090	intron_retention	65.8909	148.42		
503	stig_20	167264	167315	intron_retention	169.458	140.572		
504	stig_20	59139	59186	intron_retention	92.4556	89.2327	Gs26830	hypothetical protein Gasu_26830
505	stig_20	38230	38281	intron_retention	39.4875	42.115	Gs26690	DNA repair and recombination protein RAD52
506	stig_20	156437	156486	intron_retention	166.225	158.67	Gs27400	hypothetical protein Gasu_27400

507	stig_21	101178	101233	intron_retention	98.5703	79.8176		
508	stig_21	138073	138120	intron_retention	165.151	191.056	Gs28410	4-diphosphocytidyl-2-C-methyl-D-erythritol kinase
509	stig_21	9383	9438	intron_retention	80.775	87.9075	Gs27670	transducin family protein / WD-40 repeat family protein
510	stig_21	140545	140600	intron_retention	130.613	144.781	Gs28420	hypothetical protein isoform 1
511	stig_21	140919	140965	intron_retention	58.115	27.7917	Gs28420	hypothetical protein isoform 1
512	stig_21	151328	151385	intron_retention	324.522	324.339	Gs28490	beta-1,3-N-acetylglucosaminyltransferase 5
513	stig_21	164954	165002	intron_retention	158.328	172.063	Gs28560	UDP-N-acetylglucosamine--dolichyl-phosphateN-acetylglucosaminephosphotransferase
514	stig_21	10355	10411	intron_retention	185.782	97.9723	Gs27680	D-tyrosyl-tRNA(Tyr) deacylase
515	stig_21	122271	122318	intron_retention	293.313	229.851	Gs28310	6-pyruvoyl tetrahydropterin synthase
516	stig_21	19885	19938	intron_retention	76.0164	84.1358	Gs27740	phosphatase isoform 1
517	stig_21	16840	16895	intron_retention	194.673	153.136	Gs27720	transducin family protein / WD-40 repeat family protein
518	stig_21	110125	110187	intron_retention	51.4325	48.2614		
519	stig_21	124304	124357	intron_retention	47.9315	52.3828	Gs28320	transducin family protein / WD-40 repeat family protein
520	stig_21	150588	150644	intron_retention	105.586	96.6493	Gs28480	hypothetical protein Gasu_28480
521	stig_21	122371	122422	intron_retention	314.473	246.938	Gs28310	6-pyruvoyl tetrahydropterin synthase
522	stig_21	100803	100858	intron_retention	122.109	179.798	Gs28200	metal ion (Mn2+/Co2+) transporter, MIT family isoform 2
523	stig_21	168916	168965	intron_retention	124.737	164.714	Gs28600	EF-1-alpha-related GTP-binding protein
524	stig_21	110260	110320	intron_retention	46.899	43.9957		
525	stig_21	122184	122235	intron_retention	358.282	257.286	Gs28310	6-pyruvoyl tetrahydropterin synthase
526	stig_21	23549	23598	intron_retention	139.26	187.978	Gs27760	sugar-phosphate:phosphate translocator, DMT family isoform 2
527	stig_21	9930	9981	intron_retention	191.623	139.583	Gs27680	D-tyrosyl-tRNA(Tyr) deacylase
528	stig_21	51241	51291	intron_retention	392.652	186.64	Gs27950	dUTP pyrophosphatase
529	stig_21	175473	175528	intron_retention	795.805	367.268	Gs28640	small nuclear ribonucleoprotein F isoform 2
530	stig_21	167799	167850	intron_retention	33.9962	130.539	Gs28590	glutathione S-transferase isoform 1
531	stig_22	20544	20593	intron_retention	251.249	118.247	Gs28830	UDP-glucose/GDP-mannose dehydrogenase isoform 1
532	stig_22	99383	99450	intron_retention	203.551	579.14	Gs29280*	MFS transporter
533	stig_22	91883	91936	intron_retention	126.347	105.514	Gs29220	flap endonuclease-1
534	stig_22	47332	47380	intron_retention	69.53	66.8987	Gs28980	glutathione S-transferase
535	stig_22	26846	26900	intron_retention	196.248	257.644	Gs28870	hypothetical protein Gasu_28870
536	stig_22	107574	107624	intron_retention	178.095	227.363	Gs29340	alpha,alpha-trehalase
537	stig_22	74752	74802	intron_retention	198.769	111.352	Gs29140	RNA-binding protein
538	stig_22	47113	47164	intron_retention	105.892	90.8643	Gs28980	glutathione S-transferase
539	stig_22	19704	19763	intron_retention	273.478	271.829	Gs28830	UDP-glucose/GDP-mannose dehydrogenase isoform 1
540	stig_22	81904	81957	intron_retention	242.661	301.876		
541	stig_22	120037	120084	intron_retention	62.1548	77.7936		
542	stig_22	151049	151109	intron_retention	98.9071	52.1833	Gs29600	UNC-50 family protein isoform 1
543	stig_22	18503	18554	intron_retention	284.974	191.943	Gs28810	ADP-ribosylation factor
544	stig_22	17659	17712	intron_retention	174.857	113.546	Gs28800	BET3 vesicular transport protein
545	stig_22	29425	29479	intron_retention	143.355	77.9327	Gs28900	malate dehydrogenase
546	stig_22	131149	131209	intron_retention	72.6412	135.518	Gs29480	heat shock transcription
547	stig_22	22286	22334	intron_retention	190.573	171.845	Gs28840	phosphopantothenate--cysteine ligase
548	stig_22	151312	151372	intron_retention	195.415	115.596	Gs29600	UNC-50 family protein isoform 1

549	stig_22	80399	80458	intron_retention	209.484	165.657	Gs29190	hydrolase
550	stig_22	157320	157371	intron_retention	241.519	1500.34	Gs29650	hypothetical protein Gasu_29650
551	stig_22	26490	26559	intron_retention	94.6824	261.755	Gs28870	hypothetical protein Gasu_28870
552	stig_22	153248	153323	intron_retention	485.331	645.949		
553	stig_22	151415	151470	intron_retention	487.974	351.138	Gs29600	UNC-50 family protein isoform 1
554	stig_22	151229	151282	intron_retention	131.604	89.8776	Gs29600	UNC-50 family protein isoform 1
555	stig_22	124156	124210	intron_retention	58.2803	207.016	Gs29430	ABC transporter, ATP-binding & transmembrane domain
556	stig_22	74883	74937	intron_retention	261.924	262.651	Gs29140	RNA-binding protein
557	stig_22	95203	95279	intron_retention	485.307	346.738	Gs29240	dihydrodipicolinate synthase
558	stig_221	4461	4520	intron_retention	33.5816	47.8242		
559	stig_229	378	443	intron_retention	45.583	44.1945		
560	stig_23	123578	123619	intron_retention	44.6602	64.6218	Gs30470	endonuclease III isoform 2
561	stig_23	85910	85974	intron_retention	48.8375	88.7443		
562	stig_23	114629	114679	intron_retention	55.8237	77.3394	Gs30410	hypothetical protein Gasu_30410
563	stig_23	87750	87801	intron_retention	164.93	236.228	Gs30250	26S proteasome non-ATPase regulatory subunit 9
564	stig_23	36632	36677	intron_retention	133.48	190.385	Gs29950	poly(A) binding protein
565	stig_23	87543	87589	intron_retention	324.177	443.934	Gs30250	26S proteasome non-ATPase regulatory subunit 9
566	stig_23	124119	124182	intron_retention	57.7198	108.709		
567	stig_23	166553	166613	intron_retention	133.392	167.167	Gs30760	DNA repair exonuclease
568	stig_23	42338	42385	intron_retention	100.785	34.0541	Gs29980	phosphoribosylglycinamide formyltransferase
569	stig_23	6349	6396	intron_retention	223.364	184.23	Gs29800	ABC transporter, ATP-binding protein
570	stig_23	73810	73883	intron_retention	260.872	240.952	Gs30160	longevity assurance protein LAG1
571	stig_23	72796	72841	intron_retention	222.96	207.79	Gs30150	hypothetical protein Gasu_30150
572	stig_23	36522	36577	intron_retention	242.717	294.929	Gs29950	poly(A) binding protein
573	stig_23	144734	144781	intron_retention	143.987	282.32	Gs30610	alpha-1,4-N-acetylglucosaminyltransferase EXTL3
574	stig_23	4645	4693	intron_retention	157.537	106.833	Gs29780	NADPH2:quinone reductase
575	stig_23	123771	123821	intron_retention	132.337	189.116	Gs30470	endonuclease III isoform 2
576	stig_23	111640	111696	intron_retention	78.9563	165.04		
577	stig_23	114759	114812	intron_retention	32.8382	51.9958	Gs30410	hypothetical protein Gasu_30410
578	stig_23	33285	33339	intron_retention	87.0337	97.1599		
579	stig_23	43310	43432	intron_retention	660.077	1099.13	Gs29990	periplasmic divalent cation tolerance protein isoform 1
580	stig_23	37220	37282	intron_retention	56.8966	253.418		
581	stig_23	50195	50247	intron_retention	420.068	602.586	Gs30030	guanylate kinase
582	stig_23	114456	114506	intron_retention	680.112	587.565		
583	stig_23	111833	111903	intron_retention	138.07	223.02	Gs30390	hypothetical protein Gasu_30390
584	stig_23	167017	167068	intron_retention	111.941	137.045	Gs30760	DNA repair exonuclease
585	stig_23	123492	123547	intron_retention	247.359	257.597	Gs30470	endonuclease III isoform 2
586	stig_23	128079	128131	intron_retention	284.292	166.14		
587	stig_23	124464	124521	intron_retention	47.6069	82.6011		
588	stig_23	133310	133363	intron_retention	813.893	140.649	Gs30520	hypothetical protein Gasu_30520
589	stig_23	43773	43824	intron_retention	438.601	570.637	Gs29990	periplasmic divalent cation tolerance protein isoform 1
590	stig_233	2856	2905	intron_retention	44.2983	82.1414	Gs64200	dTDP-4-dehydrorhamnose reductase
591	stig_24	165539	165589	intron_retention	117.415	238.466	Gs31630	arsenite methyltransferase
592	stig_24	107799	107871	intron_retention	328.396	267.554	Gs31290	signal recognition particle subunit SRP19 isoform 2

593	stig_24	121110	121155	intron_retention	82.1751	112.571	Gs31390	dephospho-CoA kinase
594	stig_24	107656	107704	intron_retention	184.026	175.876	Gs31290	signal recognition particle subunit SRP19 isoform 2
595	stig_24	43354	43410	intron_retention	480.004	297.126	Gs31030	histone chaperone ASF1
596	stig_24	12591	12661	intron_retention	152.374	138.015	Gs30850	thioredoxin-like protein
597	stig_24	23606	23657	intron_retention	145.902	157.685		
598	stig_24	13659	13713	intron_retention	88.9476	85.9103	Gs30860	hypothetical protein Gasu_30860
599	stig_24	88241	88289	intron_retention	79.0082	93.6906	Gs31220*	3-deoxy-manno-octulosonate cytidylyltransferase (CMP-KDOsynthetase)
600	stig_24	162330	162379	intron_retention	66.9981	107.151	Gs31610	hypothetical protein Gasu_31610
601	stig_24	144138	144190	intron_retention	113.916	88.1055	Gs31510	band 7 family protein isoform 2
602	stig_24	164252	164326	intron_retention	50.2962	93.4483		
603	stig_24	108866	108918	intron_retention	164.875	201.121	Gs31300	hypothetical protein Gasu_31300
604	stig_24	10962	11010	intron_retention	138.937	210.408	Gs30840	mRNA binding / poly(U) binding protein
605	stig_24	32071	32133	intron_retention	322.387	779.549	Gs30960	hypothetical protein Gasu_30960
606	stig_24	9668	9713	intron_retention	204.478	256.431	Gs30830	shikimate kinase
607	stig_24	62840	62910	intron_retention	62.4134	92.8599		
608	stig_24	115821	115873	intron_retention	204.451	131.834	Gs31350	hypothetical protein Gasu_31350
609	stig_24	19451	19504	intron_retention	81.7516	100.006	Gs30900	hypothetical protein isoform 2
610	stig_24	107974	108027	intron_retention	74.4257	58.1999	Gs31290	signal recognition particle subunit SRP19 isoform 2
611	stig_24	158934	158983	intron_retention	90.2988	140.737	Gs31590	protein geranylgeranyltransferase type II
612	stig_24	164431	164487	intron_retention	77.6248	173.277		
613	stig_24	143708	143761	intron_retention	230.292	116.369	Gs31510	band 7 family protein isoform 2
614	stig_24	18094	18147	intron_retention	23.7656	55.3826	Gs30890	haloacid dehalogenase-like hydrolase family protein
615	stig_24	143839	143890	intron_retention	196.827	99.4846	Gs31510	band 7 family protein isoform 2
616	stig_24	144015	144070	intron_retention	157	85.073	Gs31510	band 7 family protein isoform 2
617	stig_24	19057	19106	intron_retention	134.328	164.743	Gs30900	hypothetical protein isoform 2
618	stig_24	97669	97719	intron_retention	64.6737	59.8837	Gs31270	DNA excision repair protein ERCC-2
619	stig_24	13193	13242	intron_retention	192.721	158.495		
620	stig_24	160234	160324	intron_retention	148.853	194.879		
621	stig_241	1078	1131	intron_retention	75.0496	96.1266		
622	stig_243	3012	3072	intron_retention	133.364	123.836		
623	stig_25	40676	40732	intron_retention	130.167	85.2783	Gs31930	hypothetical protein Gasu_31930
624	stig_25	91508	91576	intron_retention	49.4172	75.8181	Gs32180	hypothetical protein Gasu_32180
625	stig_25	60768	60826	intron_retention	57.9174	92.3616	Gs32000	peptidylprolyl isomerase
626	stig_25	15154	15215	intron_retention	184.601	207.962	Gs31750	cell cycle arrest protein BUB2
627	stig_25	127519	127568	intron_retention	343.837	399.834	Gs32320	26S proteasome regulatory subunit N12
628	stig_25	83837	83893	intron_retention	118.119	204.056	Gs32140	hypothetical protein isoform 2
629	stig_25	162057	162149	intron_retention	59.8142	93.3219	Gs32530	hypothetical protein isoform 1
630	stig_25	103954	104002	intron_retention	167.813	197.002	Gs32250	myo-inositol dehydrogenase
631	stig_25	86029	86101	intron_retention	129.536	115.201	Gs32150	cytosolic nonspecific dipeptidase
632	stig_25	112912	112969	intron_retention	183.277	195.543	Gs32290	translation initiation factor eIF-2B epsilon subunit isoform 2
633	stig_25	13047	13100	intron_retention	179.035	201.764	Gs31720	vacuolar iron transporter 1 isoform 1
634	stig_25	84039	84084	intron_retention	54.8181	107.087	Gs32140	hypothetical protein isoform 2

635	stig_25	151276	151328	intron_retention	315.771	308.913	Gs32470	aldo/keto reductase
636	stig_25	74460	74513	intron_retention	874.221	795.85	Gs32080	hypothetical protein Gasu_32080
637	stig_25	67027	67085	intron_retention	206.854	499.62	Gs32050	zinc finger protein
638	stig_25	86478	86532	intron_retention	109.726	199.125	Gs32160	V-type H+-transporting ATPase subunit c isoform 2
639	stig_25	90384	90443	intron_retention	320.946	456.198	Gs32170	transducin family protein / WD-40 repeat family protein
640	stig_25	60898	60953	intron_retention	43.2659	60.6082	Gs32000	peptidylprolyl isomerase
641	stig_25	32162	32222	intron_retention	66.5076	32.6177	Gs31850	hypothetical protein Gasu_31850
642	stig_25	110906	110962	intron_retention	117.69	102.448	Gs32270	haloacid dehalogenase-like hydrolase family protein
643	stig_25	127343	127400	intron_retention	208.721	245.992	Gs32320	26S proteasome regulatory subunit N12
644	stig_25	143983	144026	intron_retention	39.6329	65.2118		
645	stig_25	65570	65622	intron_retention	151.726	373.047		
646	stig_25	83218	83280	intron_retention	766.083	787.233	Gs32140	hypothetical protein isoform 2
647	stig_25	34410	34462	intron_retention	291.486	449.933	Gs31870	chaperone protein / DnaJ-related protein
648	stig_25	140357	140416	intron_retention	108.903	76.3944	Gs32400	glycolipid binding / glycolipid transporter
649	stig_25	37344	37406	intron_retention	198.312	146.626	Gs31890	auxin efflux carrier
650	stig_25	138935	138999	intron_retention	469.547	477.019	Gs32390	glutaminyl-tRNA synthetase
651	stig_25	111805	111864	intron_retention	249.511	414.267	Gs32280	hypothetical protein isoform 2
652	stig_25	58890	58954	intron_retention	247.114	194.961	Gs31990	tyrosyl-tRNA synthetase
653	stig_25	25164	25214	intron_retention	839.508	664.407		
654	stig_25	86289	86356	intron_retention	100.357	157.785	Gs32160	V-type H+-transporting ATPase subunit c isoform 2
655	stig_25	159484	159539	intron_retention	109.561	181.679	Gs32520	hypothetical protein Gasu_32520
656	stig_25	151787	151848	intron_retention	159.33	213.37	Gs32480	Mg2+ uptake permease (NIPA), DMT family
657	stig_25	74071	74133	intron_retention	176.932	171.718	Gs32070	Smad nuclear interacting protein 1 isoform 1
658	stig_25	112526	112581	intron_retention	1262.49	1845.52		
659	stig_25	34834	34906	intron_retention	331.779	405.418		
660	stig_254	3575	3652	intron_retention	122.241	161.576	Gs64520	hypothetical protein Gasu_64520
661	stig_26	122475	122535	intron_retention	61.4137	66.3405	Gs33170	hypothetical protein Gasu_33170
662	stig_26	160115	160176	intron_retention	93.3536	153.877	Gs33350	hypothetical protein Gasu_33350
663	stig_26	160840	160897	intron_retention	92.8593	85.8281	Gs33360	hypothetical protein Gasu_33360
664	stig_26	80750	80809	intron_retention	91.517	99.0259		
665	stig_26	64474	64517	intron_retention	278.046	244.274	Gs32910	tRNA pseudouridine synthase B isoform 1
666	stig_26	122887	122936	intron_retention	223.224	119.156	Gs33170	hypothetical protein Gasu_33170
667	stig_26	48520	48575	intron_retention	230.043	232.214	Gs32820	programmed cell death 8 (apoptosis-inducing factor)
668	stig_26	86951	87003	intron_retention	139.325	147.016	Gs33030	hypothetical protein Gasu_33030
669	stig_26	64279	64328	intron_retention	376.541	305.098	Gs32910	tRNA pseudouridine synthase B isoform 1
670	stig_26	64040	64088	intron_retention	439.41	333.736	Gs32910	tRNA pseudouridine synthase B isoform 1
671	stig_26	122662	122714	intron_retention	78.2509	69.2724	Gs33170	hypothetical protein Gasu_33170
672	stig_26	92598	92770	intron_retention	64.9649	73.2398		
673	stig_26	166695	166755	intron_retention	96.0451	54.4344		
674	stig_26	33315	33360	intron_retention	365.64	418.449	Gs32760	polyphosphate kinase isoform 1
675	stig_26	141681	141735	intron_retention	136.717	187.29	Gs33270	magnesium-dependent phosphatase 1
676	stig_26	32048	32101	intron_retention	64.2879	66.4336		
677	stig_26	151209	151255	intron_retention	112.108	98.6794	Gs33320	1-phosphatidylinositol-4-phosphate 5-kinase
678	stig_26	83216	83271	intron_retention	88.3553	195.898		

679	stig_26	48659	48729	intron_retention	245.772	236.732	Gs32820	programmed cell death 8 (apoptosis-inducing factor)
680	stig_26	141436	141485	intron_retention	95.2143	129.15	Gs33270	magnesium-dependent phosphatase 1
681	stig_26	91165	91231	intron_retention	71.2699	91.6837	Gs33050	hypothetical protein Gasu_33050
682	stig_26	63565	63621	intron_retention	283.977	322.112	Gs32900	hypothetical protein Gasu_32900
683	stig_26	80946	80998	intron_retention	61.7929	104.083	Gs32990	cytochrome c biogenesis protein, putative, Ccb2
684	stig_26	71568	71615	intron_retention	345.217	346.669	Gs32960	4-carboxymuconolactone decarboxylase
685	stig_26	47118	47177	intron_retention	306.815	314.919	Gs32810	short chain dehydrogenase
686	stig_26	141875	141927	intron_retention	146.742	190.417	Gs33270	magnesium-dependent phosphatase 1
687	stig_26	92387	92538	intron_retention	42.1042	61.9064		
688	stig_26	13295	13352	intron_retention	129.759	136.034	Gs32610	hypothetical protein isoform 1
689	stig_26	125464	125522	intron_retention	308.281	171.137	Gs33190	hypothetical protein Gasu_33190
690	stig_26	97325	97375	intron_retention	30.2891	60.2149	Gs33070	MFS transporter, SP family, sugar:H+ symporter
691	stig_266	3416	3484	intron_retention	106.707	102.776		
692	stig_27	152515	152561	intron_retention	240.285	112.182	Gs34230	ATP-dependent RNA helicase
693	stig_27	53796	53845	intron_retention	261.912	258.584		
694	stig_27	33417	33464	intron_retention	288.443	305.446	Gs33550	hypothetical protein Gasu_33550
695	stig_27	8618	8676	intron_retention	156.471	204.836	Gs33460	heparan-alpha-glucosaminide N-acetyltransferase isoform 2
696	stig_27	94128	94174	intron_retention	181.39	183.555	Gs33880	heme-binding protein
697	stig_27	93990	94042	intron_retention	209.337	242.355	Gs33880	heme-binding protein
698	stig_27	145976	146026	intron_retention	53.4042	112.901	Gs34180	hypothetical protein isoform 1
699	stig_27	57754	57798	intron_retention	271.163	308.143	Gs33700	pyrrolidone-carboxylate peptidase family protein
700	stig_27	146200	146250	intron_retention	28.2234	65.0929	Gs34180	hypothetical protein isoform 1
701	stig_27	108413	108471	intron_retention	48.0447	45.196	Gs33950	hypothetical protein Gasu_33950
702	stig_27	167448	167518	intron_retention	60.3212	61.15		
703	stig_27	61569	61616	intron_retention	194.814	159.55	Gs33730	pyrroline-5-carboxylate reductase
704	stig_27	145880	145929	intron_retention	69.3098	121.233	Gs34180	hypothetical protein isoform 1
705	stig_27	10920	10975	intron_retention	242.85	165.789	Gs33470	thiamine-phosphate pyrophosphorylase
706	stig_27	55539	55591	intron_retention	203.128	147.072	Gs33680	hypothetical protein Gasu_33680
707	stig_27	63628	63678	intron_retention	33.2438	29.5774	Gs33740	hypothetical protein Gasu_33740
708	stig_27	53652	53695	intron_retention	3252.51	5622.41		
709	stig_27	132299	132355	intron_retention	395.567	379.838		
710	stig_27	155062	155108	intron_retention	98.9105	118.411	Gs34240	hypothetical protein isoform 1
711	stig_27	87456	87513	intron_retention	11.6997	100.917		
712	stig_27	163224	163271	intron_retention	256.639	408.034	Gs34290	zinc-finger protein / transcription factor isoform 2
713	stig_27	13851	13904	intron_retention	106.637	72.1163	Gs33490	protein arginine N-methyltransferase 5 isoform 1
714	stig_27	163378	163460	intron_retention	205.843	308.258	Gs34290	zinc-finger protein / transcription factor isoform 2
715	stig_27	94205	94259	intron_retention	134.551	135.937	Gs33880	heme-binding protein
716	stig_27	75041	75087	intron_retention	260.546	298.301	Gs33800*	alpha-galactosidase
717	stig_27	164788	164841	intron_retention	131.047	50.4826	Gs34300	short-chain dehydrogenase/reductase (SDR) family protein
718	stig_27	33745	33797	intron_retention	339.581	456.739	Gs33550	hypothetical protein Gasu_33550
719	stig_27	164633	164688	intron_retention	109.3	54.1366	Gs34300	short-chain dehydrogenase/reductase (SDR) family protein

720	stig_27	74924	74976	intron_retention	343.627	385.397	Gs33800	alpha-galactosidase
721	stig_27	165010	165063	intron_retention	1249.55	127.471	Gs34300	short-chain dehydrogenase/reductase (SDR) family protein
722	stig_270	2444	2513	intron_retention	57.699	82.232	Gs64750	pyridoxamine 5-phosphate oxidase, putative
723	stig_270	2188	2238	intron_retention	101.442	141.261	Gs64750	pyridoxamine 5-phosphate oxidase, putative
724	stig_28	122052	122103	intron_retention	145.508	245.856		
725	stig_28	161012	161067	intron_retention	224.049	312.159		
726	stig_28	140830	140888	intron_retention	217.033	219.143	Gs34910	peptidase, M50 family protein
727	stig_28	160941	160988	intron_retention	91.9471	146.406	Gs35020	hypothetical protein isoform 2
728	stig_28	82181	82247	intron_retention	168.399	218.08	Gs34520	bifunctional quinolinate phosphoribosyltransferase /phosphoglycerate kinase
729	stig_28	112991	113053	intron_retention	176.804	163.055	Gs34720	hypothetical protein Gasu_34720
730	stig_28	117327	117380	intron_retention	126.519	112.442		
731	stig_28	99722	99781	intron_retention	147.975	140.485	Gs34620	hypothetical protein Gasu_34620
732	stig_28	174796	174852	intron_retention	160.747	165.585	Gs35090	UDP-sulfoquinovose synthase
733	stig_28	74962	75041	intron_retention	11.1445	120.975		
734	stig_28	148150	148211	intron_retention	184.347	201.245	Gs34950	hypothetical protein isoform 2
735	stig_28	108633	108690	intron_retention	438.317	186.442	Gs34680	mRNA (2'-O-methyladenosine-N6-)-methyltransferase
736	stig_28	158380	158427	intron_retention	211.268	153.643	Gs34990	hypothetical protein Gasu_34990
737	stig_28	86109	86160	intron_retention	165.489	131.249		
738	stig_28	112412	112461	intron_retention	418.548	402.439	Gs34710	hypothetical protein Gasu_34710
739	stig_28	90529	90583	intron_retention	225.738	312.195	Gs34560	hypothetical protein isoform 2
740	stig_28	145785	145855	intron_retention	146.624	166.926	Gs34930	acyl-activating enzyme 13
741	stig_28	13285	13331	intron_retention	507.315	658.51		
742	stig_28	173915	173966	intron_retention	433.593	449.512	Gs35090	UDP-sulfoquinovose synthase
743	stig_28	179012	179067	intron_retention	321.397	419.397		
744	stig_28	138524	138579	intron_retention	269.761	213.781	Gs34890	ubiquitin-conjugating enzyme E2
745	stig_28	194696	194758	intron_retention	34.9627	78.9549		
746	stig_29	70737	70789	intron_retention	235.47	490.086	Gs35610	hypothetical protein isoform 2
747	stig_29	70830	70883	intron_retention	80.3972	121.702	Gs35610	hypothetical protein isoform 2
748	stig_29	90300	90474	intron_retention	641.457	391.161		
749	stig_29	142504	142600	intron_retention	35.89	45.2452		
750	stig_29	82337	82392	intron_retention	95.3707	159.023		
751	stig_29	110797	110843	intron_retention	146.463	172.279	Gs35870	hypothetical protein Gasu_35870
752	stig_29	95789	95837	intron_retention	135.942	156.753	Gs35750	metal transporter, ACDP family isoform 1
753	stig_29	161077	161119	intron_retention	124.897	163.161	Gs36100	beta-1,4-N-acetylglucosaminyltransferase
754	stig_29	65719	65767	intron_retention	666.512	365.879	Gs35570	pre-mRNA branch site protein p14
755	stig_29	160716	160763	intron_retention	121.631	142.436	Gs36100	beta-1,4-N-acetylglucosaminyltransferase
756	stig_29	160512	160562	intron_retention	97.3348	98.1993	Gs36100	beta-1,4-N-acetylglucosaminyltransferase
757	stig_29	69994	70045	intron_retention	278.089	526.075		
758	stig_29	82662	82717	intron_retention	51.6818	114.853		
759	stig_29	82502	82551	intron_retention	79.8136	180.499		
760	stig_29	106076	106124	intron_retention	59.3988	137.377	Gs35830	histone acetyltransferase
761	stig_29	161303	161365	intron_retention	103.511	112.068		

762	stig_29	161208	161276	intron_retention	148.694	161.036		
763	stig_29	69699	69767	intron_retention	220.373	228.815	Gs35600	sulfate permease, SulP family
764	stig_29	95495	95548	intron_retention	283.409	318.692	Gs35750	metal transporter, ACDP family isoform 1
765	stig_29	67522	67573	intron_retention	237.126	279.73	Gs35590	hypothetical protein Gasu_35590
766	stig_29	138966	139019	intron_retention	120.255	39.9827	Gs35990	acid phosphatase
767	stig_29	107869	107918	intron_retention	191.717	160.823	Gs35840	N-carbamoyl-L-amino-acid hydrolase
768	stig_29	56871	56920	intron_retention	231.48	233.007		
769	stig_29	120137	120184	intron_retention	96.6461	86.3133	Gs35920	X-Pro dipeptidase isoform 2
770	stig_29	62472	62524	intron_retention	360.455	386.656	Gs35540	hypothetical protein isoform 2
771	stig_29	160819	160868	intron_retention	99.7337	124.742	Gs36100	beta-1,4-N-acetylglucosaminyltransferase
772	stig_29	86463	86512	intron_retention	417.966	419.442	Gs35680	DNA ligase 1
773	stig_29	69499	69543	intron_retention	115.01	111.136	Gs35600	sulfate permease, SulP family
774	stig_29	8011	8065	intron_retention	235.157	345.625	Gs35260	glucose repression regulatory protein TUP1
775	stig_29	40870	40925	intron_retention	222.358	117.911	Gs35420	RNA polymerase primary sigma factor
776	stig_29	62322	62385	intron_retention	341.373	396.827		
777	stig_29	61947	61997	intron_retention	611.334	505.515	Gs35530	ubiquitin-conjugating enzyme E2
778	stig_29	23766	23836	intron_retention	310.87	401.443		
779	stig_29	40985	41039	intron_retention	148.049	88.6162	Gs35420	RNA polymerase primary sigma factor
780	stig_29	14184	14234	intron_retention	1479.18	896.307	Gs35310	3-isopropylmalate dehydrogenase isoform 1
781	stig_29	62076	62128	intron_retention	512.494	479.941	Gs35530	ubiquitin-conjugating enzyme E2
782	stig_29	149789	149838	intron_retention	331.056	66.846	Gs36040	hypothetical protein isoform 2
783	stig_293	1798	1858	intron_retention	97.1832	81.9348		
784	stig_294	1865	1913	intron_retention	65.7549	129.728	Gs65100	putative translation factor
785	stig_294	1031	1085	intron_retention	343.467	493.042	Gs65100	putative translation factor
786	stig_299	258	309	intron_retention	154.119	170.84	Gs65160	hypothetical protein Gasu_65160, partial
787	stig_299	689	750	intron_retention	326.324	549.014		
788	stig_3	144997	145053	intron_retention	125.287	192.02	Gs06220	RNA-binding protein, putative
789	stig_3	170785	170836	intron_retention	114.833	308.982	Gs06410	centrin-2
790	stig_3	113485	113536	intron_retention	268.201	158.48	Gs05990	5-formyltetrahydrofolate cyclo-ligase
791	stig_3	47615	47661	intron_retention	40.1551	37.669	Gs05680	minichromosome maintenance family (MCM)
792	stig_3	203068	203118	intron_retention	342.818	307.872	Gs06590	hypothetical protein Gasu_06590
793	stig_3	77870	77917	intron_retention	39.2562	133.806		
794	stig_3	159647	159752	intron_retention	51.3645	102.438	Gs06330	tetratricopeptide repeat (TPR)-containing protein
795	stig_3	15870	15921	intron_retention	95.7152	118.241		
796	stig_3	21346	21398	intron_retention	302.912	163.191	Gs05520	cleavage and polyadenylation specificity factor subunit-likeprotein
797	stig_3	203266	203315	intron_retention	170.002	163.221	Gs06590	hypothetical protein Gasu_06590
798	stig_3	96372	96420	intron_retention	39.1832	72.1502	Gs05910	TIP41-like family protein
799	stig_3	238904	238950	intron_retention	160.181	139.989	Gs06790	immunophilin isoform 1
800	stig_3	16074	16120	intron_retention	132.991	162.178	Gs05490	PAB-dependent poly(A)-specific ribonuclease subunit 3 isoform 1
801	stig_3	128616	128672	intron_retention	181.232	218.449	Gs06090	hypothetical protein Gasu_06090
802	stig_3	46563	46617	intron_retention	59.7052	76.7365	Gs05670	exosome complex component RRP46
803	stig_3	213601	213652	intron_retention	198.847	147.462	Gs06640	glucoamylase

804	stig_3	46028	46075	intron_retention	186.155	177.243		
805	stig_3	269137	269191	intron_retention	23.8929	40.9739		
806	stig_3	244118	244164	intron_retention	124.14	78.3101	Gs06810	small multidrug efflux protein, DMT family
807	stig_3	57114	57158	intron_retention	462.49	314.32	Gs05730	hypothetical protein Gasu_05730
808	stig_3	7804	7858	intron_retention	316.105	167.082	Gs05440	nucleotide binding protein
809	stig_3	21170	21224	intron_retention	297.922	155.231	Gs05510	aspartic-type endopeptidase
810	stig_3	76358	76425	intron_retention	90.1059	206.777	Gs05810	ribosomal RNA small subunit methyltransferase E
811	stig_3	169374	169520	intron_retention	34.9429	189.676	Gs06400	glycogenin glucosyltransferase-like protein
812	stig_3	214453	214497	intron_retention	142.332	182.068	Gs06650	protein phosphatase 2C
813	stig_3	195247	195302	intron_retention	539.084	611.355	Gs06550	bifunctional aspartyl-tRNA(Asn) / glutamyl-tRNA (Gln)amidotransferase subunit A
814	stig_3	7605	7653	intron_retention	69.8983	61.0833	Gs05440	nucleotide binding protein
815	stig_3	213016	213067	intron_retention	89.3737	60.5753	Gs06640	glucoamylase
816	stig_3	21058	21102	intron_retention	231.119	119.5	Gs05510	aspartic-type endopeptidase
817	stig_3	266991	267041	intron_retention	141.685	212.355		
818	stig_3	133616	133664	intron_retention	359.416	311.403	Gs06130	phosphoglycerate mutase
819	stig_3	162262	162312	intron_retention	190.579	269.91	Gs06340	vesicle-associated membrane protein 7
820	stig_3	157073	157121	intron_retention	171.231	201.983	Gs06320	ubiquitin family protein
821	stig_3	245278	245338	intron_retention	317.88	277.711	Gs06820	electron transfer flavoprotein beta subunit
822	stig_3	133523	133572	intron_retention	301.625	268.176	Gs06130	phosphoglycerate mutase
823	stig_3	5248	5303	intron_retention	47.1068	111.384		
824	stig_3	116479	116539	intron_retention	170.914	220.12	Gs06010	ribonuclease
825	stig_3	185797	185851	intron_retention	366.735	123.834	Gs06490	cytochrome P45, family 51 (sterol 14-demethylase)
826	stig_3	259925	259978	intron_retention	144.204	109.11	Gs06910	hypothetical protein Gasu_06910
827	stig_3	139369	139428	intron_retention	181.704	415.646	Gs06170	hypothetical protein Gasu_06170
828	stig_3	9596	9650	intron_retention	189.519	123.609	Gs05460	transducin family protein / WD-40 repeat family protein
829	stig_3	153035	153081	intron_retention	239.626	182.01	Gs06290	hypothetical protein Gasu_06290
830	stig_3	266691	266746	intron_retention	315.108	524.81	Gs06960	mitochondrial fission 1 protein
831	stig_3	181528	181579	intron_retention	256.415	221.554	Gs06480	ABC transporter, ATP-binding & transmembrane domain
832	stig_3	139116	139168	intron_retention	75.2691	201.616	Gs06170	hypothetical protein Gasu_06170
833	stig_3	142912	142971	intron_retention	249.041	233.14		
834	stig_3	274961	275023	intron_retention	837.0	833.2		
835	stig_3	214034	214082	intron_retention	216.286	241.452	Gs06650	protein phosphatase 2C
836	stig_3	213782	213829	intron_retention	283.948	270.938	Gs06640	glucoamylase
837	stig_3	67301	67349	intron_retention	260.57	206.185	Gs05760	molybdopterin biosynthesis protein MoeA
838	stig_3	134168	134230	intron_retention	233.009	141.877	Gs06130	phosphoglycerate mutase
839	stig_3	54738	54791	intron_retention	773.499	805.599	Gs05710	rubredoxin family protein
840	stig_3	275067	275122	intron_retention	871.226	986.041		
841	stig_3	131757	131806	intron_retention	204.026	203.253		
842	stig_30	136900	136948	intron_retention	53.9691	70.8655		
843	stig_30	77308	77360	intron_retention	221.103	145.79	Gs36400	hypothetical protein Gasu_36400
844	stig_30	38074	38121	intron_retention	402.506	556.419		
845	stig_30	101002	101048	intron_retention	60.0073	69.6971		
846	stig_30	100872	100926	intron_retention	144.225	192.305	Gs36570	hypothetical protein isoform 2

847	stig_30	100594	100653	intron_retention	129.795	155.813	Gs36570	hypothetical protein isoform 2
848	stig_30	125256	125310	intron_retention	116.197	136.692	Gs36680	protein disulfide-isomerase
849	stig_30	137298	137344	intron_retention	37.5256	77.2195	Gs36740	hydrolase family protein / HAD-superfamily protein isoform 2
850	stig_30	96837	96882	intron_retention	745.104	1188.73	Gs36520	hypothetical protein Gasu_36520
851	stig_30	136594	136649	intron_retention	33.0427	46.7949		
852	stig_30	15663	15717	intron_retention	56.3219	100.994		
853	stig_30	125435	125485	intron_retention	95.0214	105.112	Gs36680	protein disulfide-isomerase
854	stig_30	92644	92696	intron_retention	341.911	194.773	Gs36490	N-carbamoylputrescine amidase
855	stig_30	137651	137702	intron_retention	273.836	487.371	Gs36740	hydrolase family protein / HAD-superfamily protein isoform 2
856	stig_30	100413	100462	intron_retention	154.902	171.917	Gs36570	hypothetical protein isoform 2
857	stig_30	81201	81261	intron_retention	71.4433	55.4179		
858	stig_30	96489	96540	intron_retention	286.233	322.671	Gs36520	hypothetical protein Gasu_36520
859	stig_30	60255	60318	intron_retention	338.513	288.261	Gs36280	hypothetical protein Gasu_36280
860	stig_30	101075	101127	intron_retention	258.1	191.947		
861	stig_30	54922	54969	intron_retention	114.612	110.218	Gs36240	fused signal recognition particle receptor
862	stig_31	87764	87818	intron_retention	1193.68	837.186		
863	stig_31	37634	37684	intron_retention	239.574	311.326	Gs36890	hypothetical protein isoform 1
864	stig_31	152468	152540	intron_retention	233.839	281.635	Gs37500	hypothetical protein isoform 1
865	stig_31	152633	152682	intron_retention	225.675	273.922	Gs37500	hypothetical protein isoform 1
866	stig_31	21470	21530	intron_retention	132.528	124.485		
867	stig_31	53624	53675	intron_retention	33.0333	100.961		
868	stig_31	21658	21711	intron_retention	114.48	119.634		
869	stig_31	157857	157908	intron_retention	123.877	475.185	Gs37540	hypothetical protein Gasu_37540
870	stig_31	127115	127169	intron_retention	113.784	189.414	Gs37350	endonuclease isoform 2
871	stig_31	52463	52537	intron_retention	117.967	160.08	Gs36940	hypothetical protein Gasu_36940
872	stig_31	57147	57205	intron_retention	202.694	225.036		
873	stig_31	127316	127365	intron_retention	117.411	150.056	Gs37350	endonuclease isoform 2
874	stig_31	158174	158238	intron_retention	213.182	562.212	Gs37540	hypothetical protein Gasu_37540
875	stig_31	54509	54565	intron_retention	114.031	218.847	Gs36950	BRCA1-associated protein / zinc finger family protein isoform 1
876	stig_31	37191	37244	intron_retention	95.7683	122.858	Gs36890	hypothetical protein isoform 1
877	stig_31	65106	65157	intron_retention	54.764	93.6847	Gs37000	helicase
878	stig_31	143649	143700	intron_retention	184.707	270.09	Gs37440	SNAP receptor/ protein transporter
879	stig_31	73320	73375	intron_retention	124.928	99.2508	Gs37040	hypothetical protein Gasu_37040
880	stig_31	65278	65342	intron_retention	95.2004	154.185	Gs37000	helicase
881	stig_31	73566	73621	intron_retention	232.243	173.421	Gs37050	hypothetical protein Gasu_37050
882	stig_31	47903	47957	intron_retention	373.903	1008.99	Gs36920	hypothetical protein Gasu_36920
883	stig_31	37342	37387	intron_retention	190.237	252.371	Gs36890	hypothetical protein isoform 1
884	stig_31	44590	44646	intron_retention	155.144	123.204	Gs36910	peptidylprolyl isomerase
885	stig_31	157947	157996	intron_retention	129.525	485.814	Gs37540	hypothetical protein Gasu_37540
886	stig_31	57809	57864	intron_retention	53.0705	55.3625	Gs36970	hypothetical protein Gasu_36970
887	stig_31	37745	37798	intron_retention	222.618	263.892	Gs36890	hypothetical protein isoform 1

888	stig_31	108057	108104	intron_retention	83.7881	125.754	Gs37230	hypothetical protein isoform 2
889	stig_31	45704	45765	intron_retention	858.86	601.633	Gs36910	peptidylprolyl isomerase
890	stig_31	88609	88662	intron_retention	191.007	200.513	Gs37120	tRNA (guanine-N7-)-methyltransferase
891	stig_31	49364	49433	intron_retention	144.146	354.481	Gs36930	AAA-type ATPase
892	stig_31	45873	45925	intron_retention	1118.64	736.874	Gs36910	peptidylprolyl isomerase
893	stig_31	100139	100200	intron_retention	250.614	251.031	Gs37190	hypothetical protein Gasu_37190
894	stig_31	158508	158552	intron_retention	66.7234	171.396		
895	stig_31	115123	115176	intron_retention	170.458	87.5898	Gs37270	eukaryotic translation initiation factor-related protein
896	stig_31	87881	87937	intron_retention	231.063	198.913		
897	stig_31	132193	132247	intron_retention	242.447	161.188	Gs37370	ABC transporter, sulfate transport, ATP-binding protein
898	stig_319	2129	2199	intron_retention	80.7421	63.4438	Gs63560	hypothetical protein Gasu_63560, partial
899	stig_32	49875	49932	intron_retention	72.3955	78.6515		
900	stig_32	51677	51724	intron_retention	122.547	181.371	Gs37830	ribosomal large subunit pseudouridine synthase D
901	stig_32	31222	31272	intron_retention	219.735	238.643	Gs37670	transcription initiation factor TFIID subunit D9 isoform 1
902	stig_32	48042	48092	intron_retention	163.267	64.2176	Gs37800	peroxisomal membrane MPV17/PMP22-like protein
903	stig_32	52491	52543	intron_retention	141.32	213.75	Gs37830	ribosomal large subunit pseudouridine synthase D
904	stig_32	67009	67070	intron_retention	506.157	606.559	Gs37930	ubiquitin family protein
905	stig_32	52326	52378	intron_retention	90.1148	110.165	Gs37830	ribosomal large subunit pseudouridine synthase D
906	stig_32	52255	52306	intron_retention	134.128	171.423	Gs37830	ribosomal large subunit pseudouridine synthase D
907	stig_32	71204	71262	intron_retention	174.635	196.293	Gs37960	DNA-directed RNA polymerase I subunit A49 isoform 2
908	stig_32	156796	156856	intron_retention	231.343	233.649		
909	stig_32	66922	66968	intron_retention	318.853	398.453	Gs37930	ubiquitin family protein
910	stig_32	100684	100736	intron_retention	76.8987	113.719	Gs38120	hypothetical protein Gasu_38120
911	stig_32	31112	31165	intron_retention	221.375	276.373	Gs37670	transcription initiation factor TFIID subunit D9 isoform 1
912	stig_32	47865	47921	intron_retention	205.303	97.1126	Gs37800	peroxisomal membrane MPV17/PMP22-like protein
913	stig_32	111759	111814	intron_retention	369.047	734.801	Gs38150	transcription factor
914	stig_32	30418	30472	intron_retention	52.3855	100.085	Gs37660	zinc finger-like protein
915	stig_32	98562	98614	intron_retention	667.93	168.309	Gs38110	hypothetical protein Gasu_38110
916	stig_32	139757	139821	intron_retention	493.412	213.512	Gs38250	isoleucyl-tRNA synthetase isoform 1
917	stig_32	59688	59742	intron_retention	494.615	330.839	Gs37870	hypothetical protein Gasu_37870
918	stig_32	140086	140141	intron_retention	522.913	230.833		
919	stig_32	89297	89361	intron_retention	385.224	151.235	Gs38070	beta-1,4-mannosyl-glycoproteinbeta-1,4-N-acetylglucosaminyltransferase
920	stig_320	1560	1614	intron_retention	44.8462	81.0668		
921	stig_33	97469	97626	intron_retention	341.728	458.77		
922	stig_33	132134	132185	intron_retention	86.8413	31.2011	Gs38930	DNA ligase 4
923	stig_33	39185	39234	intron_retention	115.266	196.94	Gs38490	OTU-like cysteine protease family protein
924	stig_33	26608	26656	intron_retention	89.4001	104.774	Gs38410	hypothetical protein isoform 1
925	stig_33	143306	143351	intron_retention	258.091	130.492	Gs39000	methionine gamma-lyase
926	stig_33	25893	25941	intron_retention	45.3206	65.222	Gs38410	hypothetical protein isoform 1
927	stig_33	114481	114533	intron_retention	208.651	804.01	Gs38860	hypothetical protein Gasu_38860
928	stig_33	34401	34456	intron_retention	104.117	180.938	Gs38450	mannose-6-phosphate isomerase
929	stig_33	125352	125405	intron_retention	166.324	283.02	Gs38900	splicing factor 3A subunit 2
930	stig_33	133968	134021	intron_retention	340.647	272.581	Gs38930	DNA ligase 4

931	stig_33	132631	132685	intron_retention	131.236	55.4862	Gs38930	DNA ligase 4
932	stig_33	141330	141380	intron_retention	127.365	59.6263	Gs38990	hypothetical protein Gasu_38990
933	stig_33	26413	26458	intron_retention	108.18	109.807	Gs38410	hypothetical protein isoform 1
934	stig_33	102042	102089	intron_retention	144.77	197.286	Gs38770	GTP-binding protein
935	stig_33	134310	134467	intron_retention	172.247	187.796		
936	stig_33	139815	139874	intron_retention	244.384	225.097	Gs38980	glutamine amidotransferase, class I isoform 1
937	stig_33	80494	80545	intron_retention	283.292	235.407	Gs38610	phosphatidic acid phosphatase type 2B-like protein
938	stig_33	148986	149037	intron_retention	151.69	107.063	Gs39030	G protein beta subunit-like protein
939	stig_33	109707	109759	intron_retention	79.9948	123.17		
940	stig_33	140745	140793	intron_retention	315.922	84.7005	Gs38990	hypothetical protein Gasu_38990
941	stig_33	81879	81932	intron_retention	748.603	587.413	Gs38620	inorganic phosphate transporter (Pho88)
942	stig_33	141059	141115	intron_retention	208.145	63.0658	Gs38990	hypothetical protein Gasu_38990
943	stig_33	37608	37680	intron_retention	151.971	137.407	Gs38480	mitochondrial carrier
944	stig_33	133292	133347	intron_retention	101.031	89.0983	Gs38930	DNA ligase 4
945	stig_33	37012	37065	intron_retention	490.562	643.205	Gs38470	hypothetical protein isoform 2
946	stig_331	2895	2949	intron_retention	97.2209	222.473	Gs65570	hypothetical protein Gasu_65570
947	stig_331	2651	2708	intron_retention	111.748	221.829	Gs65570	hypothetical protein Gasu_65570
948	stig_331	2588	2635	intron_retention	37.9385	85.3511		
949	stig_335	1454	1506	intron_retention	79.341	203.438	Gs65630	urate oxidase
950	stig_34	77521	77583	intron_retention	256.975	343.158	Gs39400	ABC transporter, cobalt/nickel transport, ATP-binding proteinisoform 2
951	stig_34	120640	120691	intron_retention	279.198	309.91		
952	stig_34	77630	77680	intron_retention	234.619	308.833	Gs39400	ABC transporter, cobalt/nickel transport, ATP-binding proteinisoform 2
953	stig_34	83413	83470	intron_retention	242.862	141.537	Gs39450	4-nitrophenyl phosphatase
954	stig_34	114496	114547	intron_retention	147.903	149.301	Gs39620	ABC transporter, subfamily B, ATP-binding & transmembrane domainisoform 2
955	stig_34	120444	120490	intron_retention	325.825	348.224	Gs39640	hypothetical protein Gasu_39640
956	stig_34	63857	63907	intron_retention	393.463	439.178	Gs39310	hypothetical protein Gasu_39310
957	stig_34	88533	89087	intron_retention	513.006	402.328	Gs39470	ATP-dependent RNA helicase
958	stig_34	96788	96961	intron_retention	722.22	621.318		
959	stig_34	120536	120590	intron_retention	420.461	479.468	Gs39640	hypothetical protein Gasu_39640
960	stig_34	62414	62464	intron_retention	110.567	140.133	Gs39300	hypothetical protein Gasu_39300
961	stig_34	59345	59398	intron_retention	124.43	130.214	Gs39290	transcriptional adapter 2-alpha isoform 1
962	stig_34	137935	137991	intron_retention	225.636	203.859	Gs39760	hypothetical protein Gasu_39760
963	stig_344	1849	1909	intron_retention	68.5467	60.4096		
964	stig_36	126673	126727	intron_retention	640.326	999.793	Gs41340	hypothetical protein Gasu_41340
965	stig_36	126331	126392	intron_retention	417.097	696.333	Gs41340	hypothetical protein Gasu_41340
966	stig_36	112212	112292	intron_retention	27.3215	77.4081		
967	stig_36	91413	91458	intron_retention	190.449	187.925	Gs41150	plastid terminal oxidase
968	stig_36	102760	102825	intron_retention	226.3	274.636	Gs41210	hypothetical protein Gasu_41210
969	stig_36	58576	58638	intron_retention	190.338	266.312	Gs40970	hypothetical protein isoform 2
970	stig_36	28897	28946	intron_retention	187.389	147.425		
971	stig_36	64572	64629	intron_retention	214.043	284.22		

972	stig_36	85636	85685	intron_retention	111.745	150.492	Gs41130	MFS transporter, DHA1 family, tetracycline:hydrogen antiporter
973	stig_36	55930	55979	intron_retention	171.951	235.887	Gs40960	hypothetical protein Gasu_40960
974	stig_36	126492	126559	intron_retention	627.579	985.2	Gs41340	hypothetical protein Gasu_41340
975	stig_36	126162	126215	intron_retention	406.286	738.192	Gs41340	hypothetical protein Gasu_41340
976	stig_36	80817	80873	intron_retention	178.865	303.751	Gs41090	hypothetical protein Gasu_41090
977	stig_36	80962	81012	intron_retention	88.29	174.376		
978	stig_36	83020	83076	intron_retention	63.603	99.4433	Gs41110	mitochondrial carrier
979	stig_36	58684	58759	intron_retention	447.112	506.578	Gs40970	hypothetical protein isoform 2
980	stig_36	46906	46963	intron_retention	141.479	123.19	Gs40900	hypothetical protein Gasu_40900
981	stig_36	88873	88935	intron_retention	144.748	137.348	Gs41140	putative glutathione S-transferase
982	stig_36	56509	56557	intron_retention	76.457	137.771		
983	stig_36	87349	87428	intron_retention	36.1849	272.443		
984	stig_37	71911	71965	intron_retention	493.448	433.209	Gs41720	hypothetical protein isoform 2
985	stig_37	68923	68973	intron_retention	218.179	201.398	Gs41700	cytidine deaminase
986	stig_37	63317	63363	intron_retention	143.28	238.323		
987	stig_37	44495	44545	intron_retention	158.096	85.3394	Gs41570	transcription factor E2F isoform 2
988	stig_37	72022	72069	intron_retention	153.32	261.655	Gs41720	hypothetical protein isoform 2
989	stig_37	104404	104460	intron_retention	146.49	169.624	Gs41890	AP-1 complex subunit gamma-1
990	stig_37	68619	68666	intron_retention	362.329	299.053	Gs41700	cytidine deaminase
991	stig_37	44314	44366	intron_retention	119.425	65.9374	Gs41570	transcription factor E2F isoform 2
992	stig_37	144003	144072	intron_retention	194.482	92.8513	Gs42120	aluminum resistance protein
993	stig_37	49323	49370	intron_retention	511.347	791.436	Gs41600	hypothetical protein Gasu_41600
994	stig_37	44704	44758	intron_retention	179.276	90.346	Gs41570	transcription factor E2F isoform 2
995	stig_37	68798	68848	intron_retention	470.232	386.139	Gs41700	cytidine deaminase
996	stig_37	125473	125615	intron_retention	413.05	541.459	Gs42010	hypothetical protein Gasu_42010
997	stig_37	31172	31345	intron_retention	50.6586	101.315		
998	stig_37	88943	88993	intron_retention	94.0337	24.1762	Gs41810	hypothetical protein isoform 2
999	stig_37	73306	73482	intron_retention	190.229	169.44		
1000	stig_37	93094	93149	intron_retention	59.5686	71.21	Gs41830	hypothetical protein isoform 2
1001	stig_37	33552	33606	intron_retention	54.0145	12.8152	Gs41520	hypothetical protein Gasu_41520
1002	stig_37	112748	112800	intron_retention	244.61	428.922	Gs41940	hypothetical protein Gasu_41940
1003	stig_37	124207	124261	intron_retention	54.0973	88.0804	Gs42000	hypothetical protein Gasu_42000
1004	stig_37	93588	93646	intron_retention	62.185	184.577	Gs41840	DNA excision repair protein ERCC-1
1005	stig_37	49844	49894	intron_retention	218.598	258.255		
1006	stig_37	110195	110249	intron_retention	417.58	221.991		
1007	stig_37	51955	52006	intron_retention	1472.95	175.318	Gs41620	hypothetical protein Gasu_41620
1008	stig_37	95436	95492	intron_retention	527.181	638.405	Gs41850	ubiquitin-conjugating enzyme E2
1009	stig_37	67419	67475	intron_retention	105.696	165.184		
1010	stig_37	114676	114730	intron_retention	68.6739	80.9437	Gs41950*	ABC transporter, iron complex transport, substrate-binding protein
1011	stig_378	1823	1883	intron_retention	118.262	123.321		
1012	stig_38	2412	2467	intron_retention	104.836	133.001	Gs42190	hypothetical protein Gasu_42190
1013	stig_38	51805	51868	intron_retention	284.256	301.223	Gs42500	diacylglycerol kinase

1014	stig_38	11322	11373	intron_retention	44.968	54.8176	Gs42240	metal transporter, ACDP family
1015	stig_38	111861	111907	intron_retention	64.8347	203.845	Gs42810	bifunctional polynucleotide phosphatase/kinase
1016	stig_38	112285	112334	intron_retention	100.392	201.317	Gs42820	hypothetical protein Gasu_42820
1017	stig_38	106103	106165	intron_retention	127.095	174.088	Gs42780	ubiquitin carboxyl-terminal hydrolase 16/45
1018	stig_38	37260	37329	intron_retention	269.052	132.159	Gs42400	adenosylmethionine-8-amino-7-oxononanoate aminotransferase
1019	stig_38	19899	19943	intron_retention	178.825	92.316	Gs42280	alpha-1,2-mannosyltransferase
1020	stig_38	48884	48938	intron_retention	298.288	234.442	Gs42470	SEC14 cytosolic factor family protein / phosphoglyceride transferfamily protein isoform 2
1021	stig_38	51339	51545	intron_retention	223.27	261.779		
1022	stig_38	47501	47553	intron_retention	146.894	107.982	Gs42460	hypothetical protein isoform 2
1023	stig_38	47363	47415	intron_retention	162.713	122.41	Gs42460	hypothetical protein isoform 2
1024	stig_38	50694	50767	intron_retention	100.489	110.73	Gs42490	stromal cell derived factor 2
1025	stig_38	20036	20102	intron_retention	154.914	111.255	Gs42280	alpha-1,2-mannosyltransferase
1026	stig_38	21109	21187	intron_retention	97.5298	318.79	Gs42300	methylase protein
1027	stig_38	66209	66259	intron_retention	70.3946	74.9306	Gs42540	trifunctional protein carbamoyl-phosphate synthasedihydroorotase (CAD protein)
1028	stig_38	4035	4084	intron_retention	243.109	367.971	Gs42200	SsrA-binding protein
1029	stig_38	20853	20905	intron_retention	71.3624	158.397	Gs42290	hypothetical protein Gasu_42290
1030	stig_38	87762	87818	intron_retention	337.271	94.0764	Gs42670	mitochondrial carrier
1031	stig_38	21040	21102	intron_retention	109.004	306.46	Gs42300	methylase protein
1032	stig_38	11095	11143	intron_retention	103.322	101.101	Gs42240	metal transporter, ACDP family
1033	stig_39	126534	126582	intron_retention	64.5508	43.6315	Gs43640	transcription initiation factor TFIIH subunit H1 isoform 2
1034	stig_39	110457	110508	intron_retention	108.562	214.98	Gs43540	hypothetical protein Gasu_43540
1035	stig_39	99803	99850	intron_retention	57.9985	68.343	Gs43470	DNA-directed RNA polymerase III subunit C5
1036	stig_39	58169	58220	intron_retention	117.961	83.7609	Gs43260	pyruvate kinase
1037	stig_39	126411	126463	intron_retention	82.0981	48.0654	Gs43640	transcription initiation factor TFIIH subunit H1 isoform 2
1038	stig_39	118990	119040	intron_retention	72.8306	228.012		
1039	stig_39	128087	128141	intron_retention	150.391	199.583		
1040	stig_39	133340	133392	intron_retention	25.4782	66.9409	Gs43680	hypothetical protein Gasu_43680
1041	stig_39	26380	26436	intron_retention	93.769	111.418	Gs43040	DNA repair protein RAD5
1042	stig_39	128409	128462	intron_retention	894.244	523.769		
1043	stig_39	46013	46066	intron_retention	176.138	191.234	Gs43180	hypothetical protein Gasu_43180
1044	stig_39	107207	107270	intron_retention	35.0554	63.0768	Gs43530	acetyltransferase
1045	stig_39	33452	33500	intron_retention	665.17	603.146		
1046	stig_39	33662	33719	intron_retention	299.708	244.477	Gs43080	peptidyl-tRNA hydrolase, PTH1 family
1047	stig_39	53491	53555	intron_retention	139.682	127.255		
1048	stig_39	35354	35400	intron_retention	91.0215	78.1886	Gs43100	tRNA (guanine-N7-)-methyltransferase
1049	stig_39	34924	34973	intron_retention	328.645	514.067	Gs43090	ubiquitin-conjugating enzyme E2 W
1050	stig_39	34782	34827	intron_retention	507.913	806.577	Gs43090	ubiquitin-conjugating enzyme E2 W
1051	stig_39	136847	136892	intron_retention	277.487	194.429	Gs43700	solute carrier, DMT family
1052	stig_39	107080	107130	intron_retention	172.282	393.74	Gs43530	acetyltransferase
1053	stig_39	133478	133536	intron_retention	89.9998	178.26	Gs43680	hypothetical protein Gasu_43680
1054	stig_39	90595	90649	intron_retention	183.01	233.224	Gs43410	ubiquinone biosynthesis COQ4 family protein

1055	stig_39	25984	26041	intron_retention	144.024	160.784	Gs43040	DNA repair protein RAD5
1056	stig_39	62846	62906	intron_retention	149.332	237.207		
1057	stig_39	104368	104416	intron_retention	148.349	147.556	Gs43500	3-isopropylmalate dehydratase small subunit
1058	stig_39	55890	55943	intron_retention	128.552	157.859	Gs43240	hypothetical protein Gasu_43240
1059	stig_390	446	506	intron_retention	97.1184	85.173		
1060	stig_4	29425	29482	intron_retention	167.938	192.829	Gs07250	alpha/beta hydrolase
1061	stig_4	236500	236548	intron_retention	82.5909	112.359	Gs08410	tRNA pseudouridine synthase A
1062	stig_4	233235	233283	intron_retention	275.174	271.811	Gs08380	potassium channel, VIC family
1063	stig_4	12755	12809	intron_retention	202.996	152.43	Gs07120	U6 snRNA-associated Sm-like protein LSm8
1064	stig_4	12078	12134	intron_retention	96.424	184.286	Gs07110	hypothetical protein Gasu_07110
1065	stig_4	262139	262190	intron_retention	288.532	209.012	Gs08540	hypothetical protein Gasu_08540
1066	stig_4	24786	24851	intron_retention	138.841	156.388	Gs07210	RNA-binding protein NOB1 isoform 2
1067	stig_4	81690	81742	intron_retention	47.5566	90.6364	Gs07570	myb proto-oncogene protein, plant
1068	stig_4	236357	236405	intron_retention	105.753	147.962	Gs08410	tRNA pseudouridine synthase A
1069	stig_4	256133	256185	intron_retention	117.333	87.9835	Gs08500	hypothetical protein Gasu_08500
1070	stig_4	12579	12628	intron_retention	130.272	107.72	Gs07120	U6 snRNA-associated Sm-like protein LSm8
1071	stig_4	10995	11046	intron_retention	34.4863	70.6665	Gs07100	chaperonin GroES
1072	stig_4	153866	153916	intron_retention	35.1463	42.8536	Gs07950	nicotinamide n-methyltransferase
1073	stig_4	171428	171488	intron_retention	549.836	688.216	Gs08030	haloacid dehalogenaselike hydrolase
1074	stig_4	234718	234771	intron_retention	151.053	118.285	Gs08390	diacylglycerol O-acyltransferase
1075	stig_4	241043	241101	intron_retention	116.373	141.98	Gs08430	GTP-binding family protein isoform 2
1076	stig_4	226282	226331	intron_retention	182.549	279.74	Gs08370	NAD+ kinase
1077	stig_4	226461	226514	intron_retention	122.799	180.001	Gs08370	NAD+ kinase
1078	stig_4	18635	18690	intron_retention	182.091	203.593		
1079	stig_4	171010	171058	intron_retention	356.567	416.134	Gs08030	haloacid dehalogenaselike hydrolase
1080	stig_4	204305	204354	intron_retention	102.372	87.2232		
1081	stig_4	134760	134811	intron_retention	203.508	254.95	Gs07820	hypothetical protein Gasu_07820
1082	stig_4	257344	257393	intron_retention	431.264	331.333	Gs08520	ethanolaminephosphotransferase
1083	stig_4	206528	206587	intron_retention	146.767	141.065	Gs08250	tRNA-dihydrouridine synthase 1
1084	stig_4	142525	142582	intron_retention	84.8751	44.2865	Gs07890	phosphatidylserine decarboxylase isoform 1
1085	stig_4	73084	73139	intron_retention	59.7857	102.248	Gs07500	hypothetical protein Gasu_07500
1086	stig_4	53847	53895	intron_retention	43.2782	35.041	Gs07360	hypothetical protein Gasu_07360
1087	stig_4	218187	218249	intron_retention	62.6952	47.7274		
1088	stig_4	63489	63541	intron_retention	195.943	119.274	Gs07440	hypothetical protein Gasu_07440
1089	stig_4	11870	11925	intron_retention	748.198	1155.44	Gs07110	hypothetical protein Gasu_07110
1090	stig_4	221007	221055	intron_retention	153.569	29.9368	Gs08330	hypothetical protein Gasu_08330
1091	stig_4	156394	156443	intron_retention	138.3	114.802	Gs07960	myo-inositol 2-dehydrogenase
1092	stig_4	236061	236112	intron_retention	73.4716	86.521		
1093	stig_4	159120	159173	intron_retention	85.0039	157.38	Gs07980	CDP-diacylglycerol--glycerol-3-phosphate 3-phosphatidyltransferase
1094	stig_4	32051	32106	intron_retention	116.615	223.029	Gs07270	hypothetical protein Gasu_07270
1095	stig_4	31352	31405	intron_retention	105.288	288.562	Gs07260	E3 ubiquitin-protein ligase synoviolin isoform 1
1096	stig_4	31897	31948	intron_retention	169.897	372.934		
1097	stig_4	159281	159328	intron_retention	77.2611	141.576		

1098	stig_4	9586	9667	intron_retention	43.5715	87.2049		
1099	stig_4	72582	72635	intron_retention	191.299	309.692	Gs07500	hypothetical protein Gasu_07500
1100	stig_4	87911	87959	intron_retention	214.804	146.546	Gs07630	cystathionine beta-lyase
1101	stig_4	135517	135580	intron_retention	614.681	736.19		
1102	stig_4	226057	226114	intron_retention	128.796	200.662		
1103	stig_4	170809	170859	intron_retention	385.352	369.58	Gs08030	haloacid dehalogenaselike hydrolase
1104	stig_40	124477	124530	intron_retention	479.719	546.597	Gs44370	peroxisomal membrane MPV17/PMP22-like protein isoform 1
1105	stig_40	29694	29748	intron_retention	1426.8	698.094		
1106	stig_40	34799	34849	intron_retention	228.444	240.974	Gs43900	6-phosphogluconolactonase
1107	stig_40	73308	73352	intron_retention	69.3994	62.8556	Gs44130	dolichyldiphosphatase
1108	stig_40	142651	142701	intron_retention	110.875	135.778		
1109	stig_40	118545	118616	intron_retention	77.8375	68.6414	Gs44320	tRNA-splicing endonuclease subunit Sen54 isoform 1
1110	stig_40	132081	132136	intron_retention	81.0724	109.455	Gs44410	serine/threonine protein kinase
1111	stig_40	30732	30789	intron_retention	87.0008	62.9168	Gs43880	leucine carboxyl methyltransferase family protein isoform 2
1112	stig_40	124247	124426	intron_retention	508.932	579.498		
1113	stig_40	30229	30291	intron_retention	272.428	262.426		
1114	stig_40	142096	142142	intron_retention	76.7394	63.9957	Gs44470	hypothetical protein Gasu_44470
1115	stig_40	73524	73579	intron_retention	52.399	50.2348	Gs44130	dolichyldiphosphatase
1116	stig_40	102131	102185	intron_retention	60.4949	62.1627	Gs44230	4Fe-4S ferredoxin, iron-sulfur binding protein
1117	stig_40	55523	55571	intron_retention	500.328	260.454		
1118	stig_40	38591	38649	intron_retention	445.657	218.696	Gs43930	anthranilate phosphoribosyltransferase
1119	stig_40	118681	118739	intron_retention	59.5676	49.2759	Gs44320	tRNA-splicing endonuclease subunit Sen54 isoform 1
1120	stig_40	143932	143977	intron_retention	218.201	228.889	Gs44480	cytochrome-b5 reductase
1121	stig_40	96374	96435	intron_retention	93.9605	86.5145	Gs44210	peptide chain release factor RF-2
1122	stig_40	29909	29992	intron_retention	544.012	499.333	Gs43870	mitochondrial inner membrane protease IMP1
1123	stig_40	59478	59539	intron_retention	64.8393	41.1101		
1124	stig_40	38162	38213	intron_retention	173.937	67.0017	Gs43930	anthranilate phosphoribosyltransferase
1125	stig_40	60465	60513	intron_retention	172.432	150.402	Gs44030	non-homologous end-joining factor 1
1126	stig_40	58442	58504	intron_retention	96.818	56.3855		
1127	stig_40	39034	39090	intron_retention	415.844	299.73	Gs43930	anthranilate phosphoribosyltransferase
1128	stig_40	118044	118096	intron_retention	120.122	103.238	Gs44320	tRNA-splicing endonuclease subunit Sen54 isoform 1
1129	stig_40	36535	36590	intron_retention	272.428	458.532	Gs43920	kinetochore protein Mis18 isoform 1
1130	stig_40	36941	36998	intron_retention	159.402	204.839	Gs43920	kinetochore protein Mis18 isoform 1
1131	stig_40	36692	36752	intron_retention	278.151	282.704	Gs43920	kinetochore protein Mis18 isoform 1
1132	stig_408	1775	1830	intron_retention	117.877	119.746	Gs66100	hypothetical protein Gasu_66100, partial
1133	stig_41	56032	56084	intron_retention	171.481	161.301	Gs44790	hypothetical protein isoform 1
1134	stig_41	89436	89502	intron_retention	159.264	196.53	Gs44860	glycerol-3-phosphate dehydrogenase (NAD+)
1135	stig_41	55852	55895	intron_retention	302.321	238.215	Gs44790	hypothetical protein isoform 1
1136	stig_41	158356	158460	intron_retention	45.2528	134.274		
1137	stig_41	117283	117338	intron_retention	465.53	463.358	Gs45050	lysyl-tRNA synthetase, class II
1138	stig_41	19172	19231	intron_retention	171.943	253.949		
1139	stig_41	149033	149081	intron_retention	259.346	512.267	Gs45240	bifunctional 2-dehydro-3-deoxyphosphogluconate

								aldolase
1140	stig_41	157224	157274	intron_retention	392.76	326.747	Gs45280	Trp repressor binding protein
1141	stig_41	151986	152033	intron_retention	59.2776	56.8456	Gs45260	exoribonuclease II
1142	stig_41	55368	55417	intron_retention	629.976	827.594	Gs44780	hypothetical protein Gasu_44780
1143	stig_41	63555	63605	intron_retention	67.9943	127.744	Gs44820	exoribonuclease R
1144	stig_41	63755	63808	intron_retention	62.2316	137.126	Gs44820	exoribonuclease R
1145	stig_41	149171	149256	intron_retention	178.881	310.042		
1146	stig_41	130612	130665	intron_retention	227.087	209.382		
1147	stig_41	9955	10046	intron_retention	1346.23	1528.96	Gs44570	adrenodoxin-like ferredoxin 1
1148	stig_41	148702	148748	intron_retention	156.91	298.277	Gs45240	bifunctional 2-dehydro-3-deoxyphosphogluconate aldolase
1149	stig_41	33598	33649	intron_retention	1301.93	1327.67	Gs44670	hypothetical protein Gasu_44670
1150	stig_41	144023	144076	intron_retention	118.575	188.246	Gs45200	mitochondrial carrier (BOU / S-adenosylmethionine carrier)
1151	stig_41	48681	48727	intron_retention	132.844	140.797	Gs44750	DNA mismatch repair protein MutL isoform 1
1152	stig_41	132781	132846	intron_retention	266.142	208.085	Gs45120	hypothetical protein Gasu_45120
1153	stig_41	143859	143913	intron_retention	97.155	180.675	Gs45200	mitochondrial carrier (BOU / S-adenosylmethionine carrier)
1154	stig_41	142803	142866	intron_retention	148.278	202.262	Gs45190	carboxyvinyl-carboxyphosphonate phosphorylmutase
1155	stig_41	124604	124658	intron_retention	163.037	307.962	Gs45080	hypothetical protein Gasu_45080
1156	stig_41	45390	45444	intron_retention	269.946	336.467		
1157	stig_41	124318	124371	intron_retention	214.692	503.404	Gs45080	hypothetical protein Gasu_45080
1158	stig_411	995	1049	intron_retention	31.4682	250.869		
1159	stig_411	1234	1291	intron_retention	38.8428	392.604	Gs66120	hypothetical protein Gasu_66120
1160	stig_42	83258	83315	intron_retention	273.608	290.473	Gs45820	mitochondrial carrier (BOU / S-adenosylmethionine carrier)
1161	stig_42	23911	23962	intron_retention	207.087	165.849	Gs45460	tryptophanyl-tRNA synthetase
1162	stig_42	72588	72640	intron_retention	261.572	252.693	Gs45750	peroxisomal membrane protein PEX12
1163	stig_42	71969	72025	intron_retention	59.7938	77.1393	Gs45750	peroxisomal membrane protein PEX12
1164	stig_42	24783	24834	intron_retention	166.898	153.075		
1165	stig_42	5436	5491	intron_retention	109.065	176.141	Gs45360	hypothetical protein Gasu_45360
1166	stig_42	114004	114054	intron_retention	658.948	536.67	Gs45970	large subunit ribosomal protein L13 isoform 2
1167	stig_42	29557	29614	intron_retention	942.435	637.279	Gs45490	hypothetical protein Gasu_45490
1168	stig_42	72172	72225	intron_retention	91.3296	108.152	Gs45750	peroxisomal membrane protein PEX12
1169	stig_42	93081	93131	intron_retention	330.639	444.511	Gs45870	ubiquinone/menaquinone biosynthesis methyltransferase
1170	stig_42	72400	72451	intron_retention	188.357	211.297	Gs45750	peroxisomal membrane protein PEX12
1171	stig_42	54758	54809	intron_retention	128.986	223.104	Gs45630	hypothetical protein Gasu_45630
1172	stig_42	69442	69495	intron_retention	85.2261	281.931	Gs45730	hypothetical protein Gasu_45730
1173	stig_42	24501	24559	intron_retention	350.576	257.039	Gs45460	tryptophanyl-tRNA synthetase
1174	stig_427	1335	1392	intron_retention	12.6739	40.4459		
1175	stig_43	59050	59101	intron_retention	67.9151	37.4819	Gs46400	carotenoid cis-trans isomerase, CrtH-like protein
1176	stig_43	95001	95049	intron_retention	155.445	153.699		
1177	stig_43	5864	5923	intron_retention	100.907	83.8066		
1178	stig_43	58219	58394	intron_retention	39.2532	68.3942		

1179	stig_43	125326	125383	intron_retention	26.1657	51.5499	Gs46780	chloride channel/carrier, ClC family
1180	stig_43	6267	6318	intron_retention	34.7573	66.8636		
1181	stig_43	58925	58973	intron_retention	46.1679	35.0007	Gs46400	carotenoid cis-trans isomerase, CrtH-like protein
1182	stig_43	48089	48152	intron_retention	153.162	199.174	Gs46340	zinc finger (C3HC4-type RING finger) family protein
1183	stig_43	58028	58188	intron_retention	79.1978	123.098		
1184	stig_43	2691	2751	intron_retention	119.492	78.5594		
1185	stig_43	12548	12598	intron_retention	122.854	131.197	Gs46110	mitochondrial carrier
1186	stig_43	58776	58831	intron_retention	32.5366	29.1757	Gs46400	carotenoid cis-trans isomerase, CrtH-like protein
1187	stig_43	34668	34733	intron_retention	290.031	443.331	Gs46240	hypothetical protein Gasu_46240
1188	stig_43	22080	22141	intron_retention	1318.03	916.613	Gs46170	carboxyl-terminal processing protease
1189	stig_43	21989	22042	intron_retention	94.3832	104.16	Gs46170	carboxyl-terminal processing protease
1190	stig_43	59230	59290	intron_retention	62.79	27.149	Gs46400	carotenoid cis-trans isomerase, CrtH-like protein
1191	stig_43	61002	61059	intron_retention	126.392	72.1871	Gs46410	cyclin-dependent serine/threonine protein kinase
1192	stig_43	103768	103823	intron_retention	210.918	192.38	Gs46690	alpha/beta hydrolase domain-containing protein
1193	stig_43	79725	79773	intron_retention	71.114	69.4842	Gs46540	xylulokinase
1194	stig_43	34969	35034	intron_retention	338.624	484.28		
1195	stig_43	34338	34388	intron_retention	129.175	173.469	Gs46240	hypothetical protein Gasu_46240
1196	stig_43	102641	102688	intron_retention	127.36	153.749	Gs46690	alpha/beta hydrolase domain-containing protein
1197	stig_43	79526	79578	intron_retention	107.096	101.547	Gs46540	xylulokinase
1198	stig_43	111477	111533	intron_retention	20.0647	85.6788		
1199	stig_43	62217	62262	intron_retention	163.69	171.376	Gs46420	GRB2-related adaptor protein 2
1200	stig_43	59538	59590	intron_retention	91.512	46.638	Gs46400	carotenoid cis-trans isomerase, CrtH-like protein
1201	stig_43	62847	62912	intron_retention	471.798	511.609		
1202	stig_43	106328	106372	intron_retention	163.412	134.353	Gs46700	amidophosphoribosyltransferase isoform 2
1203	stig_43	88099	88152	intron_retention	170.217	117.841	Gs46610	hypothetical protein isoform 1
1204	stig_43	94756	94815	intron_retention	392.253	321.552	Gs46630	hypothetical protein Gasu_46630
1205	stig_43	51767	51817	intron_retention	389.293	424.523	Gs46360	hypothetical protein Gasu_46360
1206	stig_43	62472	62523	intron_retention	233.583	275.2	Gs46420	GRB2-related adaptor protein 2
1207	stig_43	61200	61265	intron_retention	183.022	119.005	Gs46410	cyclin-dependent serine/threonine protein kinase
1208	stig_44	92725	92774	intron_retention	87.5763	28.4396	Gs47350	acyl-[ACP]-UDP-N-acetylglucosamine O-acyltransferase isoform 1
1209	stig_44	51760	51820	intron_retention	126.678	90.5543	Gs47170	zinc protease
1210	stig_44	51380	51431	intron_retention	111.979	102.37	Gs47160	nucleotidyltransferase
1211	stig_44	51623	51675	intron_retention	139.735	113.832		
1212	stig_44	64213	64264	intron_retention	72.7093	84.3865	Gs47220	hypothetical protein Gasu_47220
1213	stig_44	29888	29948	intron_retention	119.006	123.465	Gs47010	zinc finger (CCCH-type) family protein isoform 2
1214	stig_44	117927	117981	intron_retention	142.217	244.211	Gs47500	hypothetical protein Gasu_47500
1215	stig_44	117819	117869	intron_retention	409.231	519.505	Gs47500	hypothetical protein Gasu_47500
1216	stig_44	68939	68999	intron_retention	96.7136	139.67		
1217	stig_45	21889	21933	intron_retention	768.275	1303.84		
1218	stig_45	66132	66182	intron_retention	47.8917	52.6881	Gs48060	H3/H4 histone acetyltransferase
1219	stig_45	81383	81454	intron_retention	173.617	152.556		
1220	stig_45	127205	127260	intron_retention	98.3224	125.507		
1221	stig_45	81495	81666	intron_retention	514.88	377.356		

1222	stig_45	50950	51004	intron_retention	54.7316	62.7402		
1223	stig_45	27540	27597	intron_retention	161.93	114.291	Gs47830	vesicle transport protein SEC22
1224	stig_45	96090	96138	intron_retention	149.427	262.158	Gs48220	hypothetical protein Gasu_48220
1225	stig_45	45160	45212	intron_retention	353.925	207.609	Gs47930	poly(A) binding / translation initiation factor
1226	stig_45	19513	19566	intron_retention	457.872	237.072	Gs47760	enoyl-[acyl-carrier protein] reductase I isoform 1
1227	stig_45	66658	66713	intron_retention	88.2146	147.957	Gs48060	H3/H4 histone acetyltransferase
1228	stig_45	72390	72449	intron_retention	199.794	230.35	Gs48100	H/ACA ribonucleoprotein complex subunit 1
1229	stig_45	66503	66555	intron_retention	66.6561	85.5159	Gs48060	H3/H4 histone acetyltransferase
1230	stig_45	94949	95004	intron_retention	256.936	184.203	Gs48210	oxidoreductase NAD-binding domain-containing protein isoform 2
1231	stig_45	52277	52330	intron_retention	108.954	57.6467	Gs47980	hypothetical protein Gasu_47980
1232	stig_45	66338	66388	intron_retention	58.0008	67.8093	Gs48060	H3/H4 histone acetyltransferase
1233	stig_45	31712	31760	intron_retention	313.72	261.213	Gs47860	phosphomannomutase
1234	stig_45	20506	20559	intron_retention	213.51	121.64	Gs47770	hypothetical protein Gasu_47770
1235	stig_45	31908	31960	intron_retention	669.061	488.488	Gs47860	phosphomannomutase
1236	stig_45	128120	128188	intron_retention	61.9442	42.4147		
1237	stig_46	126318	126374	intron_retention	89.0646	237.634		
1238	stig_46	67579	67629	intron_retention	133.324	158.657	Gs48760	DNA helicase II / ATP-dependent DNA helicase PcrA
1239	stig_46	88279	88331	intron_retention	244.453	168.71	Gs48870	glycosyl transferase family protein
1240	stig_46	110418	110466	intron_retention	135.622	267.659	Gs49000	Myb-like DNA-binding protein BAS1 isoform 2
1241	stig_46	87473	87542	intron_retention	118.478	169.328	Gs48860	methyltransferase type 11
1242	stig_46	88112	88159	intron_retention	235.618	175.032	Gs48870	glycosyl transferase family protein
1243	stig_46	61973	62028	intron_retention	174.333	310.308	Gs48700	hypothetical protein Gasu_48700
1244	stig_46	65169	65216	intron_retention	182.697	110.21	Gs48740	serine O-acetyltransferase
1245	stig_46	74779	74829	intron_retention	433.609	306.725	Gs48790	charged multivesicular body protein 1
1246	stig_46	16642	16690	intron_retention	521.417	632.349	Gs48460	methylated-DNA-[protein]-cysteine S-methyltransferase
1247	stig_46	16766	16814	intron_retention	421.614	580.429	Gs48460	methylated-DNA-[protein]-cysteine S-methyltransferase
1248	stig_46	28034	28085	intron_retention	83.659	67.1762		
1249	stig_46	18506	18554	intron_retention	284.262	78.576	Gs48480	uracil-DNA glycosylase
1250	stig_46	120755	120802	intron_retention	301.805	233.077	Gs49070	guanine deaminase
1251	stig_46	19170	19217	intron_retention	80.5817	35.8056	Gs48480	uracil-DNA glycosylase
1252	stig_46	18677	18727	intron_retention	165.325	54.2367	Gs48480	uracil-DNA glycosylase
1253	stig_46	26225	26276	intron_retention	139.268	146.155	Gs48520	hypothetical protein Gasu_48520
1254	stig_46	18798	18855	intron_retention	108.404	43.6504	Gs48480	uracil-DNA glycosylase
1255	stig_46	18986	19036	intron_retention	110.166	43.2488	Gs48480	uracil-DNA glycosylase
1256	stig_46	62829	62892	intron_retention	56.9888	47.1923	Gs48710	hypothetical protein Gasu_48710
1257	stig_46	120511	120562	intron_retention	141.179	112.428		
1258	stig_46	120936	120993	intron_retention	311.885	315.194	Gs49070	guanine deaminase
1259	stig_46	17202	17256	intron_retention	143.515	213.677	Gs48470	AP-3 complex subunit sigma
1260	stig_46	62594	62641	intron_retention	147.018	185.554	Gs48710	hypothetical protein Gasu_48710
1261	stig_46	30923	30971	intron_retention	325.827	191.696	Gs48550	alpha/beta fold family hydrolase
1262	stig_47	53538	53590	intron_retention	89.954	56.5697		
1263	stig_47	15105	15155	intron_retention	117.423	55.6669	Gs49220	hypothetical protein Gasu_49220
1264	stig_47	14824	14875	intron_retention	131.937	69.8646	Gs49220	hypothetical protein Gasu_49220

1265	stig_47	86176	86236	intron_retention	220.846	318.297	Gs49590	DNA-directed RNA polymerase subunit
1266	stig_47	94400	94461	intron_retention	98.3385	111.494		
1267	stig_47	53411	53467	intron_retention	113.939	67.0812		
1268	stig_47	104568	104628	intron_retention	1518.75	998.552	Gs49700	50S ribosomal protein L2
1269	stig_47	120071	120135	intron_retention	92.3353	161.19	Gs49800	hypothetical protein isoform 1
1270	stig_47	52318	52377	intron_retention	182.249	112.401	Gs49430	nonsense-mediated mRNA decay protein 3
1271	stig_47	36955	37005	intron_retention	50.3373	15.0993	Gs49360	origin recognition complex subunit 4 isoform 2
1272	stig_47	78224	78270	intron_retention	57.3113	143.592	Gs49560	shikimate kinase
1273	stig_47	109151	109202	intron_retention	128.715	373.741	Gs49740	hypothetical protein Gasu_49740
1274	stig_47	21468	21512	intron_retention	64.9661	92.8395	Gs49260	hypothetical protein Gasu_49260
1275	stig_47	53317	53367	intron_retention	172.173	96.3025		
1276	stig_47	21202	21259	intron_retention	71.4655	93.6921		
1277	stig_47	53757	53815	intron_retention	116.835	93.5529		
1278	stig_47	11300	11376	intron_retention	259.42	280.152	Gs49190	hypothetical protein isoform 2
1279	stig_47	4282	4330	intron_retention	188.402	239.493	Gs49140	ankyrin-repeat protein
1280	stig_47	17821	17873	intron_retention	38.8056	121.389	Gs49240	5'-AMP-activated protein kinase-related protein
1281	stig_47	36201	36258	intron_retention	94.406	123.528	Gs49360	origin recognition complex subunit 4 isoform 2
1282	stig_47	97173	97234	intron_retention	101.138	152.044	Gs49650	peroxin 14
1283	stig_47	35921	35974	intron_retention	140.886	180.221		
1284	stig_47	36091	36142	intron_retention	170.56	225.399	Gs49360	origin recognition complex subunit 4 isoform 2
1285	stig_47	120817	120871	intron_retention	150.816	160.447		
1286	stig_48	110991	111044	intron_retention	126.521	198.398	Gs50430	hypothetical protein Gasu_50430
1287	stig_48	117632	117684	intron_retention	138.034	314.61	Gs50470	hypothetical protein Gasu_50470
1288	stig_48	43930	43992	intron_retention	166.847	216.492	Gs49990	molybdenum cofactor biosynthesis protein A
1289	stig_48	25076	25131	intron_retention	52.9233	355.252	Gs49900	primary-amine oxidase
1290	stig_48	9878	9938	intron_retention	165.418	186.808		
1291	stig_48	9711	9775	intron_retention	84.6926	95.5612		
1292	stig_48	33838	33886	intron_retention	171.353	338.094	Gs49930	hypothetical protein Gasu_49930
1293	stig_48	75552	75598	intron_retention	243.254	219.739	Gs50150	UDPglucose 6-dehydrogenase isoform 2
1294	stig_48	89068	89126	intron_retention	152.109	61.5601	Gs50260	hypothetical protein Gasu_50260
1295	stig_48	103889	103936	intron_retention	192.936	261.847	Gs50370	hypothetical protein Gasu_50370
1296	stig_48	33558	33613	intron_retention	134.158	213.826	Gs49930	hypothetical protein Gasu_49930
1297	stig_48	93143	93200	intron_retention	45.404	51.3295	Gs50290	hypothetical protein Gasu_50290
1298	stig_48	30608	30661	intron_retention	20.8935	135.488	Gs49920	amino acid/auxin permease, AAAP family
1299	stig_48	117485	117536	intron_retention	172.455	424.261	Gs50470	hypothetical protein Gasu_50470
1300	stig_48	81320	81373	intron_retention	60.7099	68.358	Gs50190	CDP-diacylglycerol--inositol 3-phosphatidyltransferase isoform 2
1301	stig_48	10083	10153	intron_retention	65.8778	69.736		
1302	stig_48	66950	67001	intron_retention	74.6273	135.887	Gs50120	hypothetical protein Gasu_50120
1303	stig_48	81538	81582	intron_retention	65.819	75.8432	Gs50190	CDP-diacylglycerol--inositol 3-phosphatidyltransferase isoform 2
1304	stig_48	54867	54917	intron_retention	215.367	180.849	Gs50050	hypothetical protein isoform 1
1305	stig_48	78354	78405	intron_retention	650.673	325.636	Gs50170	metal ion (Mn2+/Co2+) transporter, MIT family
1306	stig_48	92090	92147	intron_retention	59.0582	156.918	Gs50280	hypothetical protein Gasu_50280

1307	stig_48	55159	55210	intron_retention	102.95	93.5703	Gs50050	hypothetical protein isoform 1
1308	stig_49	50056	50104	intron_retention	299.111	229.041		
1309	stig_49	92634	92685	intron_retention	133.377	30.9921	Gs50990	hypothetical protein isoform 1
1310	stig_49	31386	31434	intron_retention	103.91	144.81	Gs50670	phosphatase / phosphoprotein phosphatase / protein tyrosinephosphatase
1311	stig_49	57258	57310	intron_retention	96.0267	110.44	Gs50830	hypothetical protein Gasu_50830
1312	stig_49	95288	95346	intron_retention	56.0767	51.0193	Gs51010	hypothetical protein isoform 2
1313	stig_49	92324	92491	intron_retention	386.697	189.808	Gs50990	hypothetical protein isoform 1
1314	stig_49	91118	91167	intron_retention	495.865	200.789	Gs50980	protochlorophyllide reductase
1315	stig_49	99074	99144	intron_retention	68.8061	371.138		
1316	stig_49	117259	117319	intron_retention	82.7287	248.093		
1317	stig_49	90729	90783	intron_retention	338.449	144.578	Gs50980	protochlorophyllide reductase
1318	stig_49	90831	90881	intron_retention	463.41	194.977	Gs50980	protochlorophyllide reductase
1319	stig_49	17881	18139	intron_retention	276.162	382.292		
1320	stig_49	51951	52005	intron_retention	295.448	283.701		
1321	stig_49	98988	99048	intron_retention	68.1327	383.36		
1322	stig_49	110449	110501	intron_retention	74.8731	148.644		
1323	stig_49	46715	46782	intron_retention	103.917	125.264	Gs50780	peptidyl-prolyl cis-trans isomerase B (cyclophilin B)
1324	stig_5	111745	111790	intron_retention	115.984	410.716	Gs09280	short-chain dehydrogenase/reductase SDR
1325	stig_5	25345	25397	intron_retention	39.2912	121.762		
1326	stig_5	5179	5227	intron_retention	74.3188	87.7972	Gs08660	tRNA-dihydrouridine synthase 3
1327	stig_5	41409	41459	intron_retention	115.88	117.196	Gs08870	hypothetical protein Gasu_08870
1328	stig_5	95020	95066	intron_retention	73.564	133.538	Gs09190	chromosome transmission fidelity protein 18
1329	stig_5	268512	268568	intron_retention	135.529	48.0633	Gs10100	ADP-ribosylation factor isoform 2
1330	stig_5	230646	230702	intron_retention	728.402	416.952	Gs09860	hypothetical protein Gasu_09860
1331	stig_5	230795	230858	intron_retention	143.292	248.704	Gs09860	hypothetical protein Gasu_09860
1332	stig_5	96676	96731	intron_retention	490.997	383.366	Gs09200	adenylylsulfate (APS) reductase, thioredoxin-independent
1333	stig_5	83533	83587	intron_retention	137.455	158.104	Gs09120	DNA/RNA-binding protein translin/TB-RBP-like protein
1334	stig_5	108391	108440	intron_retention	64.9942	108.087	Gs09260	phosphoserine phosphatase
1335	stig_5	254065	254125	intron_retention	83.581	142.133	Gs09990	hypothetical protein Gasu_09990
1336	stig_5	254574	254620	intron_retention	158.962	301.963	Gs10000	hypothetical protein Gasu_10000
1337	stig_5	237118	237182	intron_retention	114.166	174.39	Gs09900	C-5 sterol desaturase
1338	stig_5	243813	243869	intron_retention	70.5683	53.3888	Gs09930	hypothetical protein Gasu_09930
1339	stig_5	157861	157917	intron_retention	392.565	162.575	Gs09510	hypothetical protein Gasu_09510
1340	stig_5	43967	44016	intron_retention	175.532	175.162	Gs08890	hypothetical protein Gasu_08890
1341	stig_5	253915	253961	intron_retention	69.2383	128.084	Gs09990	hypothetical protein Gasu_09990
1342	stig_5	25946	26004	intron_retention	45.4391	74.2909	Gs08790	alpha-1,4-N-acetylglucosaminyltransferase EXTL3 isoform 1
1343	stig_5	111611	111676	intron_retention	84.8829	302.888	Gs09280	short-chain dehydrogenase/reductase SDR
1344	stig_5	42380	42429	intron_retention	88.1477	25.8976	Gs08880	glutamate N-acetyltransferase
1345	stig_5	96409	96455	intron_retention	408.528	344.863		
1346	stig_5	16197	16251	intron_retention	35.7619	30.5558	Gs08720	DnaJ homolog subfamily C member 11 isoform 2
1347	stig_5	111896	111942	intron_retention	212.951	568.504	Gs09280	short-chain dehydrogenase/reductase SDR
1348	stig_5	112026	112083	intron_retention	347.928	911.625	Gs09280	short-chain dehydrogenase/reductase SDR

1349	stig_5	42228	42279	intron_retention	146.597	76.2662	Gs08880	glutamate N-acetyltransferase
1350	stig_5	214770	214822	intron_retention	179.908	251.32	Gs09760	nucleobase:cation symporter-1, NCS1 family
1351	stig_5	58894	58941	intron_retention	134.364	220.645	Gs08970	glycosyl transferase, group 2 family protein
1352	stig_5	94835	94889	intron_retention	176.737	286.619	Gs09190	chromosome transmission fidelity protein 18
1353	stig_5	208190	208239	intron_retention	135.508	215.896	Gs09720	hypothetical protein Gasu_09720
1354	stig_5	251778	251968	intron_retention	77.6799	68.989		
1355	stig_5	199949	200007	intron_retention	56.9246	83.4922		
1356	stig_5	31577	31629	intron_retention	89.6245	91.6342	Gs08820	N-acetylglucosaminyltransferase
1357	stig_5	65439	65504	intron_retention	56.1445	47.4039	Gs09020	2Fe-2S iron-sulfur cluster binding domain protein
1358	stig_5	191456	191516	intron_retention	78.2611	107.089	Gs09640	hypothetical protein Gasu_09640
1359	stig_5	202872	202916	intron_retention	73.4608	65.7308	Gs09700	hypothetical protein Gasu_09700
1360	stig_5	228585	228827	intron_retention	756.781	303.422		
1361	stig_5	243094	243150	intron_retention	187.224	138.054	Gs09930	hypothetical protein Gasu_09930
1362	stig_5	112238	112289	intron_retention	270.244	750.322	Gs09280	short-chain dehydrogenase/reductase SDR
1363	stig_5	208353	208406	intron_retention	247.74	377.114	Gs09720	hypothetical protein Gasu_09720
1364	stig_5	52111	52163	intron_retention	247.128	198.677	Gs08930	heterogeneous nuclear ribonucleoprotein A1/A3
1365	stig_5	16394	16446	intron_retention	62.1719	42.0775	Gs08720	DnaJ homolog subfamily C member 11 isoform 2
1366	stig_5	102941	102998	intron_retention	60.451	56.036		
1367	stig_5	64080	64131	intron_retention	154.714	114.855	Gs09000	AP-2 complex subunit alpha isoform 2
1368	stig_5	88112	88165	intron_retention	252.67	167.419	Gs09150	hypothetical protein Gasu_09150
1369	stig_5	267799	267851	intron_retention	157.485	154.916	Gs10080	exosome complex component MTR3, animal type
1370	stig_5	254764	254822	intron_retention	368.937	521.578	Gs10000	hypothetical protein Gasu_10000
1371	stig_50	54458	54511	intron_retention	77.1454	87.3704		
1372	stig_50	24916	24966	intron_retention	106.562	257.435	Gs51210	nicotinamidase isoform 2
1373	stig_50	99712	99777	intron_retention	33.2605	48.18	Gs51570	transducin family protein / WD-40 repeat family protein
1374	stig_50	66211	66260	intron_retention	228.817	197.366	Gs51410	GTP-binding protein
1375	stig_50	61470	61520	intron_retention	248.981	369.53		
1376	stig_50	22873	22921	intron_retention	223.434	144.302	Gs51190	U4/U6 small nuclear ribonucleoprotein PRP31
1377	stig_50	53959	54005	intron_retention	110.459	113.566	Gs51360	hypothetical protein isoform 2
1378	stig_50	98221	98274	intron_retention	217.663	177.385	Gs51560	GMP synthase (glutamine-hydrolysing)
1379	stig_50	55370	55426	intron_retention	112.448	164.684	Gs51370	hypothetical protein Gasu_51370
1380	stig_50	25049	25096	intron_retention	63.2173	138.795	Gs51210	nicotinamidase isoform 2
1381	stig_50	109656	109700	intron_retention	102.975	163.222	Gs51620	uracil phosphoribosyltransferase
1382	stig_50	104332	104379	intron_retention	136.438	155.955	Gs51600	protein phosphatase 2 (formerly 2A), regulatory subunit B''
1383	stig_50	54934	54992	intron_retention	90.6107	157.525	Gs51370	hypothetical protein Gasu_51370
1384	stig_50	113784	113827	intron_retention	210.835	151.894	Gs51660	hypothetical protein Gasu_51660
1385	stig_50	22677	22727	intron_retention	110.9	109.097	Gs51190	U4/U6 small nuclear ribonucleoprotein PRP31
1386	stig_50	24154	24200	intron_retention	246.755	219.032		
1387	stig_50	58584	58641	intron_retention	133.452	154.977	Gs51380	hypothetical protein Gasu_51380
1388	stig_50	24652	24702	intron_retention	73.7698	264.953	Gs51210	nicotinamidase isoform 2
1389	stig_50	66645	66698	intron_retention	264.556	234.306	Gs51420*	FMN-binding pyridoxamine 5'-phosphate oxidase-related protein
1390	stig_50	24793	24845	intron_retention	123.707	317.37	Gs51210	nicotinamidase isoform 2
1391	stig_51	99203	99262	intron_retention	121.049	128.055	Gs52250	dTMP kinase

1392	stig_51	13091	13151	intron_retention	80.7999	149.991	Gs51760	two-component sensor histidine kinase
1393	stig_51	37639	37695	intron_retention	180.301	196.183	Gs51920	sedoheptulose-1,7-bisphosphatase, chloroplast
1394	stig_51	59402	59453	intron_retention	123.719	117.413	Gs52080	MFS transporter
1395	stig_51	15676	15741	intron_retention	416.874	552.485	Gs51770	cysteine desulfurase
1396	stig_51	82068	82121	intron_retention	105.315	107.352	Gs52190	AAA-type ATPase
1397	stig_51	81486	81541	intron_retention	31.1603	41.2807	Gs52190	AAA-type ATPase
1398	stig_51	37503	37571	intron_retention	350.935	349.63	Gs51920	sedoheptulose-1,7-bisphosphatase, chloroplast
1399	stig_51	59629	59680	intron_retention	58.6049	70.3014	Gs52080	MFS transporter
1400	stig_51	12575	12625	intron_retention	89.5055	154.338	Gs51750	hypothetical protein isoform 2
1401	stig_51	82350	82401	intron_retention	115.828	103.159	Gs52190	AAA-type ATPase
1402	stig_51	81572	81618	intron_retention	28.8578	29.8844	Gs52190	AAA-type ATPase
1403	stig_51	81931	81983	intron_retention	93.1872	101.415	Gs52190	AAA-type ATPase
1404	stig_51	81318	81369	intron_retention	33.0704	61.2081	Gs52190	AAA-type ATPase
1405	stig_51	31787	31839	intron_retention	56.5394	91.983	Gs51890	exonuclease family protein
1406	stig_51	82211	82262	intron_retention	117.26	107.764	Gs52190	AAA-type ATPase
1407	stig_51	98887	98941	intron_retention	163.24	182.06	Gs52250	dTMP kinase
1408	stig_51	46926	46974	intron_retention	4737.76	593.356	Gs51990	hypothetical protein Gasu_51990
1409	stig_51	44846	44902	intron_retention	173.531	138.855		
1410	stig_51	93825	93946	intron_retention	187.339	142.849	Gs52230	splice factor, putative isoform 1
1411	stig_51	81697	81748	intron_retention	52.2157	51.7658	Gs52190	AAA-type ATPase
1412	stig_52	98028	98080	intron_retention	233.026	391.505	Gs52820	hypothetical protein Gasu_52820
1413	stig_52	13431	13479	intron_retention	85.9355	89.7643	Gs52390	hypothetical protein Gasu_52390
1414	stig_52	75494	75554	intron_retention	115.459	139.375	Gs52670	hypothetical protein Gasu_52670
1415	stig_52	70386	70448	intron_retention	328.534	263.684	Gs52650	U6 snRNA-associated Sm-like protein LSm1
1416	stig_52	95176	95224	intron_retention	145.725	346.39	Gs52790	dihydropteroate synthase
1417	stig_52	24952	25005	intron_retention	249.111	139.828	Gs52440	condensin complex subunit 3
1418	stig_52	50070	50120	intron_retention	42.4802	40.4039	Gs52580	HhH-GPD base excision DNA repair protein-related protein
1419	stig_52	75327	75376	intron_retention	97.3623	130.787	Gs52670	hypothetical protein Gasu_52670
1420	stig_52	96941	96990	intron_retention	138.632	163.746	Gs52810	transcription factor isoform 2
1421	stig_52	45029	45084	intron_retention	206.513	104.682	Gs52540	AP-3 complex subunit beta
1422	stig_52	1626	1686	intron_retention	273.854	135.426	Gs52310	hypothetical protein Gasu_52310
1423	stig_52	25285	25334	intron_retention	247.412	149.926	Gs52440	condensin complex subunit 3
1424	stig_52	100637	100689	intron_retention	109.687	126.662	Gs52840	galactose-1-phosphate uridylyltransferase
1425	stig_53	74289	74359	intron_retention	265.113	238.683	Gs53300	hypothetical protein Gasu_53300
1426	stig_53	62895	62948	intron_retention	192.456	247.521	Gs53240	translation initiation factor IF-3
1427	stig_53	101880	101929	intron_retention	121.354	113.611	Gs53430	WD and tetratricopeptide repeats protein 1 isoform 2
1428	stig_53	7887	7937	intron_retention	175.314	220.843		
1429	stig_53	41475	41524	intron_retention	155.583	137.153	Gs53110	bifunctional methylenetetrahydrofolatedehydrogenase/methenyltetrahydrofolate cyclohydrolase
1430	stig_53	67221	67270	intron_retention	168.074	238.758	Gs53260	DnaJ homolog subfamily C member 6
1431	stig_53	48861	48906	intron_retention	142.614	112.949	Gs53150	hypothetical protein Gasu_53150
1432	stig_53	54754	54821	intron_retention	117.886	135.045	Gs53200	hypothetical protein isoform 1

1433	stig_53	87235	87297	intron_retention	266.101	518.022		
1434	stig_53	12364	12425	intron_retention	559.22	314.804		
1435	stig_54	96374	96434	intron_retention	39.5637	39.625		
1436	stig_54	78135	78191	intron_retention	166.042	188.023	Gs53930	polycomb protein EED
1437	stig_54	2567	2642	intron_retention	180.171	113.804	Gs53470	propionyl-CoA carboxylase beta chain
1438	stig_54	45965	46030	intron_retention	103.931	183.547	Gs53770	hypothetical protein isoform 2
1439	stig_54	14864	14918	intron_retention	70.4331	65.8563	Gs53560	peptidyl-prolyl cis-trans isomerase B (cyclophilin B)
1440	stig_54	35077	35131	intron_retention	1071.33	1410.13		
1441	stig_54	69307	69375	intron_retention	96.698	116.03	Gs53890	3-methylcrotonyl-CoA carboxylase beta subunit isoform 2
1442	stig_54	21949	22014	intron_retention	139.533	339.126	Gs53610	ubiquitin-conjugating enzyme E2
1443	stig_54	31815	31868	intron_retention	310.402	270.121	Gs53670	putative hydrolase of HD superfamily isoform 1
1444	stig_54	24008	24060	intron_retention	130.038	202.617	Gs53630	peptidyl-prolyl cis-trans isomerase isoform 2
1445	stig_54	24367	24430	intron_retention	173.473	225.58	Gs53630	peptidyl-prolyl cis-trans isomerase isoform 2
1446	stig_54	39756	39806	intron_retention	245.999	212.934		
1447	stig_54	24498	24545	intron_retention	141.644	171.44	Gs53630	peptidyl-prolyl cis-trans isomerase isoform 2
1448	stig_54	96159	96229	intron_retention	41.2636	38.9332		
1449	stig_55	76951	77009	intron_retention	57.0379	66.5886	Gs54450	methyltransferase
1450	stig_55	83393	83442	intron_retention	431.431	422.454	Gs54480	adenylyl cyclase-associated protein
1451	stig_55	47694	47747	intron_retention	434.271	457.105		
1452	stig_55	61473	61537	intron_retention	48.5153	251.392		
1453	stig_55	11009	11062	intron_retention	541.054	398.242	Gs54060	hypothetical protein Gasu_54060
1454	stig_55	52040	52090	intron_retention	194.509	134.136		
1455	stig_55	83258	83311	intron_retention	471.156	469.515	Gs54480	adenylyl cyclase-associated protein
1456	stig_55	47142	47204	intron_retention	104.938	170.811	Gs54270	RNA-binding protein
1457	stig_55	52414	52467	intron_retention	210.274	230.748	Gs54310	transducin family protein / WD-40 repeat family protein
1458	stig_55	30712	30760	intron_retention	107.045	39.6273	Gs54170	exonuclease 1
1459	stig_55	47929	47983	intron_retention	375.432	363.412	Gs54280	COP9 signalosome complex subunit 2 isoform 2
1460	stig_55	29281	29345	intron_retention	462.967	350.59		
1461	stig_55	76544	76595	intron_retention	40.9389	52.1827	Gs54450	methyltransferase
1462	stig_55	29746	29798	intron_retention	347.852	175.446	Gs54170	exonuclease 1
1463	stig_55	79298	79349	intron_retention	44.7368	57.7831	Gs54460	aspartyl protease
1464	stig_56	86226	86285	intron_retention	273.018	564.852	Gs54970	mRNA (2'-O-methyladenosine-N6-)-methyltransferase
1465	stig_56	88156	88203	intron_retention	344.53	303.515	Gs54990	hypothetical protein Gasu_54990
1466	stig_56	84928	84980	intron_retention	147.358	482.482	Gs54970	mRNA (2'-O-methyladenosine-N6-)-methyltransferase
1467	stig_56	85786	85832	intron_retention	101.459	257.525	Gs54970	mRNA (2'-O-methyladenosine-N6-)-methyltransferase
1468	stig_56	52277	52336	intron_retention	154.603	124.481	Gs54740	mediator of RNA polymerase II transcription subunit 14 isoform 1
1469	stig_56	34257	34320	intron_retention	23.9105	42.8425		
1470	stig_56	65100	65155	intron_retention	232.519	308.521	Gs54840	hypothetical protein Gasu_54840
1471	stig_56	43756	43807	intron_retention	71.7897	79.8636	Gs54700	mitotic-specific cyclin
1472	stig_56	71280	71332	intron_retention	135.834	165.135	Gs54880	hypothetical protein Gasu_54880
1473	stig_56	85150	85216	intron_retention	80.5187	269.145	Gs54970	mRNA (2'-O-methyladenosine-N6-)-methyltransferase
1474	stig_56	70829	70882	intron_retention	251.983	278.972	Gs54870	elongation factor EF-2
1475	stig_56	88321	88395	intron_retention	271.903	248.088	Gs54990	hypothetical protein Gasu_54990

1476	stig_56	39218	39269	intron_retention	327.117	220.688	Gs54670	deoxyribodipyrimidine photo-lyase isoform 2
1477	stig_56	45789	45841	intron_retention	39.5318	60.3141		
1478	stig_56	39410	39461	intron_retention	334.697	332.418	Gs54670	deoxyribodipyrimidine photo-lyase isoform 2
1479	stig_57	22854	22906	intron_retention	90.0062	160.983	Gs55310	hypothetical protein Gasu_55310
1480	stig_57	2807	2857	intron_retention	181.156	207.458	Gs55190	hypothetical protein Gasu_55190
1481	stig_57	35847	35896	intron_retention	236.393	255.555	Gs55400	nuclear receptor co-repressor 1
1482	stig_57	22587	22638	intron_retention	59.0847	103.942	Gs55310	hypothetical protein Gasu_55310
1483	stig_57	3135	3191	intron_retention	367.775	462.4	Gs55190	hypothetical protein Gasu_55190
1484	stig_57	20969	21020	intron_retention	276.439	282.875	Gs55290	hypothetical protein Gasu_55290
1485	stig_57	57070	57126	intron_retention	121.32	298.311	Gs55480	DNA-directed RNA polymerase III subunit C25 isoform 1
1486	stig_57	39360	39412	intron_retention	57.7256	130.471		
1487	stig_57	2993	3043	intron_retention	353.987	408.037	Gs55190	hypothetical protein Gasu_55190
1488	stig_57	53664	53714	intron_retention	355.539	272.883		
1489	stig_57	2694	2741	intron_retention	136.781	197.752	Gs55190	hypothetical protein Gasu_55190
1490	stig_57	12553	12602	intron_retention	66.9091	74.416		
1491	stig_57	91062	91112	intron_retention	351.153	491.072		
1492	stig_57	91564	91615	intron_retention	191.082	326.564	Gs55660	maltose alpha-D-glucosyltransferase
1493	stig_57	57909	57968	intron_retention	99.3914	59.7297	Gs55490	adenylosuccinate synthase
1494	stig_57	53298	53356	intron_retention	243.432	212.875	Gs55450	hypothetical protein Gasu_55450
1495	stig_57	58095	58148	intron_retention	111.594	83.4908	Gs55490	adenylosuccinate synthase
1496	stig_57	30291	30344	intron_retention	94.1729	76.2149	Gs55370	hypothetical protein Gasu_55370
1497	stig_58	43787	43837	intron_retention	736.092	576.058	Gs55930	U6 snRNA-associated Sm-like protein LSm4 isoform 1
1498	stig_58	31422	31474	intron_retention	174.722	278.489	Gs55850	hypothetical protein Gasu_55850
1499	stig_58	47086	47134	intron_retention	178.492	343.973	Gs55960	hypothetical protein Gasu_55960
1500	stig_58	58746	58796	intron_retention	129.477	100.264	Gs56030	hypothetical protein isoform 1
1501	stig_58	81937	81989	intron_retention	88.7966	93.8557	Gs56150	nucleotide binding protein isoform 1
1502	stig_58	7739	7799	intron_retention	177.052	319.453	Gs55720	GINS complex subunit 4
1503	stig_58	49927	49991	intron_retention	110.875	110.398	Gs55970	tRNA (5-methylaminomethyl-2-thiouridylate)-methyltransferase
1504	stig_58	55190	55237	intron_retention	98.9447	87.9789	Gs56010	hypothetical protein Gasu_56010
1505	stig_58	43943	43990	intron_retention	551.49	461.993	Gs55930	U6 snRNA-associated Sm-like protein LSm4 isoform 1
1506	stig_58	70972	71027	intron_retention	204.296	100.229	Gs56090	hypothetical protein Gasu_56090
1507	stig_58	38155	38212	intron_retention	91.3407	134.027	Gs55890	15,16-dihydrobiliverdin:ferredoxin oxidoreductase
1508	stig_58	55507	55556	intron_retention	215.077	109.223	Gs56010	hypothetical protein Gasu_56010
1509	stig_58	45023	45071	intron_retention	462.976	129.477		
1510	stig_58	69163	69211	intron_retention	281.525	385.07	Gs56070	alpha/beta fold family hydrolase
1511	stig_58	50224	50272	intron_retention	136.432	129.179	Gs55970	tRNA (5-methylaminomethyl-2-thiouridylate)-methyltransferase
1512	stig_58	81551	81601	intron_retention	202.976	191.863	Gs56140	hypothetical protein Gasu_56140
1513	stig_58	55352	55400	intron_retention	164.321	119.118	Gs56010	hypothetical protein Gasu_56010
1514	stig_58	21087	21215	intron_retention	148.25	99.8708	Gs55800	hypothetical protein Gasu_55800
1515	stig_58	55087	55133	intron_retention	67.8621	62.6167	Gs56010	hypothetical protein Gasu_56010
1516	stig_58	14083	14133	intron_retention	266.707	176.535	Gs55760	hypothetical protein Gasu_55760
1517	stig_58	81240	81287	intron_retention	120.339	123.878	Gs56140	hypothetical protein Gasu_56140

1518	stig_58	69986	70037	intron_retention	84.4934	94.9354	Gs56070	alpha/beta fold family hydrolase
1519	stig_59	31215	31273	intron_retention	365.637	423.927	Gs56390	hypothetical protein isoform 1
1520	stig_59	32157	32204	intron_retention	178.1	161.009		
1521	stig_59	73823	73873	intron_retention	312.747	236.237	Gs56600	hypothetical protein Gasu_56600
1522	stig_59	82722	82783	intron_retention	77.9122	86.5834		
1523	stig_59	63888	63939	intron_retention	454.211	327.237	Gs56550	leucine-rich repeat family protein
1524	stig_59	32403	32453	intron_retention	56.8723	56.5029	Gs56400	outer membrane protein
1525	stig_59	88430	88483	intron_retention	334.416	443.905	Gs56680	carbohydrate kinase family isoform 1
1526	stig_59	66117	66160	intron_retention	81.1894	68.9708	Gs56570	sucrose transporter, GPH family
1527	stig_59	31392	31454	intron_retention	244.719	299.028	Gs56390	hypothetical protein isoform 1
1528	stig_59	88870	88923	intron_retention	779.967	556.829	Gs56680	carbohydrate kinase family isoform 1
1529	stig_59	50475	50535	intron_retention	16.6838	91.7013	Gs56500	30S ribosomal protein S1
1530	stig_6	232833	232884	intron_retention	107.272	84.5607	Gs11330	hypothetical protein Gasu_11330
1531	stig_6	194774	194822	intron_retention	501.836	608.597		
1532	stig_6	27457	27511	intron_retention	77.0069	142.854	Gs10340	casein kinase 1 isoform 1
1533	stig_6	97717	97777	intron_retention	186.8	180.372	Gs10650	histone deacetylase 1/2
1534	stig_6	110194	110246	intron_retention	76.4785	120.175	Gs10690	2-oxoglutarate dehydrogenase E1 component isoform 2
1535	stig_6	224423	224474	intron_retention	67.6704	62.582	Gs11280	hypothetical protein Gasu_11280
1536	stig_6	110079	110126	intron_retention	124.097	165.398	Gs10690	2-oxoglutarate dehydrogenase E1 component isoform 2
1537	stig_6	90798	90864	intron_retention	185.326	238.518		
1538	stig_6	169727	169779	intron_retention	49.1487	96.5034		
1539	stig_6	227675	227722	intron_retention	88.438	57.5695	Gs11300	phenazine biosynthesis PhzC/PhzF family protein
1540	stig_6	221932	221983	intron_retention	202.213	141.502	Gs11270	hypothetical protein Gasu_11270
1541	stig_6	209324	209375	intron_retention	141.384	239.381	Gs11210	(S)-2-hydroxy-acid oxidase
1542	stig_6	199881	199928	intron_retention	76.3849	77.2746	Gs11160	ribosomal RNA small subunit methyltransferase B isoform 1
1543	stig_6	171392	171445	intron_retention	205.596	208.897	Gs10980	hypothetical protein Gasu_10980
1544	stig_6	269122	269169	intron_retention	357.918	928.284	Gs11520	ubiquitin-conjugating enzyme E2
1545	stig_6	171730	171788	intron_retention	96.0927	160.224		
1546	stig_6	181254	181309	intron_retention	135.622	152.477		
1547	stig_6	33246	33296	intron_retention	223.194	339.807		
1548	stig_6	265575	265621	intron_retention	89.241	104.948	Gs11500	E3 ubiquitin-protein ligase RAD18
1549	stig_6	239028	239078	intron_retention	155.798	137.974	Gs11350	ubiquitin carboxyl-terminal hydrolase 4/11/15
1550	stig_6	199374	199422	intron_retention	300.847	295.689	Gs11160	ribosomal RNA small subunit methyltransferase B isoform 1
1551	stig_6	244082	244141	intron_retention	536.968	176.704	Gs11390	D-3-phosphoglycerate dehydrogenase
1552	stig_6	214226	214273	intron_retention	48.5966	57.0518	Gs11250	hypothetical protein Gasu_11250
1553	stig_6	228567	228614	intron_retention	66.799	52.6382	Gs11310	pfkB-type carbohydrate kinase family protein
1554	stig_6	205935	205983	intron_retention	257.026	125.306	Gs11190	hypothetical protein Gasu_11190
1555	stig_60	49104	49151	intron_retention	75.18	145.307	Gs56980	pre-mRNA cleavage complex II protein Clp1-like protein
1556	stig_60	14090	14166	intron_retention	154.732	241.885	Gs56790	transcription initiation factor TFIID subunit D8
1557	stig_60	64912	64963	intron_retention	57.9167	45.9518	Gs57090*	isochorismatase hydrolase
1558	stig_60	60992	61050	intron_retention	185.3	354.23	Gs57070	zinc finger (C3HC4-type RING finger) family protein
1559	stig_60	76203	76254	intron_retention	1024.87	812.723	Gs57150	NADH dehydrogenase

1560	stig_60	8499	8550	intron_retention	1697.81	781.435	Gs56750	hypothetical protein Gasu_56750
1561	stig_60	64799	64854	intron_retention	86.3056	85.4628	Gs57090	isochorismatase hydrolase
1562	stig_60	31232	31285	intron_retention	170.446	256.05	Gs56890	phospholipid/glycerol acyltransferase family protein
1563	stig_60	55916	55964	intron_retention	48.1251	45.1724	Gs57020	rhomboid family protein
1564	stig_60	41563	41642	intron_retention	230.69	210.579	Gs56930	3-oxoacyl-(acyl-carrier-protein) reductase
1565	stig_60	58910	58959	intron_retention	297.661	308.111		
1566	stig_60	74684	74742	intron_retention	350.04	400.728	Gs57130	mutS family DNA mismatch repair protein MSH4 isoform 1
1567	stig_60	19446	19497	intron_retention	63.2942	52.3291	Gs56810	glutathione peroxidase
1568	stig_61	20168	20251	intron_retention	155.222	129.998	Gs57340	cytochrome P450 isoform 1
1569	stig_61	69444	69497	intron_retention	293.464	462.467	Gs57690	hypothetical protein Gasu_57690
1570	stig_61	42964	43014	intron_retention	979.187	884.665	Gs57490	prenylated Rab receptor 2
1571	stig_61	42806	42858	intron_retention	851.77	817.633	Gs57490	prenylated Rab receptor 2
1572	stig_61	54719	54776	intron_retention	118.599	118.823	Gs57590	hypothetical protein Gasu_57590
1573	stig_61	56712	56760	intron_retention	155.732	257.942		
1574	stig_61	26836	26879	intron_retention	299.498	383.307		
1575	stig_61	25126	25174	intron_retention	159.871	162.397	Gs57370	hypothetical protein Gasu_57370
1576	stig_61	20363	20416	intron_retention	120.661	133.402	Gs57340	cytochrome P450 isoform 1
1577	stig_61	70147	70200	intron_retention	390.869	747.488	Gs57690	hypothetical protein Gasu_57690
1578	stig_61	69599	69655	intron_retention	39.4893	73.2094	Gs57690	hypothetical protein Gasu_57690
1579	stig_61	58383	58432	intron_retention	373.317	298.84	Gs57610	aminomethyltransferase
1580	stig_61	24937	24988	intron_retention	174.875	146.569	Gs57370	hypothetical protein Gasu_57370
1581	stig_61	44925	44971	intron_retention	398.069	629.933	Gs57510	hypothetical protein Gasu_57510
1582	stig_61	6470	6520	intron_retention	790.438	334.633		
1583	stig_61	70041	70092	intron_retention	295.821	478.892	Gs57690	hypothetical protein Gasu_57690
1584	stig_61	24461	24513	intron_retention	313.696	160.951	Gs57360	Golgi transport protein GOT1-like protein
1585	stig_61	17560	17608	intron_retention	40.2463	41.9087	Gs57320	hypothetical protein isoform 2
1586	stig_61	82841	82896	intron_retention	66.6254	183.776	Gs57770	structural maintenance of chromosome (SMC ATPase family)
1587	stig_61	87365	87422	intron_retention	89.0726	117.352		
1588	stig_61	81844	81903	intron_retention	61.6376	100.268	Gs57760	S-formylglutathione hydrolase
1589	stig_61	49483	49537	intron_retention	38.117	65.5026		
1590	stig_61	81750	81800	intron_retention	84.605	120.622	Gs57760	S-formylglutathione hydrolase
1591	stig_62	2713	2793	intron_retention	200.778	338.525		
1592	stig_62	52985	53029	intron_retention	162.646	176.899	Gs58000	hypothetical protein isoform 1
1593	stig_62	74305	74358	intron_retention	143.256	113.111	Gs58130	hypothetical protein Gasu_58130
1594	stig_62	64910	64960	intron_retention	453.316	161.498		
1595	stig_62	94139	94191	intron_retention	66.5208	112.176	Gs58260	ubiquitin carboxyl-terminal hydrolase L3
1596	stig_62	52790	52848	intron_retention	251.991	195.241	Gs58000	hypothetical protein isoform 1
1597	stig_62	3090	3153	intron_retention	43.0509	103.694		
1598	stig_62	89172	89220	intron_retention	316.738	338.868	Gs58220	bifunctional aspartyl-tRNA(Asn) / glutamyl-tRNA (Gln)amidotransferase subunit A
1599	stig_62	3024	3075	intron_retention	140.738	266.388		
1600	stig_62	88975	89041	intron_retention	300.642	281.122	Gs58220	bifunctional aspartyl-tRNA(Asn) / glutamyl-tRNA (Gln)amidotransferase subunit A

1601	stig_62	50232	50285	intron_retention	179.714	968.889	Gs57980*	glycerol dehydrogenase
1602	stig_62	52529	52586	intron_retention	241.827	175.434		
1603	stig_62	36555	36611	intron_retention	1449.97	1714.97	Gs57910	hypothetical protein Gasu_57910
1604	stig_63	55677	55744	intron_retention	144.244	108.882	Gs58540	peroxisomal membrane MPV17/PMP22-like protein
1605	stig_63	32853	33014	intron_retention	77.1819	202.867		
1606	stig_63	55899	55945	intron_retention	71.4434	70.8314	Gs58540	peroxisomal membrane MPV17/PMP22-like protein
1607	stig_63	9588	9643	intron_retention	69.2599	56.4807		
1608	stig_63	75004	75051	intron_retention	185.22	203.748	Gs58650	hypothetical protein Gasu_58650
1609	stig_63	32654	32832	intron_retention	47.0177	247.707		
1610	stig_63	61336	61390	intron_retention	152.173	155.704	Gs58580	diacylglycerol kinase
1611	stig_63	61124	61178	intron_retention	155.95	167.678	Gs58580	diacylglycerol kinase
1612	stig_63	25369	25425	intron_retention	424.057	957.679		
1613	stig_63	46750	46797	intron_retention	203.029	336.795	Gs58480	hypothetical protein Gasu_58480
1614	stig_64	54737	54793	intron_retention	112.511	88.3811		
1615	stig_64	25357	25405	intron_retention	265.093	88.7047	Gs58830	minichromosome maintenance family (MCM)
1616	stig_64	20019	20075	intron_retention	993.991	582.894	Gs58800	putative carotenoid biosynthesis protein, crtK-like protein
1617	stig_65	37870	37930	intron_retention	407.647	374.105	Gs59310	hypothetical protein Gasu_59310
1618	stig_65	26513	26559	intron_retention	310.078	174.05	Gs59220	proteasome family protein isoform 2
1619	stig_65	29268	29325	intron_retention	289.592	228.002	Gs59240	eukaryotic translation initiation factor SUI1 family protein
1620	stig_65	23919	23992	intron_retention	859.068	1053.96	Gs59200	hypothetical protein Gasu_59200
1621	stig_65	22931	22978	intron_retention	204.382	332.109	Gs59200	hypothetical protein Gasu_59200
1622	stig_65	52714	52761	intron_retention	230.495	189.458	Gs59420	putative acetyltransferase
1623	stig_65	7538	7589	intron_retention	56.4448	87.4844	Gs59120	ribonuclease P
1624	stig_65	11096	11147	intron_retention	187.147	142.832		
1625	stig_66	18179	18228	intron_retention	68.8817	92.9091	Gs59590	hypothetical protein Gasu_59590
1626	stig_66	7001	7056	intron_retention	104.104	118.142	Gs59530	GTP-binding protein LepA
1627	stig_67	22678	22730	intron_retention	131.668	180.456	Gs59850	alpha-1,6-mannosyltransferase
1628	stig_67	51754	51805	intron_retention	164.509	123.433	Gs60030	spermidine synthase
1629	stig_67	29436	29481	intron_retention	106.457	88.6061		
1630	stig_68	21939	21998	intron_retention	100.234	104.615	Gs60210	DNA-3-methyladenine glycosylase
1631	stig_68	21715	21772	intron_retention	156.275	172.565	Gs60210	DNA-3-methyladenine glycosylase
1632	stig_68	4027	4070	intron_retention	143.987	133.519	Gs60080	inorganic phosphate transporter (Pho88)
1633	stig_68	9741	9785	intron_retention	517.936	385.073	Gs60120	hypothetical protein isoform 2
1634	stig_68	10449	10506	intron_retention	107.147	83.4823		
1635	stig_69	38798	38855	intron_retention	242.599	195.192	Gs60550	GNS1/SUR4 membrane family protein
1636	stig_69	37783	37831	intron_retention	137.968	137.473	Gs60540	fibrillin-like protein
1637	stig_69	42179	42230	intron_retention	173.902	245.689	Gs60570	hypothetical protein isoform 2
1638	stig_69	18060	18110	intron_retention	77.7093	55.4789	Gs60430	palmitoyltransferase ZDHHC9
1639	stig_69	22875	22929	intron_retention	465.433	332.185	Gs60470*	mercuric reductase
1640	stig_7	22830	22881	intron_retention	164.319	474.751	Gs11640	hypothetical protein Gasu_11640
1641	stig_7	185157	185209	intron_retention	102.534	179.239	Gs12590	hypothetical protein Gasu_12590
1642	stig_7	156832	156880	intron_retention	166.669	298.977	Gs12460	cob(I)alamin adenosyltransferase
1643	stig_7	123811	123871	intron_retention	233.771	232.681	Gs12280	enoyl-CoA hydratase/ protein tyrosine phosphatase
1644	stig_7	66939	66991	intron_retention	101.665	70.9815	Gs11870	diphosphomevalonate decarboxylase isoform 2

1645	stig_7	193908	193954	intron_retention	97.6989	184.118	Gs12650	hypothetical protein Gasu_12650
1646	stig_7	113536	113592	intron_retention	80.9643	88.7641		
1647	stig_7	185438	185490	intron_retention	181.878	296.625		
1648	stig_7	140822	140871	intron_retention	197.418	162.61	Gs12390	methyltransferase
1649	stig_7	203493	203547	intron_retention	88.5281	120.836	Gs12710	cyclin-dependent serine/threonine protein kinase
1650	stig_7	53192	53268	intron_retention	322.551	160.257	Gs11750	hypothetical protein Gasu_11750
1651	stig_7	85132	85188	intron_retention	36.1489	63.4124	Gs11970	adenosine kinase
1652	stig_7	112984	113033	intron_retention	65.4351	90.4262	Gs12180	transducin family protein / WD-40 repeat family protein isoform 2
1653	stig_7	156536	156588	intron_retention	108.879	213.055	Gs12460	cob(I)alamin adenosyltransferase
1654	stig_7	102220	102267	intron_retention	265.73	361.179		
1655	stig_7	84523	84574	intron_retention	105.556	350.876		
1656	stig_7	162009	162062	intron_retention	50.0653	52.0751	Gs12510	hypothetical protein Gasu_12510
1657	stig_7	24504	24554	intron_retention	198.062	775.898	Gs11650	hypothetical protein Gasu_11650
1658	stig_7	195812	195870	intron_retention	463.777	388.462	Gs12660	hypothetical protein Gasu_12660
1659	stig_7	195494	195548	intron_retention	177.11	109.42	Gs12660	hypothetical protein Gasu_12660
1660	stig_7	91758	91811	intron_retention	151.181	247.535	Gs12020	hypothetical protein Gasu_12020
1661	stig_7	98282	98331	intron_retention	293.861	226.804	Gs12070	carbon-sulfur lyase
1662	stig_7	109318	109365	intron_retention	1062.91	413.035	Gs12160	pyridoxine biosynthesis protein
1663	stig_7	98401	98462	intron_retention	233.894	194.617	Gs12070	carbon-sulfur lyase
1664	stig_7	130477	130529	intron_retention	335.143	291.482	Gs12320	peptidylprolyl isomerase
1665	stig_7	131018	131067	intron_retention	323.188	161.929		
1666	stig_7	210693	210749	intron_retention	449.472	254.586	Gs12770	enoyl-CoA hydratase
1667	stig_7	134490	134537	intron_retention	35.1568	34.2602	Gs12350	hypothetical protein Gasu_12350
1668	stig_7	230239	230296	intron_retention	199.781	626.743		
1669	stig_7	219911	219968	intron_retention	76.2982	82.703	Gs12820	F-box and leucine-rich repeat protein GRR1
1670	stig_7	161736	161785	intron_retention	52.4928	35.6631	Gs12510	hypothetical protein Gasu_12510
1671	stig_7	130671	130720	intron_retention	562.934	337.096	Gs12320	peptidylprolyl isomerase
1672	stig_7	204044	204110	intron_retention	119.062	138.913	Gs12720	hypothetical protein Gasu_12720
1673	stig_7	86117	86178	intron_retention	319.996	392.473	Gs11970	adenosine kinase
1674	stig_7	170261	170306	intron_retention	196.71	102.301	Gs12560	phosphatase
1675	stig_7	59108	59188	intron_retention	246.781	577.765	Gs11820	ribose-phosphate pyrophosphokinase
1676	stig_7	185874	185932	intron_retention	129.276	212.09	Gs12600	ATP-dependent DNA helicase 2 subunit 2 isoform 2
1677	stig_7	119442	119490	intron_retention	75.619	103.33	Gs12240	hypothetical protein Gasu_12240
1678	stig_7	7694	7750	intron_retention	131.623	238.1		
1679	stig_7	138220	138279	intron_retention	96.1635	161.8	Gs12360	tubulin binding protein
1680	stig_7	53341	53422	intron_retention	482.474	252.954	Gs11750	hypothetical protein Gasu_11750
1681	stig_70	29930	29982	intron_retention	369.966	527.387	Gs60730	hypothetical protein Gasu_60730
1682	stig_70	33480	33529	intron_retention	360.888	698.581	Gs60760	DNA polymerase epsilon subunit 4
1683	stig_70	23315	23376	intron_retention	461.501	588.191	Gs60690	S-methyl-5'-thioadenosine phosphorylase
1684	stig_70	16182	16235	intron_retention	514.89	802.196	Gs60660	5-oxopent-3-end-1,2,5-tricarboxylate decarboxylase
1685	stig_70	9380	9426	intron_retention	121.936	106.539		
1686	stig_70	23591	23638	intron_retention	197.315	279.234	Gs60700	transcription elongation factor B, polypeptide 1
1687	stig_71	7135	7186	intron_retention	539.946	263.658	Gs60810	hypothetical protein Gasu_60810

1688	stig_73	2149	2226	intron_retention	36.8388	83.7488		
1689	stig_73	5560	5620	intron_retention	38.5213	59.2161		
1690	stig_73	2904	2959	intron_retention	62.5745	134.814		
1691	stig_74	5435	5486	intron_retention	444.539	404.493	Gs60990	hemolysin III-like protein isoform 1
1692	stig_8	139945	140002	intron_retention	167.009	213.079	Gs13770	DnaJ (Hsp40) homolog, subfamily B
1693	stig_8	60900	60952	intron_retention	195.291	148.541	Gs13350	hypothetical protein Gasu_13350
1694	stig_8	154630	154680	intron_retention	148.127	148.673	Gs13840	potassium channel tetramerisation domain-like protein
1695	stig_8	39105	39164	intron_retention	462.893	362.409	Gs13210	endodeoxyribonuclease isoform 1
1696	stig_8	40436	40498	intron_retention	332.339	202.631	Gs13230	hypothetical protein isoform 2
1697	stig_8	60379	60442	intron_retention	140.73	98.4692	Gs13350	hypothetical protein Gasu_13350
1698	stig_8	160735	160792	intron_retention	126.27	200.918	Gs13880	hypothetical protein Gasu_13880
1699	stig_8	215982	216038	intron_retention	112.048	83.7025	Gs14150	farnesyltransferase / geranylgeranyltransferase isoform 1
1700	stig_8	137738	137802	intron_retention	204.15	206.635	Gs13750	ADP-ribosylation factor GTPase-activating protein 1
1701	stig_8	10905	10953	intron_retention	42.7075	78.2281	Gs13050	hypothetical protein Gasu_13050
1702	stig_8	37837	37899	intron_retention	38.1776	27.4884	Gs13210	endodeoxyribonuclease isoform 1
1703	stig_8	4641	4700	intron_retention	105.55	109.725		
1704	stig_8	139188	139239	intron_retention	94.2108	177.773	Gs13760	syntaxin 1B/2/3
1705	stig_8	199397	199443	intron_retention	115.194	245.094	Gs14050	hypothetical protein Gasu_14050
1706	stig_8	39341	39399	intron_retention	303.12	280.493		
1707	stig_8	124109	124162	intron_retention	71.2419	63.028	Gs13690	chloride channel/carrier, CIC family
1708	stig_8	125084	125149	intron_retention	55.0161	38.1212	Gs13690	chloride channel/carrier, CIC family
1709	stig_8	53721	53774	intron_retention	92.9092	96.8413	Gs13300	hypothetical protein Gasu_13300
1710	stig_8	102296	102345	intron_retention	442.28	368.085	Gs13570	serine/threonine protein kinase
1711	stig_8	9736	9784	intron_retention	136.25	128.627	Gs13040	protein tyrosine phosphatase
1712	stig_8	9612	9672	intron_retention	176.324	149.117	Gs13040	protein tyrosine phosphatase
1713	stig_8	178474	178534	intron_retention	289.371	1124.41	Gs13970	zinc finger protein
1714	stig_8	188680	188732	intron_retention	336.535	211.152		
1715	stig_8	247187	247252	intron_retention	34.9866	48.8075	Gs14330	dihydroorotase
1716	stig_8	49367	49426	intron_retention	117.055	122.669		
1717	stig_8	154348	154395	intron_retention	324.419	281.07	Gs13840	potassium channel tetramerisation domain-like protein
1718	stig_8	70983	71040	intron_retention	191.432	172.822		
1719	stig_8	215786	215844	intron_retention	138.471	136.487		
1720	stig_8	65884	65954	intron_retention	440.958	159.877	Gs13370	hypothetical protein Gasu_13370
1721	stig_8	207926	207971	intron_retention	272.269	287.82	Gs14090	maintenance of ploidy protein MOB1 (MPS1 binder 1)
1722	stig_8	70783	70837	intron_retention	288.929	202.408	Gs13390	uroporphyrinogen-III synthase
1723	stig_8	189002	189055	intron_retention	151.055	160.837	Gs14020	sorting nexin 1 / phosphoinositide binding protein isoform 1
1724	stig_8	25120	25174	intron_retention	122.374	74.2597	Gs13140	kinesin-related protein
1725	stig_8	171945	171995	intron_retention	109.329	23.3162	Gs13930	methyltransferase
1726	stig_8	208082	208137	intron_retention	219.911	218.359	Gs14090	maintenance of ploidy protein MOB1 (MPS1 binder 1)
1727	stig_8	49609	49659	intron_retention	186.825	181.735		
1728	stig_8	211194	211248	intron_retention	49.2507	84.9014	Gs14110	hypothetical protein Gasu_14110
1729	stig_81	10994	11044	intron_retention	392.976	258.933	Gs61200	cytochrome-b5 reductase
1730	stig_81	5008	5057	intron_retention	242.754	116.237	Gs61170	NAD-dependent epimerase/dehydratase

1731	stig_81	4506	4560	intron_retention	43.7089	26.652	Gs61170	NAD-dependent epimerase/dehydratase
1732	stig_83	5860	5920	intron_retention	254.661	216.624		
1733	stig_83	5686	5732	intron_retention	316.122	269.51		
1734	stig_86	10984	11040	intron_retention	58.4489	268.583		
1735	stig_87	3921	3981	intron_retention	389.105	242.559		
1736	stig_88	1549	1595	intron_retention	23.3975	51.2487		
1737	stig_89	2806	2852	intron_retention	119.207	62.5527		
1738	stig_9	40607	40661	intron_retention	154.131	133.238	Gs14580	hypothetical protein Gasu_14580
1739	stig_9	42078	42124	intron_retention	226.474	200.24	Gs14600	hypothetical protein isoform 2
1740	stig_9	200898	200949	intron_retention	188.74	82.1338		
1741	stig_9	232255	232303	intron_retention	168.865	172.239		
1742	stig_9	41626	41675	intron_retention	418.993	367.249	Gs14590	peptidylprolyl isomerase
1743	stig_9	41279	41332	intron_retention	375.404	304.176	Gs14590	peptidylprolyl isomerase
1744	stig_9	101461	101527	intron_retention	34.3919	50.4754		
1745	stig_9	8919	9093	intron_retention	193.366	269.966		
1746	stig_9	144605	144651	intron_retention	137.921	56.1906	Gs15090	hypothetical protein isoform 1
1747	stig_9	115288	115341	intron_retention	126.496	99.497	Gs14950	hypothetical protein isoform 2
1748	stig_9	232384	232437	intron_retention	137.338	153.084		
1749	stig_9	230232	230294	intron_retention	145.848	173.876	Gs15490	prephenate dehydratase
1750	stig_9	80609	80659	intron_retention	36.049	56.3861	Gs14720	hypothetical protein Gasu_14720
1751	stig_9	48082	48181	intron_retention	145.564	160.372	Gs14640	alpha-amylase
1752	stig_9	16579	16627	intron_retention	349.339	237.804	Gs14430	GCN5-related N-acetyltransferase (GNAT) family protein isoform 1
1753	stig_9	41443	41489	intron_retention	571.157	451.195	Gs14590	peptidylprolyl isomerase
1754	stig_9	153313	153366	intron_retention	95.7732	31.7934	Gs15110	dihydrolipoamide dehydrogenase
1755	stig_9	43489	43544	intron_retention	62.2234	37.991	Gs14610	coproporphyrinogen III oxidase isoform 2
1756	stig_9	86274	86334	intron_retention	231.764	398.789		
1757	stig_9	104388	104446	intron_retention	292.045	209.671	Gs14870	methylene-fatty-acyl-phospholipid synthase
1758	stig_9	872	918	intron_retention	60.1816	84.6618		
1759	stig_9	201229	201281	intron_retention	66.8173	138.016	Gs15350	hypothetical protein isoform 1
1760	stig_9	43290	43338	intron_retention	68.4616	41.62	Gs14610	coproporphyrinogen III oxidase isoform 2
1761	stig_9	232123	232176	intron_retention	146.468	149.849	Gs15510	hypothetical protein Gasu_15510
1762	stig_9	201442	201487	intron_retention	56.5598	124.194	Gs15350	hypothetical protein isoform 1
1763	stig_9	212463	212518	intron_retention	133.433	127.986	Gs15420	ER lumen protein retaining receptor
1764	stig_9	42895	42948	intron_retention	134.726	63.5541	Gs14610	coproporphyrinogen III oxidase isoform 2
1765	stig_94	8064	8111	intron_retention	96.083	158.347	Gs61510	hydrolase
1766	stig_94	9546	9598	intron_retention	224.267	146.451	Gs61520	hypothetical protein Gasu_61520
