# Supplementary material for: Unexpected conservation of the RNA splicing apparatus in the highly streamlined genome of Galdieria sulphuraria
Source: BMC Evol Biol. 2018 Apr 2;18:41. doi: 10.1186/s12862-018-1161-x (PMC5880011; doi:10.1186/s12862-018-1161-x)
Supplement: Supplementary file 15 — Table S7. Galdieria sulphuraria introns that were differentially spliced under the heat and cold conditions. (PDF 226 kb) [file 12862_2018_1161_MOESM15_ESM.pdf]

Table S7. ***Galdieria sulphuraria*** **introns that were differentially spliced under the heat and cold conditions** (Examples displayed in Fig. 3C are shown in red text)

No.	Chr	Start	End	Category	Stat_diff_trans [1]	Sqrt JSD [2]	Coverage (heat)	Coverage (cold)	Gene	Product
1	stig_3	169374	169520	intron_retention	0.502	0.302	34.9	189.7	Gs06400	glycogenin glucosyltransferase-like protein
2	stig_5	16197	16251	intron_retention	0.523	0.268	35.8	30.6	Gs08720	DnaJ homolog subfamily C member 11 isoform 2
3	stig_28	74962	75041	intron_retention	0.636	0.266	11.1	121.0		
4	stig_25	60898	60953	intron_retention	0.677	0.253	43.3	60.6	Gs32000	peptidylprolyl isomerase
5	stig_23	33285	33339	intron_retention	0.776	0.302	87.0	97.2		
6	stig_27	63628	63678	intron_retention	0.905	0.293	33.2	29.6	Gs33740	hypothetical protein Gasu_33740
7	stig_0	357640	357695	intron_retention	0.974	0.266	136.6	52.6	Gs01940	hypothetical protein isoform 1
8	stig_46	19170	19217	intron_retention	0.975	0.417	80.6	35.8	Gs48480	uracil-DNA glycosylase
9	stig_37	33552	33606	intron_retention	0.984	0.317	54.0	12.8	Gs41520	hypothetical protein Gasu_41520
10	stig_52	45029	45084	intron_retention	0.991	0.318	206.5	104.7	Gs52540	AP-3 complex subunit beta
11	stig_50	54934	54992	intron_retention	1.007	0.392	90.6	157.5	Gs51370	hypothetical protein Gasu_51370
12	stig_45	66658	66713	intron_retention	1.015	0.345	88.2	148.0	Gs48060	H3/H4 histone acetyltransferase
13	stig_61	44925	44971	intron_retention	1.026	0.293	398.1	629.9	Gs57510	hypothetical protein Gasu_57510
14	stig_39	136847	136892	intron_retention	1.041	0.302	277.5	194.4	Gs43700	solute carrier, DMT family
15	stig_40	59478	59539	intron_retention	1.052	0.386	64.8	41.1		
16	stig_33	141330	141380	intron_retention	1.083	0.342	127.4	59.6	Gs38990	hypothetical protein Gasu_38990
17	stig_32	98562	98614	intron_retention	1.085	0.273	667.9	168.3	Gs38110	hypothetical protein Gasu_38110
18	stig_11	214890	214944	intron_retention	1.086	0.330	63.4	104.6		
19	stig_48	30608	30661	intron_retention	1.110	0.427	20.9	135.5	Gs49920	amino acid/auxin permease, AAAP family
20	stig_61	6470	6520	intron_retention	1.138	0.298	790.4	334.6		
21	stig_12	73308	73365	intron_retention	1.166	0.289	202.8	139.9	Gs18490	7,8-dihydro-8-oxoguanine triphosphatase
22	stig_55	29746	29798	intron_retention	1.183	0.274	347.9	175.4	Gs54170	exonuclease 1
23	stig_1	41832	41886	intron_retention	1.198	0.278	79.9	63.8	Gs02600	hypothetical protein Gasu_02600
24	stig_31	49364	49433	intron_retention	1.230	0.334	144.1	354.5	Gs36930	AAA-type ATPase
25	stig_34	62414	62464	intron_retention	1.232	0.366	110.6	140.1	Gs39300	hypothetical protein Gasu_39300
26	stig_46	18798	18855	intron_retention	1.235	0.281	108.4	43.7	Gs48480	uracil-DNA glycosylase
27	stig_6	265575	265621	intron_retention	1.243	0.276	89.2	104.9	Gs11500	E3 ubiquitin-protein ligase RAD18
28	stig_6	239028	239078	intron_retention	1.323	0.265	155.8	138.0	Gs11350	ubiquitin carboxyl-terminal hydrolase
29	stig_24	144015	144070	intron_retention	1.360	0.251	157.0	85.1	Gs31510	band 7 family protein isoform 2
30	stig_5	16394	16446	intron_retention	1.382	0.279	62.2	42.1	Gs08720	DnaJ homolog subfamily C member 11
31	stig_26	91165	91231	intron_retention	1.383	0.291	71.3	91.7	Gs33050	hypothetical protein Gasu_33050
32	stig_21	23549	23598	intron_retention	1.384	0.287	139.3	188.0	Gs27760	sugar-phosphate:phosphate translocator
33	stig_241	1078	1131	intron_retention	1.401	0.279	75.0	96.1		
34	stig_57	52662	52984	alter_trans_start/end	1.402	0.256	78.3	92.5	Gs55450	hypothetical protein Gasu_55450

35	stig_45	66338	66388	intron_retention	1.406	0.254	58.0	67.8	Gs48060	H3/H4 histone acetyltransferase
36	stig_7	219911	219968	intron_retention	1.412	0.254	76.3	82.7	Gs12820	F-box and leucine-rich repeat protein GRR1
37	stig_55	79298	79349	intron_retention	1.422	0.321	44.7	57.8	Gs54460	aspartyl protease
38	stig_26	63565	63621	intron_retention	1.430	0.253	284.0	322.1	Gs32900	hypothetical protein Gasu_32900
39	stig_39	133478	133536	intron_retention	1.432	0.466	90.0	178.3	Gs43680	hypothetical protein Gasu_43680
40	stig_15	35469	35519	intron_retention	1.441	0.263	113.0	190.9	Gs21350	NADH dehydrogenase
41	stig_3	153035	153081	intron_retention	1.446	0.255	239.6	182.0	Gs06290	hypothetical protein Gasu_06290
42	stig_47	17821	17873	intron_retention	1.457	0.441	38.8	121.4	Gs49240	5'-AMP-activated protein kinase-related protein
43	stig_10	73119	73172	intron_retention	1.485	0.344	32.4	36.6	Gs15970	Myb-like DNA-binding protein REB1
44	stig_15	29179	29235	intron_retention	1.491	0.278	65.2	72.1		
45	stig_61	70041	70092	intron_retention	1.492	0.309	295.8	478.9	Gs57690	hypothetical protein Gasu_57690
46	stig_126	5859	5936	intron_retention	1.495	0.385	20.8	70.1		
47	stig_2	115157	115210	intron_retention	1.497	0.271	116.6	174.0	Gs04450	D-lactate dehydrogenase (cytochrome)
48	stig_27	87456	87513	intron_retention	1.508	0.431	11.7	100.9		
49	stig_9	872	918	intron_retention	1.523	0.279	60.2	84.7		
50	stig_47	36201	36258	intron_retention	1.525	0.284	94.4	123.5	Gs49360	origin recognition complex subunit 4 isoform 2
51	stig_46	120511	120562	intron_retention	1.535	0.310	141.2	112.4		
52	stig_5	64080	64131	intron_retention	1.550	0.256	154.7	114.9	Gs09000	AP-2 complex subunit alpha isoform 2
53	stig_34	59345	59398	intron_retention	1.557	0.306	124.4	130.2	Gs39290	transcriptional adapter 2-alpha isoform 1
54	stig_14	31869	31914	intron_retention	1.559	0.362	74.2	88.5	Gs20390	hypothetical protein Gasu_20390
55	stig_4	32051	32106	intron_retention	1.571	0.295	116.6	223.0	Gs07270	hypothetical protein Gasu_07270
56	stig_22	153248	153323	intron_retention	1.576	0.335	485.3	645.9		
57	stig_11	153036	153111	intron_retention	1.576	0.318	43.7	230.6		
58	stig_20	38230	38281	intron_retention	1.605	0.512	39.5	42.1	Gs26690	DNA repair and recombination protein RAD52
59	stig_51	81318	81369	intron_retention	1.606	0.432	33.1	61.2	Gs52190	AAA-type ATPase
60	stig_10	97512	97576	intron_retention	1.628	0.320	207.3	114.8	Gs16100	glutamate decarboxylase isoform 2
61	stig_11	98704	98759	intron_retention	1.661	0.340	48.4	77.0	Gs17360	short-chain dehydrogenase/reductase SDR
62	stig_4	31352	31405	intron_retention	1.685	0.364	105.3	288.6	Gs07260	E3 ubiquitin-protein ligase synoviolin isoform 1
63	stig_47	35921	35974	intron_retention	1.685	0.253	140.9	180.2		
64	stig_10	90952	91006	intron_retention	1.709	0.361	162.5	188.3	Gs16070	isocitrate dehydrogenase, NADP dependent
65	stig_22	124156	124210	intron_retention	1.718	0.264	58.3	207.0	Gs29430	ABC transporter, ATP-binding & transmembrane domain
66	stig_29	40870	40925	intron_retention	1.744	0.278	222.4	117.9	Gs35420	RNA polymerase primary sigma factor
67	stig_40	60465	60513	intron_retention	1.756	0.305	172.4	150.4	Gs44030	non-homologous end-joining factor 1
68	stig_14	92647	92702	intron_retention	1.758	0.362	175.2	135.5		
69	stig_164	906	958	intron_retention	1.758	0.314	357.4	193.2	Gs63270	arsenate reductase

70	stig_10	33767	33823	intron_retention	1.759	0.251	329.4	280.4	Gs15780	peptidase S58 DmpA isoform 1
71	stig_54	24008	24060	intron_retention	1.776	0.335	130.0	202.6	Gs53630	peptidyl-prolyl cis-trans isomerase isoform 2
72	stig_51	31787	31839	intron_retention	1.785	0.258	56.5	92.0	Gs51890	exonuclease family protein
73	stig_31	115123	115176	intron_retention	1.799	0.291	170.5	87.6	Gs37270	eukaryotic translation initiation factor-related protein
74	stig_40	58442	58504	intron_retention	1.799	0.499	96.8	56.4		
75	stig_27	13851	13904	intron_retention	1.816	0.262	106.6	72.1	Gs33490	protein arginine N-methyltransferase 5 isoform 1
76	stig_61	82841	82896	intron_retention	1.818	0.381	66.6	183.8	Gs57770	structural maintenance of chromosome (SMC ATPase family)
77	stig_19	21806	21952	intron_retention	1.830	0.514	188.4	51.5	Gs25760	omega-6 fatty acid desaturase (delta-12 desaturase)
78	stig_47	36091	36142	intron_retention	1.832	0.258	170.6	225.4	Gs49360	origin recognition complex subunit 4 isoform 2
79	stig_10	97101	97153	intron_retention	1.837	0.398	79.3	91.7	Gs16100	glutamate decarboxylase isoform 2
80	stig_49	117259	117319	intron_retention	1.838	0.288	82.7	248.1		
81	stig_4	9586	9667	intron_retention	1.900	0.271	43.6	87.2		
82	stig_18	78218	78282	intron_retention	1.926	0.368	51.4	84.3	Gs25010	aminomethyltransferase
83	stig_9	201229	201281	intron_retention	1.936	0.277	66.8	138.0	Gs15350	hypothetical protein isoform 1
84	stig_6	214226	214273	intron_retention	1.981	0.445	48.6	57.1	Gs11250	hypothetical protein Gasu_11250
85	stig_54	24367	24430	intron_retention	1.995	0.306	173.5	225.6	Gs53630	peptidyl-prolyl cis-trans isomerase isoform 2
86	stig_61	87365	87422	intron_retention	1.997	0.291	89.1	117.4		
87	stig_5	88112	88165	intron_retention	2.015	0.299	252.7	167.4	Gs09150	hypothetical protein Gasu_09150
88	stig_41	144023	144076	intron_retention	2.027	0.502	118.6	188.2	Gs45200	mitochondrial carrier (BOU / S-adenosylmethionine carrier)
89	stig_51	82211	82262	intron_retention	2.053	0.287	117.3	107.8	Gs52190	AAA-type ATPase
90	stig_9	43290	43338	intron_retention	2.100	0.402	68.5	41.6	Gs14610	coproporphyrinogen III oxidase isoform 2
91	stig_56	45789	45841	intron_retention	2.127	0.477	39.5	60.3		
92	stig_17	103420	103472	intron_retention	2.142	0.251	471.7	209.9	Gs24100	peroxisomal membrane protein-related protein
93	stig_8	70783	70837	intron_retention	2.149	0.277	288.9	202.4	Gs13390	uroporphyrinogen-III synthase
94	stig_23	123492	123547	intron_retention	2.168	0.273	247.4	257.6	Gs30470	endonuclease III isoform 2
95	stig_7	59108	59188	intron_retention	2.176	0.301	246.8	577.8	Gs11820	ribose-phosphate pyrophosphokinase
96	stig_26	92387	92538	intron_retention	2.231	0.251	42.1	61.9		
97	stig_55	78858	79819	alter_trans_start/end	2.231	0.256	97.6	88.9	Gs54460	aspartyl protease
98	stig_57	53298	53356	intron_retention	2.240	0.264	243.4	212.9	Gs55450	hypothetical protein Gasu_55450
99	stig_19	89275	89333	intron_retention	2.276	0.260	183.5	85.1		
100	stig_11	20488	20548	intron_retention	2.361	0.270	122.6	109.8	Gs16900	20S proteasome subunit alpha 7
101	stig_25	86289	86356	intron_retention	2.362	0.297	100.4	157.8	Gs32160	V-type H+-transporting ATPase subunit c isoform 2
102	stig_14	111789	111835	intron_retention	2.363	0.348	206.4	135.1	Gs20800	asparaginyl-tRNA synthetase isoform 1
103	stig_61	81844	81903	intron_retention	2.383	0.300	61.6	100.3	Gs57760	S-formylglutathione hydrolase

104	stig_33	109707	109759	intron_retention	2.386	0.435	80.0	123.2		
105	stig_49	90831	90881	intron_retention	2.388	0.258	463.4	195.0	Gs50980	protochlorophyllide reductase
106	stig_54	39756	39806	intron_retention	2.395	0.407	246.0	212.9		
107	stig_51	98887	98941	intron_retention	2.397	0.251	163.2	182.1	Gs52250	dTMP kinase
108	stig_21	167799	167850	intron_retention	2.398	0.285	34.0	130.5	Gs28590	glutathione S-transferase isoform 1
109	stig_25	159484	159539	intron_retention	2.399	0.386	109.6	181.7	Gs32520	hypothetical protein Gasu_32520
110	stig_9	201442	201487	intron_retention	2.403	0.293	56.6	124.2	Gs15350	hypothetical protein isoform 1
111	stig_65	11096	11147	intron_retention	2.442	0.263	187.1	142.8		
112	stig_6	228567	228614	intron_retention	2.456	0.256	66.8	52.6	Gs11310	pfkB-type carbohydrate kinase family protein
113	stig_4	170809	170859	intron_retention	2.469	0.270	385.4	369.6	Gs08030	haloacid dehalogenaselike hydrolase
114	stig_57	58095	58148	intron_retention	2.503	0.304	111.6	83.5	Gs55490	adenylosuccinate synthase
115	stig_1	81228	81276	intron_retention	2.506	0.269	321.3	100.6		
116	stig_14	143729	143795	intron_retention	2.519	0.253	224.7	117.7	Gs21030	short chain dehydrogenase/reductase family oxidoreductase
117	stig_33	140745	140793	intron_retention	2.533	0.343	315.9	84.7	Gs38990	hypothetical protein Gasu_38990
118	stig_41	143859	143913	intron_retention	2.538	0.435	97.2	180.7	Gs45200	mitochondrial carrier (BOU / S-adenosylmethionine carrier)
119	stig_38	11095	11143	intron_retention	2.554	0.263	103.3	101.1	Gs42240	metal transporter, ACDP family
120	stig_17	179815	179870	intron_retention	2.572	0.330	139.0	82.7	Gs24510	hypothetical protein isoform 2
121	stig_36	87349	87428	intron_retention	2.574	0.290	36.2	272.4		
122	stig_46	120936	120993	intron_retention	2.576	0.250	311.9	315.2	Gs49070	guanine deaminase
123	stig_23	124464	124521	intron_retention	2.577	0.425	47.6	82.6		
124	stig_18	111262	111341	intron_retention	2.580	0.409	47.9	75.4		
125	stig_41	142803	142866	intron_retention	2.586	0.260	148.3	202.3	Gs45190	carboxyvinyl-carboxyphosphonate phosphorylmutase
126	stig_58	55087	55133	intron_retention	2.619	0.379	67.9	62.6	Gs56010	hypothetical protein Gasu_56010
127	stig_10	115240	115308	intron_retention	2.635	0.360	55.2	76.8	Gs16190	telomerase reverse transcriptase isoform 2
128	stig_319	2129	2199	intron_retention	2.647	0.268	80.7	63.4	Gs63560	hypothetical protein Gasu_63560, partial
129	stig_57	30291	30344	intron_retention	2.649	0.266	94.2	76.2	Gs55370	hypothetical protein Gasu_55370
130	stig_0	350262	350324	intron_retention	2.686	0.368	25.0	56.7	Gs01900	hypothetical protein isoform 1
131	stig_115	285	335	intron_retention	2.735	0.349	47.4	37.2		
132	stig_2	136766	136816	intron_retention	2.747	0.337	431.1	265.4	Gs04520	hypothetical protein Gasu_04520
133	stig_67	29436	29481	intron_retention	2.747	0.408	106.5	88.6		
134	stig_29	61947	61997	intron_retention	2.749	0.251	611.3	505.5	Gs35530	ubiquitin-conjugating enzyme E2
135	stig_0	74201	74252	intron_retention	2.760	0.270	77.3	93.3	Gs00420	NADH dehydrogenase I (Complex I) alpha subcomplex assembly factor1-like protein
136	stig_10	212218	212295	intron_retention	2.763	0.340	51.4	117.1		
137	stig_1	14610	14662	intron_retention	2.772	0.274	256.1	232.1	Gs02470	CTP synthase isoform 1
138	stig_68	10449	10506	intron_retention	2.978	0.365	107.1	83.5		
139	stig_26	97325	97375	intron_retention	2.986	0.283	30.3	60.2	Gs33070	MFS transporter, SP family, sugar:H+

										symporter
140	stig_49	51951	52005	intron_retention	3.021	0.311	295.4	283.7		
141	stig_28	179012	179067	intron_retention	3.046	0.275	321.4	419.4		
142	stig_27	164788	164841	intron_retention	3.046	0.321	131.0	50.5	Gs34300	short-chain dehydrogenase/reductase (SDR) family protein
143	stig_6	205935	205983	intron_retention	3.047	0.481	257.0	125.3	Gs11190	hypothetical protein Gasu_11190
144	stig_29	40985	41039	intron_retention	3.071	0.338	148.0	88.6	Gs35420	RNA polymerase primary sigma factor (Fig. 3C)
145	stig_33	141059	141115	intron_retention	3.081	0.397	208.1	63.1	Gs38990	hypothetical protein Gasu_38990
146	stig_3	134168	134230	intron_retention	3.141	0.383	233.0	141.9	Gs06130	phosphoglycerate mutase
147	stig_39	55890	55943	intron_retention	3.146	0.361	128.6	157.9	Gs43240	hypothetical protein Gasu_43240
148	stig_62	3024	3075	intron_retention	3.146	0.336	140.7	266.4		
149	stig_1	158384	158453	intron_retention	3.147	0.258	189.8	170.2		
150	stig_28	194696	194758	intron_retention	3.147	0.451	35.0	79.0		
151	stig_24	160234	160324	intron_retention	3.179	0.409	148.9	194.9		
152	stig_19	34722	34900	intron_retention	3.194	0.283	176.6	67.4		
153	stig_61	49483	49537	intron_retention	3.201	0.352	38.1	65.5		
154	stig_58	14083	14133	intron_retention	3.202	0.300	266.7	176.5	Gs55760	hypothetical protein Gasu_55760
155	stig_27	164633	164688	intron_retention	3.247	0.440	109.3	54.1	Gs34300	short-chain dehydrogenase/reductase (SDR) family protein
156	stig_46	17202	17256	intron_retention	3.260	0.359	143.5	213.7	Gs48470	AP-3 complex subunit sigma
157	stig_59	88870	88923	intron_retention	3.263	0.260	780.0	556.8	Gs56680	carbohydrate kinase family isoform 1
158	stig_33	37608	37680	intron_retention	3.343	0.452	152.0	137.4	Gs38480	mitochondrial carrier
159	stig_56	39410	39461	intron_retention	3.427	0.271	334.7	332.4	Gs54670	deoxyribodipyrimidine photo-lyase isoform 2
160	stig_0	389407	389457	intron_retention	3.445	0.269	98.7	117.4		
161	stig_41	124604	124658	intron_retention	3.448	0.255	163.0	308.0	Gs45080	hypothetical protein Gasu_45080
162	stig_45	128120	128188	intron_retention	3.502	0.393	61.9	42.4		
163	stig_54	96159	96229	intron_retention	3.526	0.404	41.3	38.9		
164	stig_16 2	3191	3235	intron_retention	3.578	0.275	173.2	173.4	Gs63220	hypothetical protein isoform 1
165	stig_48	92090	92147	intron_retention	3.603	0.265	59.1	156.9	Gs50280	hypothetical protein Gasu_50280
166	stig_11	168141	168202	intron_retention	3.618	0.507	42.5	84.7	Gs17780	NAD+ diphosphatase
167	stig_51	44846	44902	intron_retention	3.666	0.260	173.5	138.9		
168	stig_7	138220	138279	intron_retention	3.704	0.365	96.2	161.8	Gs12360	tubulin binding protein
169	stig_51	93825	93946	intron_retention	3.792	0.469	187.3	142.8	Gs52230	splice factor, putative isoform 1
170	stig_23	43773	43824	intron_retention	3.809	0.289	438.6	570.6	Gs29990	periplasmic divalent cation tolerance protein isoform 1
171	stig_61	81750	81800	intron_retention	3.816	0.286	84.6	120.6	Gs57760	S-formylglutathione hydrolase
172	stig_40	36535	36590	intron_retention	3.856	0.474	272.4	458.5	Gs43920	kinetochore protein Mis18 isoform 1
173	stig_37	114676	114730	intron_retention	3.866	0.391	68.7	80.9	Gs41950	ABC transporter, iron complex transport, substrate-binding protein
174	stig_18	39768	39820	intron_retention	4.002	0.306	207.1	318.3	Gs24800	hypothetical protein Gasu_24800
175	stig_8	171945	171995	intron_retention	4.003	0.423	109.3	23.3	Gs13930	methyltransferase
176	stig_10	135163	135234	intron_retention	4.053	0.527	24.7	62.4		

177	stig_40	36941	36998	intron_retention	4.102	0.418	159.4	204.8	Gs43920	kinetochore protein Mis18 isoform 1
178	stig_81	4506	4560	intron_retention	4.114	0.391	43.7	26.7	Gs61170	NAD-dependent epimerase/dehydratase
179	stig_32	89297	89361	intron_retention	4.150	0.271	385.2	151.2	Gs38070	beta-1,4-mannosyl-glycoproteinbeta-1,4-N-acetylglucosaminyltransferase
180	stig_58	69986	70037	intron_retention	4.703	0.294	84.5	94.9	Gs56070	alpha/beta fold family hydrolase
181	stig_25	34834	34906	intron_retention	4.775	0.446	331.8	405.4		
182	stig_47	120817	120871	intron_retention	4.806	0.253	150.8	160.4		
183	stig_49	98988	99048	intron_retention	4.842	0.295	68.1	383.4		
184	stig_8	49609	49659	intron_retention	4.874	0.315	186.8	181.7		
185	stig_49	110449	110501	intron_retention	4.969	0.294	74.9	148.6		
186	stig_49	46715	46782	intron_retention	5.015	0.405	103.9	125.3	Gs50780	peptidyl-prolyl cis-trans isomerase B (cyclophilin B)
187	stig_266	3416	3484	intron_retention	5.031	0.478	106.7	102.8		
188	stig_59	50475	50535	intron_retention	5.217	0.621	16.7	91.7	Gs56500	30S ribosomal protein S1
189	stig_51	81697	81748	intron_retention	5.279	0.481	52.2	51.8	Gs52190	AAA-type ATPase
190	stig_29	62076	62128	intron_retention	5.308	0.270	512.5	479.9	Gs35530	ubiquitin-conjugating enzyme E2
191	stig_8	211194	211248	intron_retention	5.312	0.368	49.3	84.9	Gs14110	hypothetical protein Gasu_14110
192	stig_10	79661	79728	intron_retention	5.416	0.602	350.3	76.8	Gs16000	ABC transporter, ATP-binding protein isoform 1
193	stig_62	52529	52586	intron_retention	5.448	0.275	241.8	175.4		
194	stig_149	333	403	intron_retention	5.460	0.477	39.3	73.7		
195	stig_29	149789	149838	intron_retention	5.472	0.382	331.1	66.8	Gs36040	hypothetical protein isoform 2
196	stig_18	48336	48389	intron_retention	5.485	0.268	339.8	772.8	Gs24850	short-chain dehydrogenase/reductase SDR isoform 2
197	stig_3	275067	275122	intron_retention	5.499	0.513	871.2	986.0		
198	stig_33	133292	133347	intron_retention	5.549	0.408	101.0	89.1	Gs38930	DNA ligase 4
199	stig_3	131757	131806	intron_retention	5.930	0.252	204.0	203.3		
200	stig_18	48473	48527	intron_retention	5.962	0.286	462.7	1052.4	Gs24850	short-chain dehydrogenase/reductase SDR isoform 2
201	stig_18	47847	47903	intron_retention	5.966	0.366	128.1	252.0	Gs24850	short-chain dehydrogenase/reductase SDR isoform 2
202	stig_10	55794	55850	intron_retention	5.993	0.519	88.5	120.5	Gs15900	H+-translocating PPase (vacuolar)
203	stig_27	165010	165063	intron_retention	6.032	0.412	1249.6	127.5	Gs34300	short-chain dehydrogenase/reductase (SDR) family protein
204	stig_18	48168	48230	intron_retention	6.230	0.320	253.7	629.1	Gs24850	short-chain dehydrogenase/reductase SDR isoform 2
205	stig_3	274961	275023	intron_retention	6.390	0.379	837.0	833.2		Novel ORF shown in Fig. 3C
206	stig_46	30923	30971	intron_retention	6.600	0.393	325.8	191.7	Gs48550	alpha/beta fold family hydrolase
207	stig_7	53341	53422	intron_retention	7.398	0.463	482.5	253.0	Gs11750	hypothetical protein Gasu_11750
208	stig_1	295474	295525	intron_retention	8.475	0.434	102.8	245.6	Gs03920	hypothetical protein Gasu_03920
209	stig_40	36692	36752	intron_retention	8.483	0.488	278.2	282.7	Gs43920	kinetochore protein Mis18 isoform 1
210	stig_1	281013	281073	intron_retention	9.978	0.443	138.3	66.1		
211	stig_10	115068	115121	intron_retention	10.842	0.324	85.6	98.6	Gs16190	telomerase reverse transcriptase isoform

212	stig_62	36555	36611	intron_retention	11.678	0.307	1450.0	1715.0	Gs57910	hypothetical protein Gasu_57910

[1] Statistic of differential ASM transcription. Larger value indicates greater difference (see Hu et al. 2013 for details).

[2] Square root of JSD between samples under heat condition and samples under cold condition. Larger value indicates great difference (see Hu et al. 2013 for details).
