# Supplementary material for: Unexpected conservation of the RNA splicing apparatus in the highly streamlined genome of Galdieria sulphuraria
Source: BMC Evol Biol. 2018 Apr 2;18:41. doi: 10.1186/s12862-018-1161-x (PMC5880011; doi:10.1186/s12862-018-1161-x)
Supplement: Supplementary file 16 — Figure S8. Intron retention in a Galdieria sulphuraria gene. (PDF 74 kb) [file 12862_2018_1161_MOESM16_ESM.pdf]

```
TGGATTCGCGCCAGCATATCCAAATATCTAAATGAATACAATCGCTCCGTTAGAGTGCCT exon3
 W  I  R  A  S  I  S  K  Y  L  N  E  Y  N  R  S  V  R  V  P
GCCAAGGTTCTTGCTCTTTTCAATCAGTCGAAAAAAGTCGCAGTCGAACTGGAAAGTAAG exon3
 A  K  V  L  A  L  F  N  Q  S  K  K  V  A  V  E  L  E  S  K
AAGAAAGTACCAAGTGAGAGTGAAGTCTGCTCGTTACTTGGTGTCTCAAGTTCCCGTCTT exon3
 K  K  V  P  S  E  S  E  V  C  S  L  L  G  V  S  S  S  R  L
CGCTTTTGTATAGAGgtatttccgtattctttttcatattgtgcacctccttatgtctaa intron3
 R  F  C  I  E  V  F  P  Y  S  F  S  Y  C  A  P  P  Y  V  *
tttgtagGCTGTTACTAACCAACCTGTTTCATTGGAAAGGTTAGGAGAGTTGTTAGAAGA exon4
 F  V  G  C  Y  *  P  T  C  F  I  G  K  V  R  R  V  V  R  R
TAGTGGACGTCTGGGTAAGGGGGCTGTGTATTGTGT…                        exon4
 *  W  T  S  G  *  G  G  C  V  L  C
```

Figure S8. Intron retention in a *Galdieria sulphuraria* gene. The retention of the 3rd intron leads to introduction of stop codon and truncation of exon 4-encoded peptide in a *G. sulphuraria* gene encoding RNA polymerase primary sigma factor. The exons 3 and 4 encode a part of the Sigma70-r3 domain (pfam04539). Intronic sequence is shown in grey color. Stop codons are denoted by the asterisks highlighted in green color.
