# Supplementary material for: Unexpected conservation of the RNA splicing apparatus in the highly streamlined genome of Galdieria sulphuraria
Source: BMC Evol Biol. 2018 Apr 2;18:41. doi: 10.1186/s12862-018-1161-x (PMC5880011; doi:10.1186/s12862-018-1161-x)
Supplement: Supplementary file 17 — Table S8. Galdieria sulphuraria genes that were differentially expressed under the heat and cold conditions. (PDF 186 kb) [file 12862_2018_1161_MOESM17_ESM.pdf]

Table S8. ***Galdieria sulphuraria*** **genes that were differentially expressed under the heat and cold conditions**

No.	Chr	Start	End	Stat_diff_trans[1]	Fold change	Coverage (heat)	Coverage (cold)	Gene	Product
1	stig_48	24951	26242	0.966	6.503	29.8	213.5	Gs49900	primary-amine oxidase
2	stig_38	15383	16464	1.080	0.375	393.7	148.0	Gs42260	3R-hydroxymyristoyl ACP dehydrase isoform 1
3	stig_54	16257	17900	1.082	0.395	869.4	360.3	Gs53580	40S ribosomal protein S24e
4	stig_31	156917	159161	1.088	2.649	112.9	301.9	Gs52900	hypothetical protein Gasu_52900, partial
5	stig_31	156917	159161	1.088	2.649	112.9	301.9	Gs37540	hypothetical protein Gasu_37540
6	stig_60	8012	9085	1.148	0.335	1624.5	609.7	Gs56750	hypothetical protein Gasu_56750
7	stig_2	171446	171997	1.317	0.305	2186.9	687.8	Gs04730	60S ribosomal protein L30e
8	stig_30	74731	75637	1.329	0.389	846.7	363.5	Gs36380	60S ribosomal protein L17e
9	stig_18	125882	128001	1.405	2.663	540.9	1377.8	Gs25340	hypothetical protein Gasu_25340
10	stig_32	44408	47068	1.438	0.389	1738.3	731.7	Gs37780	30S ribosomal protein S2 (plastid) isoform 1
11	stig_32	44408	47068	1.438	0.389	1738.3	731.7	Gs37790	glutamine synthetase
12	stig_25	125675	126825	1.517	0.257	1708.7	442.7	Gs32310	RNA-binding protein
13	stig_10	25816	28420	1.559	0.370	268.4	99.3	Gs15740	H+-translocating PPase (vacuolar)
14	stig_20	104016	105029	1.632	0.398	287.9	115.7	Gs27100	signal peptidase I isoform 2
15	stig_62	47378	50492	1.716	3.504	134.8	756.0	Gs57980	glycerol dehydrogenase
16	stig_62	47378	50492	1.716	3.504	134.8	756.0	Gs57970	alcohol dehydrogenase
17	stig_11	102392	105339	1.724	0.359	98.9	35.8	Gs17400	O-methyltransferase
18	stig_11	102392	105339	1.724	0.359	98.9	35.8	Gs17390	RNA polymerase primary sigma factor
19	stig_4	105431	109027	1.832	3.305	193.6	379.8	Gs07710	hypothetical protein Gasu_07710
20	stig_4	105431	109027	1.832	3.305	193.6	379.8	Gs07720	phospholipid-translocating P-type ATPase
21	stig_0	356068	358613	1.835	0.356	67.7	20.6	Gs01940	hypothetical protein isoform 1
22	stig_11	141745	143378	1.837	0.285	2357.6	678.2	Gs17630	actin
23	stig_21	82182	84585	1.872	0.292	810.2	324.3	Gs28130	**splicing factor, arginine/serine-rich 2***
24	stig_21	82182	84585	1.872	0.292	810.2	324.3	Gs28120	hypothetical protein Gasu_28120
25	stig_9	152964	154926	1.880	0.252	323.5	86.9	Gs15110	dihydrolipoamide dehydrogenase
26	stig_53	14376	16064	1.904	0.390	277.1	99.7	Gs52990	translation initiation factor eIF-3 subunit 9
27	stig_27	121140	122886	1.910	0.310	834.0	258.3	Gs34040	3-isopropylmalate dehydrogenase
28	stig_11	98479	100286	1.918	0.317	56.7	89.1	Gs17370	translation initiation factor eIF-5A
29	stig_11	98479	100286	1.918	0.317	56.7	89.1	Gs17360	short-chain dehydrogenase/reductase SDR
30	stig_23	143401	146017	1.925	4.085	77.5	150.4	Gs30610	alpha-1,4-N-acetylglucosaminyltransferase EXTL3
31	stig_45	67853	70660	2.005	0.398	170.5	65.1	Gs48070	H3/H4 histone acetyltransferase
32	stig_45	67853	70660	2.005	0.398	170.5	65.1	Gs48080	hypothetical protein Gasu_48080
33	stig_230	19	1005	2.054	0.355	614.5	213.6	Gs64140	translationally controlled tumor protein (TCTP) (p23)-like protein
34	stig_230	19	1005	2.054	0.355	614.5	213.6	Gs64610	translationally controlled tumor protein (TCTP) (p23)-like protein
35	stig_30	86383	88657	2.054	0.350	257.4	90.8	Gs36460	glutamate dehydrogenase (NAD(P)+) isoform 2
36	stig_8	226713	231812	2.056	0.374	189.6	99.6	Gs14230	3-oxoacyl-[acyl-carrier-protein] synthase II
37	stig_8	226713	231812	2.056	0.374	189.6	99.6	Gs14220	magnesium chelatase subunit D
38	stig_8	226713	231812	2.056	0.374	189.6	99.6	Gs14210	molybdenum cofactor biosynthesis protein E

39	stig_7	63534	64339	2.057	0.165	119.5	19.9	Gs11850	endonuclease
40	stig_61	15950	17053	2.057	0.322	2311.9	763.3	Gs57310	40S ribosomal protein S12e
41	stig_15	183919	185007	2.166	4.295	101.2	451.5	Gs22200	hypothetical protein Gasu_22200
42	stig_4	138201	139259	2.185	2.800	1978.8	5545.8	Gs07860	hypothetical protein Gasu_07860
43	stig_25	79785	81911	2.232	3.972	795.1	3406.2	Gs32120	hypothetical protein Gasu_32120
44	stig_31	111776	113794	2.258	0.384	329.1	150.9	Gs37250	60S ribosomal protein L38e
45	stig_31	111776	113794	2.258	0.384	329.1	150.9	Gs37260	threonine 3-dehydrogenase
46	stig_53	4200	5922	2.273	0.323	737.6	241.2	Gs52930	hypothetical protein Gasu_52930
47	stig_13	115000	118731	2.310	0.330	433.5	222.7	Gs19870	hypothetical protein Gasu_19870
48	stig_13	115000	118731	2.310	0.330	433.5	222.7	Gs19860	RNA methyltransferase, TrmH family
49	stig_13	115000	118731	2.310	0.330	433.5	222.7	Gs19840	elongation factor EF-1 alpha subunit
50	stig_13	115000	118731	2.310	0.330	433.5	222.7	Gs19850	hypothetical protein Gasu_19850
51	stig_49	97635	99453	2.344	91.153	38.6	2869.2	Gs51030	hypothetical protein Gasu_51030
52	stig_49	97635	99453	2.344	91.153	38.6	2869.2	Gs51040	hypothetical protein Gasu_51040
53	stig_37	32573	33786	2.346	0.210	82.8	17.8	Gs41520	hypothetical protein Gasu_41520
54	stig_65	255	2976	2.363	0.261	329.7	135.0	Gs59090	phosphoserine phosphatase
55	stig_65	255	2976	2.363	0.261	329.7	135.0	Gs59080	dTDP-glucose 4,6-dehydratase, partial
56	stig_47	34726	35737	2.405	3.381	223.8	780.8	Gs49350	small GTP-binding protein of Ras family
57	stig_27	164097	166221	2.416	0.137	87.2	31.6	Gs34300	short-chain dehydrogenase/reductase (SDR) family protein
58	stig_27	164097	166221	2.416	0.137	87.2	31.6	Gs34310	hypothetical protein Gasu_34310
59	stig_26	50815	52448	2.479	0.400	199.2	80.0	Gs32830	hypothetical protein Gasu_32830
60	stig_15	178910	181099	2.504	0.277	82.8	18.4	Gs22170	hypothetical protein Gasu_22170
61	stig_51	78815	80107	2.508	0.378	380.3	147.5	Gs52180	hypothetical protein isoform 2
62	stig_33	114306	115008	2.519	3.766	118.2	447.7	Gs38860	hypothetical protein Gasu_38860
63	stig_11	109393	110373	2.522	0.385	1754.2	683.8	Gs17430	poly(A) binding protein isoform 1
64	stig_3	111486	114427	2.563	0.395	280.3	152.7	Gs05980	pyruvate kinase
65	stig_3	111486	114427	2.563	0.395	280.3	152.7	Gs06000	40S ribosomal protein S17
66	stig_3	111486	114427	2.563	0.395	280.3	152.7	Gs05990	5-formyltetrahydrofolate cyclo-ligase
67	stig_27	149857	153407	2.580	0.348	259.6	112.0	Gs34220	40S ribosomal protein S6e
68	stig_27	149857	153407	2.580	0.348	259.6	112.0	Gs34230	**ATP-dependent RNA helicase***
69	stig_27	149857	153407	2.580	0.348	259.6	112.0	Gs34210	mitochondrial carrier, adenine nucleotidetranslocator
70	stig_49	3878	4470	2.612	18.966	76.0	1451.6	Gs50510	hypothetical protein Gasu_50510
71	stig_50	45779	47440	2.658	2.541	71.8	165.9	Gs51320	O-acyl transferase, membrane bound
72	stig_4	103587	105137	2.737	2.834	460.9	1311.2	Gs07700	putative acetate transporter
73	stig_62	82822	84310	2.739	0.255	198.0	53.5	Gs58190	UDPglucose 6-dehydrogenase
74	stig_5	1881	3561	2.741	0.389	230.5	111.5	Gs08640	hypothetical protein Gasu_08640
75	stig_5	1881	3561	2.741	0.389	230.5	111.5	Gs08650	hypothetical protein Gasu_08650
76	stig_7	25386	26257	2.743	10.162	69.4	702.1	Gs11660	hypothetical protein Gasu_11660
77	stig_3	263364	266264	2.755	0.370	1107.2	405.0	Gs06940	60S ribosomal protein L23Ae
78	stig_3	263364	266264	2.755	0.370	1107.2	405.0	Gs06950	30S ribosomal protein S5 (mitochondria)
79	stig_47	108366	109850	2.811	2.816	110.7	337.8	Gs49740	hypothetical protein Gasu_49740
80	stig_47	108366	109850	2.811	2.816	110.7	337.8	Gs49730	CDP-alcohol phosphatidyltransferase
81	stig_4	200824	202542	2.818	0.225	756.1	175.4	Gs08210	adenosine kinase
82	stig_8	176621	178699	2.873	3.376	200.1	615.2	Gs13970	zinc finger protein

83	stig_8	176621	178699	2.873	3.376	200.1	615.2	Gs13960	plastid-lipid associated protein PAP, putative
84	stig_411	875	1376	2.876	7.958	31.3	240.3	Gs66120	hypothetical protein Gasu_66120
85	stig_6	3056	4088	2.908	233.860	23.4	5537.3	Gs10210	hypothetical protein Gasu_10210
86	stig_17	55687	57099	2.912	3.021	36.8	98.0	Gs23850	hypothetical protein Gasu_23850
87	stig_299	13	1455	2.914	3.170	359.0	1048.7	Gs65160	hypothetical protein Gasu_65160, partial
88	stig_299	13	1455	2.914	3.170	359.0	1048.7	Gs65170	hypothetical protein Gasu_65170
89	stig_250	48	838	2.933	3.417	777.1	2775.5	Gs64440	light-harvesting complex protein
90	stig_250	48	838	2.933	3.417	777.1	2775.5	Gs15230	light-harvesting complex protein
91	stig_38	111403	114304	2.964	2.559	63.8	165.0	Gs42810	bifunctional polynucleotide phosphatase/kinase
92	stig_38	111403	114304	2.964	2.559	63.8	165.0	Gs42830	bZIP transcription factor isoform 1
93	stig_38	111403	114304	2.964	2.559	63.8	165.0	Gs42820	hypothetical protein Gasu_42820
94	stig_28	124000	124822	3.012	3.396	3036.0	10397.9	Gs34810	hypothetical protein Gasu_34810
95	stig_3	34025	39524	3.111	8.816	54.9	440.9	Gs05590	hypothetical protein Gasu_05590
96	stig_3	34025	39524	3.111	8.816	54.9	440.9	Gs05580	hydrolase
97	stig_3	34025	39524	3.111	8.816	54.9	440.9	Gs05610	IMP dehydrogenase
98	stig_3	34025	39524	3.111	8.816	54.9	440.9	Gs05600	hypothetical protein Gasu_05600
99	stig_3	34025	39524	3.111	8.816	54.9	440.9	Gs05570	RNA-binding S1 domain-containing protein
100	stig_10	77962	80491	3.114	0.110	87.8	6.6	Gs16010	3-oxo-5-alpha-steroid 4-dehydrogenase 3 isoform 1
101	stig_10	77962	80491	3.114	0.110	87.8	6.6	Gs16000	ABC transporter, ATP-binding protein isoform 1
102	stig_22	156580	158207	3.190	7.645	300.3	2260.4	Gs29650	hypothetical protein Gasu_29650
103	stig_22	156580	158207	3.190	7.645	300.3	2260.4	Gs29660	hypothetical protein Gasu_29660
104	stig_47	33861	34574	3.205	0.378	1161.4	455.3	Gs49340	40S ribosomal protein S15e
105	stig_8	152960	155103	3.287	0.171	882.7	223.8	Gs13840	potassium channel tetramerisation domain-like protein
106	stig_8	152960	155103	3.287	0.171	882.7	223.8	Gs13830	glycine-rich RNA binding protein isoform 2
107	stig_5	266873	269320	3.361	0.332	175.4	47.5	Gs10100	ADP-ribosylation factor isoform 2
108	stig_5	266873	269320	3.361	0.332	175.4	47.5	Gs10090	hypothetical protein Gasu_10090
109	stig_5	266873	269320	3.361	0.332	175.4	47.5	Gs10080	exosome complex component MTR3, animal type
110	stig_23	132120	133699	3.388	0.157	679.6	101.4	Gs30520	hypothetical protein Gasu_30520
111	stig_32	97446	99345	3.408	0.215	1015.1	232.3	Gs38110	hypothetical protein Gasu_38110
112	stig_41	147226	147961	3.481	5.191	94.8	500.0	Gs45230	hypothetical protein Gasu_45230
113	stig_69	23323	24848	3.547	3.281	116.9	305.9	Gs60480	methylsterol monooxygenase
114	stig_69	23323	24848	3.547	3.281	116.9	305.9	Gs60490	hypothetical protein isoform 2
115	stig_12	173531	174031	3.733	0.293	1012.0	319.7	Gs19110	60S ribosomal protein L34e
116	stig_16	32063	33182	3.746	2.678	115.3	319.3	Gs22530	Nudix hydrolase-like protein
117	stig_16	120810	125307	3.788	3.594	256.7	674.3	Gs23060	hypothetical protein Gasu_23060
118	stig_16	120810	125307	3.788	3.594	256.7	674.3	Gs23070	hypothetical protein Gasu_23070
119	stig_16	120810	125307	3.788	3.594	256.7	674.3	Gs23050	hypothetical protein Gasu_23050
120	stig_5	111215	113757	3.833	2.842	83.0	211.0	Gs09280	short-chain dehydrogenase/reductase SDR
121	stig_5	111215	113757	3.833	2.842	83.0	211.0	Gs09290	hypothetical protein Gasu_09290
122	stig_31	133588	136016	3.836	0.358	2810.5	1031.3	Gs37380	beta-1,4-N-acetylglucosaminyltransferase
123	stig_31	133588	136016	3.836	0.358	2810.5	1031.3	Gs37390	glycine-rich RNA binding protein isoform 1
124	stig_74	539	3740	3.906	3.546	162.6	636.2	Gs60960	nucleobase:cation symporter-1, NCS1 family, partial
125	stig_74	539	3740	3.906	3.546	162.6	636.2	Gs60980	NAD-dependent formate dehydrogenase
126	stig_74	539	3740	3.906	3.546	162.6	636.2	Gs60970	hypothetical protein Gasu_60970

127	stig_18	116608	123590	3.929	0.193	572.0	253.6	Gs25260	hypothetical protein Gasu_25260
128	stig_18	116608	123590	3.929	0.193	572.0	253.6	Gs25300	glycerophosphodiester phosphodiesterase
129	stig_18	116608	123590	3.929	0.193	572.0	253.6	Gs25280	hypothetical protein Gasu_25280
130	stig_18	116608	123590	3.929	0.193	572.0	253.6	Gs25270	phosphatidate cytidylyltransferase/ phytol kinase
131	stig_18	116608	123590	3.929	0.193	572.0	253.6	Gs25310	hypothetical protein Gasu_25310
132	stig_18	116608	123590	3.929	0.193	572.0	253.6	Gs25320	mitochondrial ribosomal protein L23 precursor
133	stig_18	116608	123590	3.929	0.193	572.0	253.6	Gs25290	ubiquitin carboxyl-terminal hydrolase L5
134	stig_34	166921	168103	4.026	3.103	185.6	530.2	Gs39930	2-hydroxyglutarate dehydrogenase isoform 1
135	stig_26	122173	124962	4.070	0.353	178.9	93.2	Gs33180	splicing factor, arginine/serine-rich 7
136	stig_26	122173	124962	4.070	0.353	178.9	93.2	Gs33170	hypothetical protein Gasu_33170
137	stig_23	35924	38611	4.089	3.748	73.2	325.8	Gs29950	poly(A) binding protein
138	stig_23	35924	38611	4.089	3.748	73.2	325.8	Gs29960	hypothetical protein isoform 1
139	stig_303	3321	3840	4.193	3.602	198.6	739.0	Gs65230	MA3 domain-containing protein
140	stig_303	3321	3840	4.193	3.602	198.6	739.0	Gs51550	MA3 domain-containing protein
141	stig_7	83599	84749	4.490	3.576	93.2	295.3	Gs11960	hypothetical protein Gasu_11960
142	stig_50	82505	84635	4.547	0.225	629.0	156.2	Gs51510	DNA damage-binding protein 2
143	stig_50	82505	84635	4.547	0.225	629.0	156.2	Gs51500	hypothetical protein Gasu_51500
144	stig_5	154137	157351	4.675	0.319	761.5	242.6	Gs09490	bifunctional acetyl-CoA carboxylase/biotin carboxylase subunitisoform 2
145	stig_5	154137	157351	4.675	0.319	761.5	242.6	Gs09500	GTP-binding protein
146	stig_7	206909	208477	4.873	2.859	97.0	361.8	Gs12750	hypothetical protein Gasu_12750
147	stig_7	206909	208477	4.873	2.859	97.0	361.8	Gs12740	4a-hydroxytetrahydrobiopterin dehydratase
148	stig_24	153809	158573	5.219	0.394	689.1	229.6	Gs31560	H+-transporting P-type ATPase
149	stig_24	153809	158573	5.219	0.394	689.1	229.6	Gs31580	alanine--glyoxylate transaminase/serine--pyruvateaminotransferase
150	stig_24	153809	158573	5.219	0.394	689.1	229.6	Gs31570	arsenite efflux protein, ArsB family
151	stig_61	6456	8274	5.270	0.383	1057.2	437.2	Gs57280	hypothetical protein Gasu_57280
152	stig_61	6456	8274	5.270	0.383	1057.2	437.2	Gs57270	60S ribosomal protein L15e isoform 2
153	stig_59	50339	53509	5.310	0.228	2203.3	537.2	Gs56500	30S ribosomal protein S1
154	stig_59	50339	53509	5.310	0.228	2203.3	537.2	Gs56510	myb domain-containing protein
155	stig_23	157110	157828	5.707	0.338	1417.1	490.1	Gs30690	peptidylprolyl isomerase
156	stig_6	79260	81255	5.747	0.362	505.5	184.3	Gs10570	DNA gyrase subunit A
157	stig_51	51204	52945	5.758	0.236	1158.6	288.4	Gs52020	40S ribosomal protein S16e
158	stig_7	144323	145151	5.924	3.158	173.5	553.5	Gs12410	hypothetical protein Gasu_12410
159	stig_19	106507	107282	6.018	0.383	1292.0	495.3	Gs26210	60S ribosomal protein L23e
160	stig_56	84315	89106	6.176	2.537	97.9	220.5	Gs54980	ubiquitin-conjugating enzyme E2
161	stig_56	84315	89106	6.176	2.537	97.9	220.5	Gs54970	mRNA (2'-O-methyladenosine-N6-)-methyltransferase
162	stig_56	84315	89106	6.176	2.537	97.9	220.5	Gs54990	hypothetical protein Gasu_54990
163	stig_28	141958	144419	6.278	0.117	3025.4	359.9	Gs34920	5-methyltetrahydropteroyltriglutamate--homocysteinemethyltransferase
164	stig_1	291963	293489	6.304	0.349	1066.1	460.8	Gs03900	S-adenosylmethionine synthetase
165	stig_15	36070	37326	6.768	2.590	172.7	467.6	Gs21360	fatty acid hydroxylase
166	stig_5	109911	110219	6.897	7.391	9511.8	70691.5	Gs09270	hypothetical protein Gasu_09270
167	stig_62	44932	47256	7.766	8.582	438.8	3826.1	Gs57960	glycerol dehydrogenase

168	stig_62	44932	47256	7.766	8.582	438.8	3826.1	Gs57950	putative acetate transporter
169	stig_19	56804	58791	7.909	0.261	430.1	111.8	Gs25940	cell division control protein 42
170	stig_57	36060	38768	8.282	2.975	90.9	255.9	Gs55410	transcription factor
171	stig_57	36060	38768	8.282	2.975	90.9	255.9	Gs55420	FAD-dependent monooxygenase/oxidoreductase acting on aromaticcompound isoform 2
172	stig_92	309	1924	10.941	0.329	1307.5	434.8	Gs61400	40S ribosomal protein S11e
173	stig_92	309	1924	10.941	0.329	1307.5	434.8	Gs61410	hypothetical protein Gasu_61410
174	stig_49	71383	74165	15.635	0.218	1509.2	338.0	Gs50900	hypothetical protein Gasu_50900
175	stig_49	71383	74165	15.635	0.218	1509.2	338.0	Gs50890	60S ribosomal protein L37e
176	stig_37	107794	111733	16.308	0.397	582.3	264.4	Gs41930	flavodoxin family protein
177	stig_37	107794	111733	16.308	0.397	582.3	264.4	Gs41910	hypothetical protein Gasu_41910
178	stig_37	107794	111733	16.308	0.397	582.3	264.4	Gs41920	hypothetical protein Gasu_41920

[1] Statistic of differential gene expression, larger value indicates greater difference (see Hu et al. 2013 for details).

* Spliceosomal genes are shown in bold face.
