# Supplementary material for: Unexpected conservation of the RNA splicing apparatus in the highly streamlined genome of Galdieria sulphuraria
Source: BMC Evol Biol. 2018 Apr 2;18:41. doi: 10.1186/s12862-018-1161-x (PMC5880011; doi:10.1186/s12862-018-1161-x)
Supplement: Supplementary file 18 — Figure S9. Phylogenetic trees of UPF1, UPF2, and UPF3. (PDF 97 kb) [file 12862_2018_1161_MOESM18_ESM.pdf]

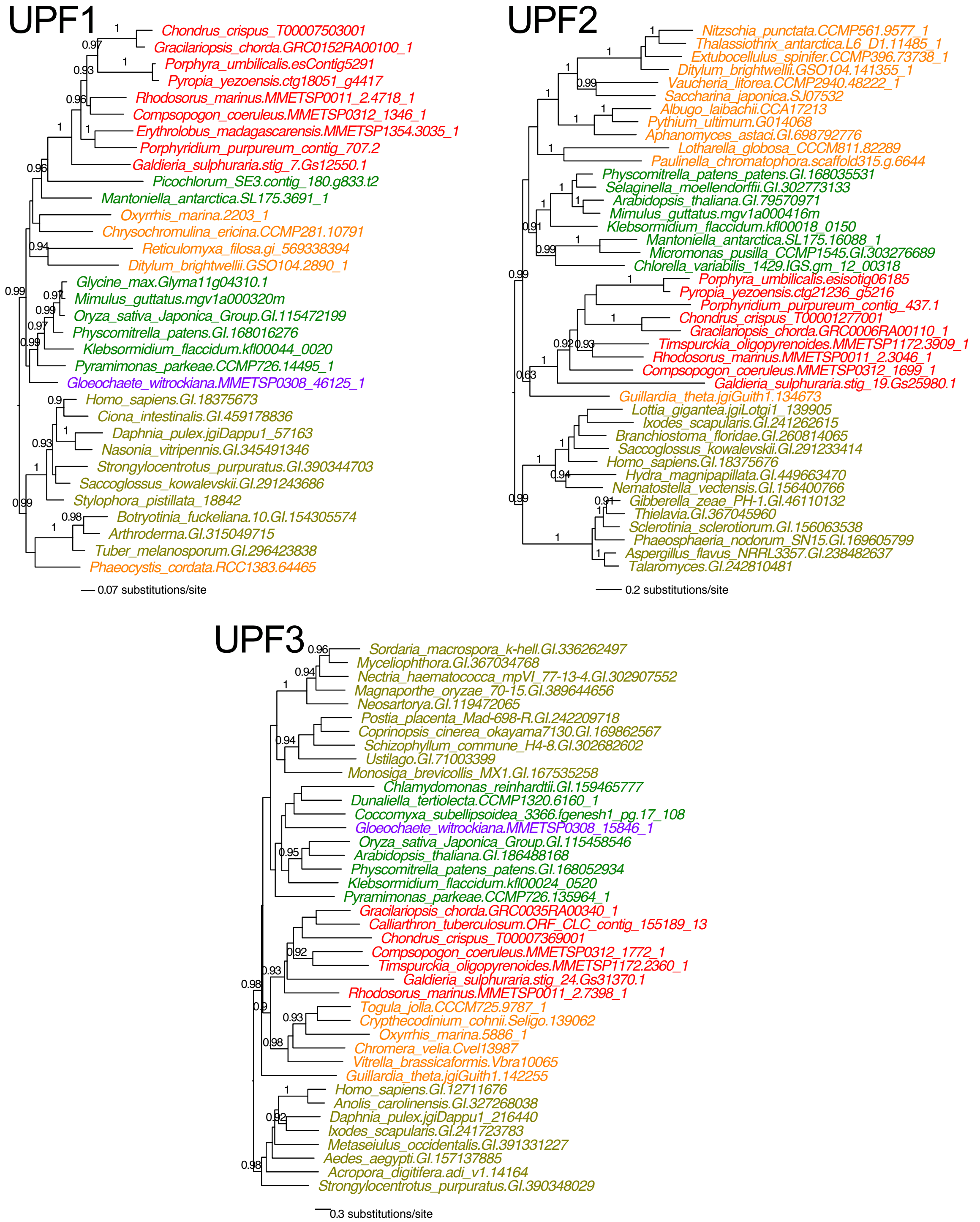

Figure S9. Phylogenetic trees of UPF1, UPF2, and UPF3. The trees are built using FastTree (13) with branch support values (shown when larger than 0.9) estimated using the Shimodaira-Hasegawa test. Species are shown in difference colors including red (red algae), green (Viridiplantae), glaucophytes (purple), orange (chromalveolates), and Metazoa and fungi (brown).
