# Supplementary material for: Unexpected conservation of the RNA splicing apparatus in the highly streamlined genome of Galdieria sulphuraria
Source: BMC Evol Biol. 2018 Apr 2;18:41. doi: 10.1186/s12862-018-1161-x (PMC5880011; doi:10.1186/s12862-018-1161-x)
Supplement: Supplementary file 19 — Supplementary Methods. (PDF 109 kb) [file 12862_2018_1161_MOESM19_ESM.pdf]

Supplementary Methods

Red algal genome and transcriptome data

We included into our analysis the genome data from *Chondrus crispus* [1], *Gracilariopsis chorda* (unpublished data), *Rhodosorus marinus* [2], *Galdieria phlegrea* [3], *G. sulphuraria* [4], *Cyanidioschyzon merolae* [5], Bangiacea, and *Porphyridium*. When analyzing Bangiaceae data, we pooled protein sequences that were derived from *Pyropia yezoensis* genome [6] and *Porphyra umbilicalis* transcriptome [7]. Likewise, we pooled protein sequences derived from *Porphyridium purpureum* genome [8] and *P. aerugineum* transcriptome [2]. The *G. chorda* genome (89.6 Mb) was sequenced up to 80x coverage on PacBio sequencing platform. A total of 9.9K gene models were predicted under the guidance of an extensive transcriptome data (a total of 29.4 Gbp from 7 conditions, unpublished).

Non-redundant human spliceosomal protein set

We downloaded 244 experimentally verified human spliceosome machinery (SM)-associated proteins from an existing study [9]. These SM proteins were used as queries to search against a local protein database containing NCBI RefSeq (v58) and a comprehensive collection of red algal sequences [10] using BLASTp (*e*-value cutoff = 1e-5). For each query, the top 80 hits were retrieved, combined with the query sequences, and then aligned using MUSCLE (v3.8.31) [11] under the default setting. The alignment was trimmed using TrimAl (version 1.2) [12] in the automated mode (-automated1). The phylogenetic tree was constructed using FastTree (version 2.1.7) [13] under the 'WAG+CAT' model with 4 rounds of minimum evolution SPR moves (-psr 4) and exhaustive ML nearest-neighbor interchanges (-mlacc 2, -slownni). Branch support was derived from the Shimodaira-Hasegawa test [14].

For each SM gene tree, we inferred the largest metazoan monophyletic group (containing the human query gene) with ≥85% support (by SH-test). The collection of members of the monophyletic group was considered as an orthologous gene family. We then merged all 244 such orthologous gene families using a single-linkage method. In other words, two gene families (likely resulting from metazoan-specific duplications) were merged if they contained one or more shared members. This procedure led to 215 non-redundant orthologous gene families. For each gene family, a representative human protein was randomly selected for further analyses.

Homology-based SM gene finding

To reduce the impact of false negatives in the existing red algal proteome data (i.e., annotated gene models collected from previously studies), we took extra effort to search the potential SM proteins in the 8 red algal lineages (shown in Fig. 1B) with the following procedure. 1) The human SM proteins were used as queries to search against the red algal DNA databases using tBLASTn (*e*-value cutoff = 1e-5). The homologous proteins encoded in the red algal DNA sequences were subtracted from the tBLASTn outputs. 2) We recorded for each red algal species the genomic regions (exon regions) encoding homology to the human SM proteins from tBLASTn outputs. The genomic regions (corresponding to the same SM query proteins) less than 1 Kb apart were connected and merged into larger regions. The resulting genomic regions and the corresponding human SM proteins were then used for homology-based gene prediction using GeneWise [15]. All predicted proteins with scores (≥25) were collected. 3) The tBLASTn search- and GeneWise-derived proteins were combined with the existing proteome data from each species. The redundant sequences with identity (>85%) were removed using CD-HIT (v4.5.4) [16]. The resulting red algal proteome data were retained for SM gene search.

References

1. Collén J, Porcel B, Carré W, Ball SG, Chaparro C, Tonon T, et al. Genome structure and metabolic features in the red seaweed *Chondrus crispus* shed light on evolution of the Archaeplastida. Proc. Natl. Acad. Sci. U. S. A. 2013;110:5247–52.

2. Keeling PJ, Burki F, Wilcox HM, Allam B, Allen EE, Amaral-Zettler LA, et al. The Marine Microbial Eukaryote Transcriptome Sequencing Project (MMETSP): illuminating the functional diversity of eukaryotic life in the oceans through transcriptome sequencing. PLoS Biol. 2014;12:e1001889.

3. Qiu H, Price DC, Weber APM, Reeb V, Yang EC, Lee JM, et al. Adaptation through horizontal gene transfer in the cryptoendolithic red alga *Galdieria phlegrea*. Curr. Biol. 2013;23:R865-866.

4. Schönknecht G, Chen W-H, Ternes CM, Barbier GG, Shrestha RP, Stanke M, et al. Gene transfer from bacteria and archaea facilitated evolution of an extremophilic eukaryote. Science. 2013;339:1207–10.

5. Matsuzaki M, Misumi O, Shin-I T, Maruyama S, Takahara M, Miyagishima S-Y, et al. Genome sequence of the ultrasmall unicellular red alga *Cyanidioschyzon merolae* 10D. Nature. 2004;428:653–7.

6. Nakamura Y, Sasaki N, Kobayashi M, Ojima N, Yasuike M, Shigenobu Y, et al. The first symbiont-free genome sequence of marine red alga, Susabi-nori (*Pyropia yezoensis*). PloS One. 2013;8:e57122.

7. Chan CX, Blouin NA, Zhuang Y, Zäuner S, Prochnik SE, Lindquist E, et al. *Porphyra* (Bangiophyceae) transcriptomes provide insights into red algal development and metabolism. J. Phycol. 2012;48:1328–42.

8. Bhattacharya D, Price DC, Chan CX, Qiu H, Rose N, Ball S, et al. Genome of the red alga *Porphyridium purpureum*. Nat. Commun. 2013;4:1941.

9. Hegele A, Kamburov A, Grossmann A, Sourlis C, Wowro S, Weimann M, et al. Dynamic protein-protein interaction wiring of the human spliceosome. Mol. Cell. 2012;45:567–80.

10. Qiu H, Price DC, Yang EC, Yoon HS, Bhattacharya D. Evidence of ancient genome reduction in red algae (Rhodophyta). J. Phycol. 2015;51:624–36.

11. Edgar RC. MUSCLE: a multiple sequence alignment method with reduced time and space complexity. BMC Bioinformatics. 2004;5:113.

12. Capella-Gutiérrez S, Silla-Martínez JM, Gabaldón T. trimAl: a tool for automated alignment trimming in large-scale phylogenetic analyses. Bioinforma. Oxf. Engl. 2009;25:1972–3.

13. Price MN, Dehal PS, Arkin AP. FastTree 2--approximately maximum-likelihood trees for large alignments. PloS One. 2010;5:e9490.

14. Shimodaira, H., Hasegawa, M. Multiple comparisons of log-likelihoods with applications to phylogenetic inference. Mol. Biol. Evol. 1999;16:1114–6.

15. Birney E, Clamp M, Durbin R. GeneWise and Genomewise. Genome Res. 2004;14:988–95.

16. Li W, Godzik A. Cd-hit: a fast program for clustering and comparing large sets of protein or nucleotide sequences. Bioinforma. Oxf. Engl. 2006;22:1658–9.
